# Lysosomal genes contribute to Parkinson’s disease near agriculture with high intensity pesticide use

**DOI:** 10.1038/s41531-024-00703-4

**Published:** 2024-04-25

**Authors:** Kathie J. Ngo, Kimberly C. Paul, Darice Wong, Cynthia D. J. Kusters, Jeff M. Bronstein, Beate Ritz, Brent L. Fogel

**Affiliations:** 1 Department of Neurology, David Geffen School of Medicine, University of California Los Angeles, Los Angeles, CA 90095 USA; 2 Clinical Neurogenomics Research Center, David Geffen School of Medicine, University of California Los Angeles, Los Angeles, CA 90095 USA; 3 Department of Epidemiology, UCLA Fielding School of Public Health, Los Angeles, CA USA; 4 Department of Environmental Health Sciences, UCLA Fielding School of Public Health, Los Angeles, CA USA; 5 Department of Human Genetics, David Geffen School of Medicine, University of California Los Angeles, Los Angeles, CA 90095 USA

**Keywords:** Parkinson's disease, Genomics, Risk factors

## Abstract

Parkinson’s disease (PD), the second most common neurodegenerative disorder, develops sporadically, likely through a combination of polygenic and environmental factors. Previous studies associate pesticide exposure and genes involved in lysosomal function with PD risk. We evaluated the frequency of variants in lysosomal function genes among patients from the Parkinson’s, Environment, and Genes (PEG) study with ambient pesticide exposure from agricultural sources. 757 PD patients, primarily of White European/non-Hispanic ancestry (75%), were screened for variants in 85 genes using a custom amplicon panel. Variant enrichment was calculated against the Genome Aggregation Database (gnomAD). Enriched exonic variants were prioritized by exposure to a cluster of pesticides used on cotton and severity of disease progression in a subset of 386 patients subdivided by race/ethnicity. Gene enrichment analysis identified 36 variants in 26 genes in PEG PD patients. Twelve of the identified genes (12/26, 46%) had multiple enriched variants and/or a single enriched variant present in multiple individuals, representing 61% (22/36) of the observed variation in the cohort. The majority of enriched variants (26/36, 72%) were found in genes contributing to lysosomal function, particularly autophagy, and were bioinformatically deemed functionally deleterious (31/36, 86%). We conclude that, in this study, variants in genes associated with lysosomal function, notably autophagy, were enriched in PD patients exposed to agricultural pesticides suggesting that altered lysosomal function may generate an underlying susceptibility for developing PD with pesticide exposure. Further study of gene-environment interactions targeting lysosomal function may improve understanding of PD risk in individuals exposed to pesticides.

## Introduction

Parkinson’s Disease (PD) is the second most common neurodegenerative disease after Alzheimer’s disease, and the prevalence is expected to increase with an aging populance^1,2^. While the etiology of sporadic PD is certainly multifactorial and includes both genetic and environmental factors^3^, extensive genetic studies have resulted in the discovery of several gene mutations that can cause familial PD or polymorphisms that can alter risk and have provided us with some valuable insight into the pathogenesis of the disease. Despite these advances, genetics alone only accounts for the minority of cases and cannot explain the increasing incidence of PD^4^.

Abnormal protein homeostasis appears central to the pathogenesis of PD. Recent evidence has spotlighted altered autophagy as an important pathological pathway in the initiation and/or propagation of alpha-synuclein based on the ever-expanding list of relatively rare gene mutations associated with PD patients and within experimental animal models^5,6^. The importance of autophagic function in PD etiology is underscored by the obligatory presence of Lewy-body aggregates of alpha-synuclein in PD and the finding that lysosomal gene mutations can cause PD. For example, one mutation in the *GBA1* gene encoding glucocerebrosidase (GCase) results in an 8-fold increase in the risk of developing PD whereas carriers of two mutant alleles develop Gaucher’s disease^7^. Approximately 15% of Ashkenazi Jews with PD carry a *GBA1* mutation^7^. Most mutations in *GBA1* are believed to result in loss of function and reduced GCase activity can (but not always) lead to the accumulation of specific forms of higher molecular weight species of alpha-synuclein and autophagic dysfunction^8–10^. This is just one example that implicates alterations in autophagy in promoting pathological alpha-synuclein aggregation and the risk of developing PD. There are other rare mutations in lysosomal genes associated with PD as well^5,11^. Taken together, altered autophagy may be a common pathological pathway leading to the development of PD.

Epidemiologic studies have long shown that pesticide exposure is a risk factor for PD^1,12^. To better understand genetic factors influencing PD risk associated with pesticide exposure, we employed a well-documented cohort of 757 PD patients enrolled in the Parkinson’s, Environment, and Genes (PEG) study in a case only-approach to investigate gene-environment interactions. This patient cohort has been followed for decades with a detailed recording of environmental and clinical data, including exposure to agricultural pesticides^13^. As agricultural pesticides are generally not applied individually but in combination, we focus on individuals who were exposed to a cluster of pesticides that are typically co-applied to cotton and related crops within the same growing season. This pesticide cluster (which we termed “cotton cluster”) includes organophosphorus, organoarsenic, and n-methylcarbamate chemical classes and was selected for several reasons, including strong epidemiologic association with PD in our PEG case control study coupled with experimental evidence indicating exposure to the cluster, and notably the pesticides trifluralin and tribufos, results in neurotoxicity to iPSC patient-derived dopaminergic neurons^14^. Furthermore, in our study population, exposure to the cotton cluster is not correlated with other pesticide clusters created using a hierarchical clustering method, thus providing an exposure measure that is not confounded by other pesticide exposures (Supplementary Fig. 1).

**Fig. 1:**
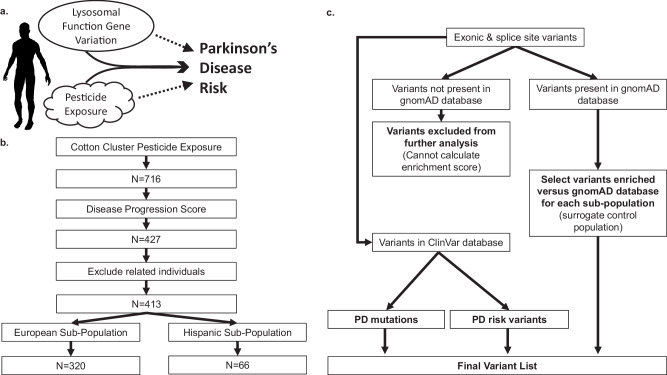
Study design. **a** Hypothesis. Parkinson’s disease risk is known to be influenced by variants in genes associated with lysosomal function as well as environmental influences such as pesticide exposure (dashed lines). In this study, we investigated the relationship between both lysosomal gene variation and pesticide exposure in a PD cohort. **b** Patient selection. To assess degree of pesticide exposure as a variable in combination with disease progression, the cohort was sub-divided as shown. **c** Variant selection and analysis workflow. HGMD: Human Gene Mutation Database.

The goal of this study was to assess whether exonic variants in genes known to be involved in lysosomal function are enriched in PD patients with this cotton cluster pesticide exposure (Fig. 1a, Table 1). To identify those variants with the greatest potential impact, we examined disease progression and focused on genetic variants in patients with the most progressive disease. This may ultimately suggest that alterations in lysosomal function encoded by these variants may modify the known risk associated with pesticide exposure in the development of PD.

**Table 1:** Cohort demographics and clinical characteristics at baseline examination

*N* (%) or Mean ± SD	All Patients (*N* = 757)	Disease Progression: All Patients (*N* = 386)	Progression: European Ancestry (*N* = 320)	Progression: Hispanic Ancestry (*N* = 66)
Age at diagnosis, years	67.7 ± 10.6	66.2 ± 10.0	67.0 ± 9.6	62.4 ± 10.9
Sex, Male	468 (61.8%)	241 (62.4%)	192 (60.0%)	49 (74.2%)
Ethnicity, Hispanic	138 (18.2%)	66 (17.1%)	0 (0%)	66 (100%)
Race, White	695 (91.8%)	360 (93.3%)	320 (100%)	40 (60.6%)
PD duration at diagnosis, years	2.9 ± 2.5	2.8 ± 2.4	2.6 ± 2.4	3.5 ± 2.6
Levodopa Use (yes)	530 (70.0%)	269 (69.7%)	226 (70.6%)	43 (65.2%)
LED, mg/day	311 ± 289	290 ± 277	296 ± 284	269 ± 239
UPDRS-III	21.5 ± 11.4	20.1 ± 10.6	18.9 ± 9.9	25.8 ± 12.1
Rigidity	3.5 ± 2.5	3.4 ± 2.5	3.2 ± 2.3	4.3 ± 3.0
Bradykinesia	1.1 ± 0.9	1.0 ± 0.8	0.9 ± 0.8	1.5 ± 0.9
Tremor	3.1 ± 2.6	3.1 ± 2.5	3.0 ± 2.4	3.3 ± 2.7
PIGD	1.7 ± 1.6	1.4 ± 1.3	1.3 ± 1.3	1.8 ± 1.5
HY (mean)	2.1 ± 0.8	2.0 ± 0.7	1.9 ± 0.7	2.2 ± 0.7

*HY* Hoehn and Yahr scale, *LED* Levodopa, *PD* Parkinson’s disease, *PIGD* Postural Instability and Gait Difficulties, *UPDRS-III* Movement Disorder Society-Unified Parkinson’s Disease Rating Scale version III.

## Results

### Gene enrichment analysis to pesticide exposure

A cohort of 757 PD patients from the Parkinson’s, Environment, and Genes (PEG) study was utilized for this study. Overall, 62% of the cohort was male (468/757) with an average age at PD diagnosis of 67.7 years (standard deviation 10.6 years, range 23–89 years), and primarily of White European/non-Hispanic (75%, 571/757) descent (Table 1). We sought to investigate the etiology of Parkinson’s disease in this cohort using a gene-environment analysis in a case-only study approach to examine rare variation in 85 genes (Supplementary Table 1) associated with Parkinson’s disease risk and/or lysosomal function in the setting of exposure to pesticides typically applied to cotton (cotton cluster, Supplementary Fig. 1). This cohort was previously screened for known mutations and risk alleles in genes associated with Parkinson’s disease (Supplementary Text, Supplementary Tables 2 and 3).

Due to the study location in agricultural regions of Central California, this patient population has been subjected to repeated ambient pesticide exposure over an extended period of time. We therefore hypothesized that the development of Parkinson’s disease for many in this patient cohort is due to a combination of genetics and these environmental influences, with differing effect sizes depending on the specific genetic change and level of exposure in each individual (Fig. 1a). We sought to specifically investigate the genetic component that may modify PD risk by analyzing the cohort using a custom amplicon panel comprising 85 genes (Supplementary Table 1) associated with Parkinson’s disease risk and/or lysosomal function. Genes were categorized into 5 groups representing known PD risk genes (Group 1) and genes involved in lysosomal function selected based on specified criteria related to known function, interaction with established PD risk genes, expression in the substantia nigra, or previous observed association with PD risk (Groups 2–5, Supplementary Table 1). We calculated enrichment of identified variants in comparison to their respective self-identified population (White, European/non-Hispanic or Hispanic) in the gnomAD database. We hypothesized that greater magnitude of pesticide exposure is associated with greater PD risk, and consequently genetic variants compounding this PD risk would be found in individuals with the highest exposure and most progressive disease. Therefore, to identify genetic variants most closely associated with high pesticide exposure status and aggressive PD, subjects were next classified according to exposure level to the cotton pesticide cluster and by severity of disease progression (Fig. 1b). To maximize the chances of identifying pesticide exposure-associated variants modifying gene product function, we focused exclusively on exonic gene variants (Fig. 1c).

Variants identified in the 85 genes selected for this study (Supplementary Table 1) were filtered for gene enrichment analysis based on the following workflow to identify genetic variants enriched for pesticide and disease status (Fig. 1c). Because our local non-PD control population had not been sequenced on this platform, we compared variant frequency to the most racially equivalent White European/non-Hispanic group in the gnomAD database, the non-Finnish European population, and the most racially equivalent Hispanic group, the Latino/Admixed American population, as relative control populations (Fig. 1c). A total of 224 enriched exonic variants were identified (Supplementary Table 4) and further analyzed. 7 of these variants (3%) were enriched in both the European and Hispanic sub-populations. We excluded 54 variants that were not found within the gnomAD database, 16 (30%) of which were observed in both sub-populations, as we could not calculate an enrichment score for them (Fig. 1c, Supplementary Table 4).

**Fig. 2:**
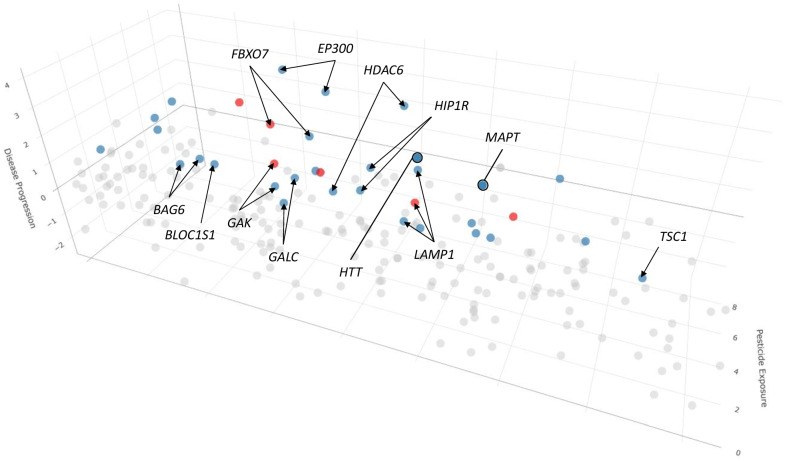
Genetic variants enriched in the PEG study patient cohort prioritized by pesticide exposure and disease progression. All enriched genetic exonic variants are shown and arranged by measures of exposure to the cotton cluster pesticides and disease progression. For pesticide exposure, scores represent scaled values derived from weighting individual pesticides in the cotton cluster by toxicity and exposure occurrence where a score of 1 represents the reference level for the cohort while higher numbers indicate increased toxicity and/or exposure. For disease progression, scores are scaled such that a score of 1 represents baseline symptom severity and rate of progression in the cohort while higher numbers represent faster progression and/or more severe symptoms. Variants with both an average weighted sum of 1 or greater for cotton cluster pesticide exposure and an average disease progression score of 1 or greater are highlighted. Blue points were found to be enriched in subjects of European/non-Hispanic descent while red points represent variants found to be enriched in patients of Hispanic descent. For *LAMP1*, the same variant is shown in both blue and red as it is enriched in both sub-populations. Genes with multiple variants identified and/or genes with single variants identified in multiple individuals in the patient cohort are highlighted. Arrows indicate single variants while circles represent two variants.

To identify genetic variants most associated with disease in the context of pesticide exposure, for each variant we examined the magnitude of pesticide exposure and the severity of disease progression across the cohort (Fig. 2, Table 2, Supplementary Fig. 2, Supplementary Table 5). Because not all subjects had sufficient clinical information available for this analysis, the study population was consequently reduced to 386 individuals (Fig. 1b, Table 1). We focused on variants with an average weighted sum of 1 or greater for cotton cluster pesticide exposure and an average disease progression score of 1 or greater, resulting in a prioritized total of 36 variants representing 26 genes (Fig. 2, Table 2, Supplementary Fig. 2, Supplementary Table 5). To rank these variants, we generated a disease severity x pesticide exposure score by multiplying the normalized average disease progression value by the normalized average mean weighted sum of pesticide exposure (Fig. 2, Table 2, Supplementary Fig. 2, Supplementary Table 5).

**Table 2:** Genes and variants enriched in the PEG cohort ranked by disease severity x pesticide exposure score

Gene Symbol	Gene Name	Gene Function	Variants Enriched in PEG Cohort	Variant Population *P* value	Average Disease x Pesticide Score
*EP300*	E1A Binding Protein P300	Regulates transcription via chromatin remodeling and cell proliferation and differentiation	NP_001420.2:p.Asp2412Glu NP_001420.2:p.Asn251Ser	8.40E-21 2.98E-02	0.497
*HDAC6*	Histone Deacetylase 6	Alters chromosome structure and affects transcription factor access to DNA	NP_006035.2:p.Gly844Arg NP_006035.2:p.Leu508Ser	7.30E-22 5.34E-05	0.259
*HTT*	Huntingtin	Regulate transcription and is required for normal development	NP_002102.4:p.Asp758Asn NP_002102.4:p.Arg3109His	2.30E-40 1.38E-17	0.235
*CTSD*	Cathepsin D	Protein turnover and in the proteolytic activation of hormones and growth factors	NP_001900.1:p.Ile198Val	5.40E-13^a^	0.230
*ACP2*	Acid Phosphatase 2, Lysosomal	Hydrolyze orthophosphoric monoesters to alcohol and phosphate	NP_001601.1:p.Thr358Ile	1.26E-06	0.229
*PRKN (PARK2)*	Parkin RBR E3 Ubiquitin Protein Ligase	Multiprotein E3 ubiquitin ligase complex mediate substrate proteins for proteasomal degradation	NP_004553.2:p.Pro37Leu	4.59E-02	0.229
*FBXO7*	F-Box Protein 7	SCFs (SKP1-cullin-F-box) ubiquitin protein ligase complex for phosphorylation-dependent ubiquitination	NP_036311.3:p.Asp516His NP_036311.3:p.Val485Ile	1.76E-04^a^ 1.71E-02	0.207
*APP*	Amyloid Beta Precursor Protein	Promote transcriptional activation	NP_000475.1:p.Glu665Lys	7.18E-21	0.166
*MAPT*	Microtubule Associated Protein Tau	Maintain stability of microtubules in axons	NP_001116538.2:p.Thr30Ile NP_001116538.2:p.Lys293Arg	9.77E-04 2.29E-03	0.160
*ATG4C*	Autophagy Related 4C Cysteine Peptidase	Autophagy	NP_116241.2:p.Thr452Met	7.90E-10	0.130
*HIP1R*	Huntingtin Interacting Protein 1 Related	Phosphatidylinositol phosphate binding activity	NP_003950.1:p.Arg613Gln NP_003950.1:p.Arg586Trp	4.44E-23 2.32E-07	0.124
*GNPTAB*	N-Acetylglucosamine-1-Phosphate Transferase Subunits Alpha And Beta	Catalyzes synthesis of mannose 6-phosphate recognition markers for trafficking of lysosomal enzymes	NP_077288.2:p.Met1175Ile	2.78E-14	0.111
*GBA1*	Glucosylceramidase Beta 1	Glycolipid metabolism	NP_001005742.1:p.Arg78Cys	8.43E-31^a^	0.110
*LAMP1*	Lysosomal Associated Membrane Protein 1	Provides selectins with carbohydrate ligands	NP_005552.3:p.Thr189Ser NP_005552.3:p.Ile309Thr	8.29E-06, 4.12E-02^a^ 7.91E-03	0.105
*SMPD1*	Sphingomyelin Phosphodiesterase 1	Converts sphingomyelin to ceramide	NP_001007594.2:p.Arg497Leu	1.96E-07	0.088
*PINK1*	PTEN Induced Kinase 1	Protect cells from stress-induced mitochondrial dysfunction	NP_115785.1:p.Ala383Thr	1.10E-04^a^	0.076
*BLOC1S1*	Biogenesis Of Lysosomal Organelles Complex 1 Subunit 1	Biogenesis of specialized organelles of the endosomal-lysosomal system	NP_001478.2:p.Asp68Asn	4.36E-02	0.074
*MCCC1*	Methylcrotonyl-CoA Carboxylase Subunit 1	Catalyzes the carboxylation of 3-methylcrotonyl-CoA to form 3-methylglutaconyl-CoA	NP_064551.3:p.Glu717Lys	1.89E-05	0.073
*BAG6*	BAG Cochaperone 6	Control of apoptosis and acetylation of p53 in response to DNA damage	NP_542434.1:p.Pro429Leu NP_542434.1:p.Glu293Lys	7.72E-88 8.78E-04	0.063
*GAK*	Cyclin G Associated Kinase	Cyclin-dependent protein kinase	NP_005246.2:p.Ile413Val NP_005246.2:p.Arg654Gln	4.40E-03^a^ 3.44E-02	0.062
*GALC*	Galactosylceramidase	Hydrolyzes galactose ester bonds of galactosylceramide, galactosylsphingosine, lactosylceramide, and monogalactosyldiglyceride	NP_000144.2:p.Ile144Leufs*27 NP_000144.2:p.Thr112Ala	6.21E-09 6.04E-03	0.056
*NPC1*	NPC Intracellular Cholesterol Transporter 1	Mediates intracellular cholesterol trafficking	NP_000262.2:p.Ile787Val	2.63E-02	0.055
*ACMSD*	Aminocarboxymuconate Semialdehyde Decarboxylase	NAD synthesis	NP_612199.2:p.Lys134Glu	3.36E-02	0.049
*MCOLN1*	Mucolipin TRP Cation Channel 1	Regulation of lysosomal exocytosis	NP_065394.1:p.Ala164Thr	2.17E-03	0.047
*TSC1*	TSC Complex Subunit 1	Rapamycin complex 1 (mTORC1) signaling	NP_001155899.1:p.Gly984Ser	7.49E-04	0.046
*LAMP3*	Lysosomal Associated Membrane Protein 3	Differentiate dendritic cells	NP_055213.2:p.Ala267Thr	5.36E-10	0.036

^a^*P* value of enrichment from Latino/Admixed American population. All other *p* values derived from the European (non-Finnish) population. For genes with multiple variants and/or genes with variants present in both European and Hispanic populations, Disease Severity x Pesticide Exposure scores were averaged to obtain a composite score.

Of the resulting prioritized 36 enriched variants, the majority were found to be related to lysosomal function (Groups 2–5, 26/36 variants, 72%). Surprisingly only a small number of variants represented Group 2 (4/36 variants,11%), the most stringently defined lysosomal function category, or Group 5, lysosomal function genes previously associated with PD risk (3/36 variants, 8%). Instead, the majority of variants represented genes from Group 3 (10/36 variants, 28%) and Group 4 (9/36 variants, 25%), defined by protein-protein interactions with known PD genes or high expression in the substantia nigra, respectively (Supplementary Table 1).

The majority of observed prioritized enriched variants were seen only in a single individual within the cohort (31/36, 86%) (Supplementary Table 5). We therefore focused on the variants observed in multiple individuals and the genes with multiple variants observed in the cohort as these were the most likely to be associated with increased risk due to pesticide exposure. Ten genes (10/26, 38%) were represented by multiple variants (Fig. 2, Table 2). The *BAG6, EP300, FBXO7, GAK, GALC, HDAC6, HIP1R, HTT, LAMP1* and *MAPT* genes had 2 variants each (2/36, 6%). Different *FBXO7* and *GAK* variants were enriched in individuals from the European and Hispanic sub-populations respectively (Supplementary Table 5). The remaining 16 genes all had one variant each (Supplementary Table 5). Variants in 5 genes (5/26, 19%) (*BLOC1S1, FBXO7, GALC, LAMP1, and TSC1*) were observed in multiple individuals (Supplementary Table 5). Each gene had a variant that was enriched in multiple individuals in the European sub-population, however *LAMP1* also had a second variant that was enriched in single individuals from both the European and Hispanic sub-populations (Supplementary Table 5). Three genes (3/26, 12%) (*FBXO7, GALC, LAMP1)* had both multiple variants observed and multiple individuals with the same variant (Supplementary Table 5).

To assess the contribution of each gene and variant to PD in the setting of pesticide exposure, variants were ranked by a calculated disease severity x pesticide exposure score (Fig. 2, Table 2, Supplementary Figure 2, Supplementary Table 5). For genes with multiple variants, *EP300* variants represented the top 2 highest scores overall. Two *HTT* variants and two *FBXO7* variants were also among the top 10 highest scoring. *MAPT* and *HIP1R* both had 2 variants among the top 20 highest scores. *ACP2, CTSD, HDAC6*, and *PRKN* each had a single variant represented in the 10 highest scores. One variant in *GBA1* was found in the top 20 highest scores. Of the genes with variants seen in multiple individuals, *FBXO7* and *LAMP1* had two and one variants within the top 10 and top 20, respectively, for disease severity x pesticide exposure scores (Supplementary Table 5). The *LAMP1* variant was also observed in both the European and Hispanic subpopulations.

As an initial measure of functional impact, we assessed the tolerance of the identified genes for variation using the metrics defined in the gnomAD database. Four genes with multiple enriched variants identified (4/26, 15%) (*BAG6, EP300, HDAC6, HTT*) were restricted for both loss-of-function and missense variants (probability of being loss-of-function intolerant, pLI > 0.9 and missense Z-score > 2). One gene, *LAMP1* (1/26, 4%), was restricted only for loss-of-function variants. As a measure of the strength of the genetic effects of the individual variants, we examined the potential functional effect of the identified variants on their respective proteins using the CADD (Combined Annotation Dependent Depletion) model which evaluates the impact of all possible substitutions in the human reference genome^15^. Using this method, a scaled score of 10 or more represents the 10% most deleterious substitutions in the human genome while a score of 20 or more reflects the top 1% most deleterious substitutions^15^. Of the 36 prioritized enriched exonic variants, 31 (31/36, 86%) were highly deleterious with either CADD scores between 10 and 20 (11/36 variants, 31%) or greater than 20 (20/36, 56%) (Supplementary Table 5). For genes with multiple observed variants, *EP300* had one variant scoring above 20, which also achieved the highest value based on the calculated disease severity x pesticide exposure score (Supplementary Table 5). The two *HTT* variants that ranked among the top 10 highest scoring variants based on the disease severity x pesticide exposure score both had CADD scores above 20 as well, including one with the highest observed CADD score of all variants (Supplementary Table 5). Of the variants observed in multiple individuals, four variants (4/6, 67%) had CADD scores greater than 10 with the other 2 (2/6, 33%) having scores greater than 20 including the most commonly observed variant in *GALC* (seen in 7 subjects, CADD 24.4) (Supplementary Table 5).

As a means to assess whether any of these variants might contribute to PD risk independently of pesticide exposure, we asked whether any of the identified prioritized variants were enriched in a large publicly-available independent cohort of 496 PD patients and 192 healthy controls from the Parkinson’s Progression Marker Initiative (PPMI)^16^, not known to have an excessive level of pesticide exposure. We were unable to evaluate variants that were not present in the PPMI cohort (22/36 variants, 61%). Of the remaining variants (14/36 variants, 39%), 3 were enriched in the PPMI PD cohort (3/36 variants, 8%) representing 2 variants seen in multiple PEG subjects in the *GALC* (7 subjects) and *TSC1* (5 subjects) genes, as well as the highest ranked *FBXO7* variant based on the calculated disease severity x pesticide exposure score (Supplementary Table 5). The remaining variants (11/36, 31%) were not enriched in PPMI cohort (Supplementary Table 5).

## Discussion

In this study, we sought to identify genetic contributions to the risk of developing Parkinson’s disease in the setting of chronic high pesticide exposure. To do so, we evaluated 757 patients with Parkinson’s disease, resulting in 386 subjects with detailed pesticide exposure levels and disease progression scores which we subdivided by race/ethnicity for genetic analysis. We subsequently identified 36 genetic exonic variants in 26 genes enriched in PD patients exposed to high levels of pesticides typically used on cotton and prioritized based on severity of disease progression. Although we also examined 14 known genes associated with PD risk, the majority of these identified variants (26/36, 72%) were found in genes associated with lysosomal function. Association between pesticide exposure and disease progression is supported by the observation that 12 genes (12/26, 46%) had either multiple variants or variants present in multiple individuals in the PEG cohort (Fig. 2, Table 2). The impact of these variants on function is likely also important as 31 variants (31/36, 86%) were deemed deleterious with CADD scores between 10 and 20 (11/36 variants, 31%) or highly deleterious with scores greater than 20 (20/36, 56%) (Supplementary Table 5).

Multiple lines of evidence implicate impaired autophagy-lysosomal pathways in Parkinson’s disease^17,18^. Lysosomes, which are intracellular organelles that contain hydrolytic enzymes, are crucial for degrading proteins, including aggregated alpha-synuclein, organelles, such as damaged mitochondria, and other intracellular components through autophagy^17,18^. Animal models and postmortem brain samples from patients have demonstrated lower levels of lysosomes and lysosomal-associated proteins (e.g., LAMP1) along with an accumulation of autophagosomes in PD^19^. Large genome-wide genetic studies along with familial studies have linked variants in autophagy and lysosomal related genes, including *GBA1*, while other PD-related gene products (e.g., PRKN, PINK2) have now been shown to have roles in autophagy-lysosomal pathways ^18^.

The specific role of the identified variants in modulating PD risk and disease progression in the setting of pesticide exposure is unknown but it is reasonable to assume that it may involve impairment of protein function and disruption of specific lysosomal pathways. Supporting this, the gene with the highest pesticide exposure- and disease progression-associated variants identified in this study was *EP300*. The *EP300* gene had 2 variants in the prioritized variant group (2/36, 6%) including the two highest scoring variants based on pesticide exposure and severity of disease progression overall (Table 2, Supplementary Table 5). In assessing functional impact, one of these *EP300* variants (the highest scoring overall by pesticide exposure and disease progression) had a CADD score above 20, in the most deleterious range, suggesting this variant negatively impacts protein function (Supplementary Table 5). *EP300* encodes a histone acetyltransferase involved in cell proliferation and differentiation and plays an important role in autophagy ^20^.

Research investigating pesticides and autophagy is still accumulating, however, several pesticides have been shown to influence the process^21^. For example, the cotton cluster contains sodium cacodylate, an organic arsenic compound used as an herbicide. Arsenic-containing pesticides have previously been related to PD^14,22^. Furthermore, studies have demonstrated arsenic promotes protein aggregation^23^, can induce the accumulation of alpha-synuclein^24^, and inhibits autophagic flux^25^. We have also previously shown using the Comparative Toxicogenomics Database that the chemical-gene network linked to sodium cacodylate is enriched for autophagy-related gene sets determined through gene ontology^26^. Beyond arsenic-containing pesticides, several of the cotton cluster pesticides have been linked to mitochondrial dysfunction and elevated reactive oxygen species, which can induce autophagy. Trifluralin, for instance, was found to reduce the spare capacity of mitochondria in PD-patient-derived dopaminergic neurons^14^. Prometryn exposure led to mitochondrial and proteasome dysfunction^27^, while phorate induced oxidative stress and DNA damage ^28^.

Therefore, as some pesticides may alter autophagy, a 2-hit model to the lysosomal system where exposure would add to the genetic variant’s impact is plausible^29–33^. A similar gene-environment interaction has been seen with aldehyde dehydrogenase gene (ALDH) variants and ALDH-inhibiting pesticides^34^. Dysfunction of autophagy has also been implicated in other neurodegenerative diseases such as Alzheimer’s disease^35^, and the added toxicity from pesticide exposure coupled with a potent genetic variant affecting autophagy might very well cause a Parkinsonian disorder. Supporting this, other genes with multiple variants scoring highly on disease severity and pesticide exposure observed in this study (Table 2, Supplementary Table 5) also show links to autophagy, including *HTT*^36^, *LAMP1*^37^, and *MAPT*^38^, or mitophagy, such as *FBXO7*^39^. Another gene with multiple observed enriched variants, *BAG6*, modifies autophagy via modulation of EP300 ^40^.

Only three variants (3/36, 8%) were observed to be enriched in an independent PD cohort from the Parkinson’s Progression Marker Initiative (PPMI), likely without pesticide exposure comparable to the PEG cohort, suggesting that the majority of variants we identified (11/14 variants, 79% of variants seen in both PEG and PPMI cohorts) contribute most to PD risk when individuals are pesticide exposed (Supplementary Table 5). It is also notable that 2 of the PEG variants also enriched in the PPMI cohort (in the *GALC* and *TSC1* genes) were observed in multiple PEG subjects (Supplementary Table 5). This may reflect that some variants exhibit a baseline PD risk that can be augmented by pesticide exposure. This is further supported by the observation of 10 prioritized variants (10/36, 28%) being observed in genes with a known association to PD risk. *GAK*, *HIP1R*, and *MAPT* each had 2 variants apiece (2/10, 20%). *GBA1*, the most common PD risk gene, had one variant (1/10, 10%), as did *ACMSD, LAMP3*, and *MCCC1*. Of these 10 variants, 9 (9/10, 90%) had CADD scores above 10 with 6 (6/10, 60%) greater than 20, suggesting the majority of these variants may impact protein function.

The cluster of pesticides investigated in this study were chosen from an untargeted analysis of all agricultural pesticides applied in our study region^14^. In total 68 pesticides were implicated with PD in our study. However, combinations of pesticides are often seasonally applied to the same fields, and we identified multiple clusters of strongly correlated exposures among the 68 pesticides, one of which was the cotton cluster. We selected this cluster for further genetic analysis due to the strong epidemiologic association with PD coupled with experimental evidence indicating exposure to these chemicals in combination results in toxicity to dopaminergic neurons^14^. Furthermore, several of the individual pesticides in the cluster have been previously linked to PD. Trifluralin and phorate, for instance, were associated with PD in the Agricultural Health Study^41^, while aldicarb was linked to PD in a Dutch epidemiologic study^42^. Still other pesticides have been linked to autophagy and PD, and would be of interest for future analysis, including chlorpyrifos, paraquat, and malathion ^21^.

There are several important limitations to this study. Related to study design, the size of the cohort, further reduced to generate subgroups of pesticide exposure and disease progression, limits a more comprehensive analysis of variant frequency. Correspondingly, because of the size differences between the sub-populations, most of the prioritized enriched variants were derived from subjects of European descent (31/36 variants, 86%) with one variant (1/36, 3%, in the *LAMP1* gene) enriched in both sub-populations. The lack of a specific control population that more closely mimics the racial and ethnic composition of the cohort sequenced on the same platform could have influenced the magnitude of the observed variant enrichment. The size limitations of both our cohort and the PPMI cohort may also have limited the observance of shared variation unrelated to pesticide exposure. Lastly, the limited number of genes tested and their method of selection could bias the results in favor of variants in the genes examined, whereas a genomic approach might yield more promising candidates. Given that PD populations with detailed environmental exposure, such as agricultural pesticides, are rare and the PEG study is unique in that aspect, these are important considerations for genomic studies in the development of future cohorts. Ideally, the implementation of genomic sequencing methods, with either whole exome or whole genome sequencing, to the design of future studies will allow the identification of PD risk-associated variation more broadly across the genome.

An additional limitation of this study is the inability to accurately assess the contribution of variants in genes which were not present in the gnomAD database as an enrichment score could not be calculated (Supplementary Table 4). It is possible that some of this variation may also contribute to PD risk in the setting of pesticide exposure. Additionally, although we can observe enrichment of specific gene variants in subjects with high pesticide exposure, we cannot determine whether the primary contribution of such variants is related to interactions with other genetic factors or with the pesticide cluster itself. We did note that 14 individuals in the cohort carried at least 2 enriched variants so we assessed for a potential additive effect by comparing the average disease severity x pesticide exposure scores of these individuals with those individuals carrying only a single enriched variant. We found no statistical difference (*p* = 0.43, data not shown) between the groups and therefore considered each variant as acting independently for the purpose of this analysis, however we cannot rule out that such interactions may occur. Future study of these genetic variants in combination with pesticide exposure using model systems may further address this question.

In conclusion, this study supports a relationship between genes associated with lysosomal function and environmental exposure to pesticides in the development and progression of PD. Further study of these genes and variants in conjunction with environmental exposures could aid the identification of novel mechanisms for PD through gene-environment interactions and eventually lead to better methods of prevention of PD in individuals exposed to pesticides or improved disease treatments.

## Methods

### Patient recruitment

This work involved 757 PD patients of primarily European ancestry from the Parkinson’s, Environment, and Genes (PEG) study (Table 1). Participants were enrolled in two waves (PEG1 from 2001 to 2007 and PEG2 from 2010 to 2014)^13^. All study participants were confirmed to have probable idiopathic PD and were seen at least once at baseline by a UCLA movement disorder specialist and most were seen repeatedly over follow-up. Written informed consent was obtained from all participants. All methods in this study were approved by the UCLA Institutional Review Board.

### Pesticide exposure assessment

The study population resides in central California, a region known for its intense agriculture. We estimated long-term ambient pesticide exposure to specific pesticide active ingredients due to living and working near agricultural pesticide applications using a geographic information systems (GIS)-based model and record-based pesticide application data recorded in the Pesticide Use Report (PUR) database^14,43^.

Since 1974, California requires by law that commercial agricultural pesticide applications be reported into the PUR database. This database records the method and date of applications, location, poundage applied, type of crop, and acreage a pesticide was applied on. We combined the PUR data with land-use maps for crop cover to determine pesticide applications at specific agricultural sites. Study participants provided lifetime residential and workplace address information which allowed us to determine the pounds of each pesticide active ingredient applied per acre within a 500 m buffer of each address yearly since 1974, weighing the total poundage applied by the proportion of acreage treated (lbs/acre).

We were interested in long-term exposures as likely the most relevant for PD and therefore considered an exposure window starting in 1974 and ending 10 years prior to PD diagnosis, to account for a prodromal PD period. For each pesticide applied in the study region (*n* = 722) and at each location separately (residential and workplace exposures), we averaged the annual lbs/acre estimates to create one long-term summary exposure estimate for each pesticide. More detail on this has been published^14^. We have previously determined that out of all pesticides applied in the study area, 68 were most strongly associated with PD. We then created co-exposure clusters based on hierarchical correlation analysis using a cut-point of *R* > 0.45^14^. The cluster we selected here is based on ambient exposure to 10 pesticides commonly applied on cotton (termed “cotton cluster”, Supplementary Fig. 1). By design, exposure to a cluster is not correlated above the preset cut-point mentioned above with any other PD-associated pesticide exposure.

To create a summary exposure score for the cluster, we generated a weighted sum of the long-term yearly average pounds of pesticide applied per acre of all pesticides in the cluster. The weighting scheme was designed to account for the differences in toxicity per pound for different pesticides in the cluster. Each pesticide’s lbs/acre estimate (log transformed and scaled to the SD) was first weighted by the beta for its association with PD on the log odds scale determined from an untargeted PD-pesticide meta-analysis^14^, then these measures were summed. Ambient exposures at residential and workplace locations were included separately, with the highest exposure (i.e., estimated ambient exposure at both locations) generating the highest values, compared with exposure at only one location or at no location. The resulting summary cluster exposure score was scaled to the SD (Supplementary Fig. 2).

### Disease progression

PD disease progression was measured from clinical data derived from the PEG study cohort using a model predicting repeated measures of the Movement Disorder Society-Unified Parkinson’s Disease Rating Scale version III (MDS-UPDRS-III) across an initial visit and up to two follow-up visits^44^. Random effects to longitudinal measures of the MDS-UPDRS-III (Table 1) were adjusted for age at diagnosis, race/ethnicity, gender, PD duration at baseline, PD family history, school years, and study wave treated as fixed effects. The random effects included a random intercept with respect to individuals and a random slope with respect to visit to account for heterogeneous changes in PD scores across individuals that are not captured by the fixed effect of visit. Empirical Bayes estimates of the random slopes were computed as an initial correlate of PD progression followed by regression of the random slopes on the above covariates and the residuals were then used as the measurement of PD progression for further analysis (Supplementary Fig. 2).

### Gene selection strategy, custom amplicon design, and sequencing

To test the hypothesis that Parkinson’s disease risk is influenced by variants in genes associated with lysosomal function as well as environmental influences such as pesticide exposure, we initially pre-selected 85 genes associated with lysosomal function for this study (Supplementary Table 1) based on five sets of criteria described below. The five groups (Groups 1–5) were designed to prioritize genes with the highest likelihood for risk to Parkinson’s Disease. To outline the selection strategy, we first included all known PD risk associated genes compiled from the literature (Group 1) to determine if their known baseline risk would be influenced by pesticide exposure. Next, we included a list of genes involved in lysosomal function (Group 2) based on a strict keyword search. Next, we included a more permissive keyword search of genes involved in lysosomal function but required them to have known protein-protein interactions with at least one of four well-established PD risk genes (*GBA1, SNCA, MAPT*, or *LRRK2)*, reasoning that mutations in such genes might impact pathways involved in PD risk (Group 3). Group 4 was derived in similar fashion except that after a permissive keyword search of genes involved in lysosomal function, we selected the genes most highly expressed in the substantia nigra, reasoning that high expression in a key area of PD pathology could imply a relevant contribution to PD risk. Lastly, Group 5 consisted of additional lysosomal function genes that were reported in the literature as associated with PD risk during the course of this project. To briefly describe the process of gene selection, Group 1 (PD Risk Genes) utilized a literature review^2^ to identify 14 well-known PD risk genes. Group 2 (Lysosomal Function) used a strict database search for genes whose function included multiple keywords, specifically mitophagy, autophagy and lysosome/lysosomal function, resulting in 13 genes. Group 3 (Lysosomal Function Genes that are PD Interactors) used a permissive keyword search for autophagy and/or lysosome/lysosomal function and required the resulting genes to be protein−protein interactors of well-established PD risk genes (*GBA1, SNCA, MAPT*, or *LRRK2)*, resulting in 18 genes. Group 4 (Lysosomal Function Genes Highly Expressed in the Substantia Nigra) used a permissive keyword search for autophagy and/or lysosome/lysosomal function and selected genes ranked by highest expression level in the substantia nigra, resulting in 36 genes. Group 5 (Other Lysosomal Storage Disorder Genes) consisted of 4 additional lysosomal storage disorder (LSD) genes (*ASAH1, SLC17A5, GALC*, and *GNPTAB*) with reported contributions to PD risk following an additional literature review^11^. Keyword search and protein-protein interaction analysis were performed with the use of QIAGEN IPA (QIAGEN Inc., https://digitalinsights.qiagen.com/IPA)^45^. Gene expression data was obtained from the GTEx (Genotype-Tissue Expression) Portal^46^.

A custom amplicon was designed exclusively targeting the 85 genes listed above for sequencing following PCR amplification of the selected exonic regions (coding sequence only, not including untranslated regions) and uploaded to Illumina’s DesignStudio software (Illumina, San Diego, CA) for TruSeq Custom Amplicon probe design (Supplementary File 1). A minimum of 10 bp sequence around each exon was included to detect splice site mutations. DNA was extracted from whole blood collected from PEG study PD patients. The TruSeq Custom Amplicon library preparation kit (Illumina, San Diego, CA) was used according to the manufacturer’s protocol to amplify the target regions of interest. Libraries were dual indexed (with up to 96 samples pooled in a library) and sequenced on a HiSeq4000 flow cell (8 lanes, Illumina, San Diego, CA). Reads were paired-end and 150 bp long. The average number of reads per sample was 3,717,232 with a standard deviation of 1,054,374 reads.

### Bioinformatics analysis

Sequencing reads were aligned to the human genome version GRCh37 (hs37d5) using the Burrows-Wheeler Alignment Tool^47^, and the Broad Institute’s Genome Analysis Toolkit (GATK3) was used for indel realignment^48^. Small-variant calling was performed with Illumina’s Pisces suite for germline amplicon sequencing applications^49^, and SAMtools^50^ was used to sort and index intermediary Binary Alignment Map files. GATK was also used to select for variants that were in the target regions. VarSeq (Golden Helix, Inc., Bozeman, MT, www.goldenhelix.com) software was used to annotate the variants. ClinVar^51^ and/or HGMD (Human Gene Mutation Database)^52^ were used to classify variants as pathogenic or likely pathogenic PD mutations or PD risk variants. Variants were filtered for inclusion in further analysis based on the pass quality filter, having at least 10x coverage in 90% of the samples within each sub-population, and having a Phred score of 30 or higher. Variants were further annotated with CADD v1.6 (Combined Annotation Dependent Depletion) Phred scores through the CADD web application (https://cadd.gs.washington.edu/score, accessed August 2023) ^15^.

To identify genetic variation in PD patients that correlated with the magnitude of pesticide exposure, we focused on those patients with the highest exposures and the most progressive disease. To do so, we first selected individuals that had both a pesticide cluster weighted sum exposure score (*n* = 716, Fig. 1b) and a disease progression score (*n* = 427, Fig. 1b). To rule out enrichment due to familial relationships, identity-by-descent (IBD) analysis was performed using genotype data (470,569 SNPs) generated from Illumina’s Global Screening Array using PLINK^53^ and samples with an IBD score >0.05 were excluded (*n* = 14, Fig. 1b). For the purpose of calculating gene variant enrichment, we divided this group into two sub-populations based on self-reported racial and ethnicity information (White, European/non-Hispanic, *n* = 320 and Hispanic subjects of admixed race, *n* = 66), resulting in a total of 386 subjects for genetic analysis (Fig. 1b). Self-reported ancestry was utilized for consistency as not all subjects had genotype data, but for those individuals where genotype was available (93%), a concordance of 99% was observed. We then utilized either the gnomAD non-Finnish European population or the Latino/Admixed American population, respectively, (Genome Aggregation Database v2.1.1, https://gnomad.broadinstitute.org/)^54^ as a control population to compare variant frequency in our cohort. Gene enrichment analysis was then performed by first filtering for enrichment against the variant frequency in the corresponding gnomAD population. No variant frequency threshold was employed, allowing inclusion of both common and rare variation, however variants that were absent in the gnomAD database were not further analyzed as an enrichment score could not be calculated (Fig. 1c). Additionally, variants in the *HTT* gene were excluded if they were in low complexity regions and variants in the *HLA-DRB5* gene were excluded if they had a low quality warning or were in phase with other variants that would alter population frequency for enrichment calculation. For variants where an enrichment score could be calculated, only variation mapping to exonic or splice-site regions in the gene’s canonical transcript were included in further analysis to maximize evaluation of variants with potential functional impact. Variants were prioritized using an average weighted sum of 1 for cotton cluster pesticide exposure and an average disease progression score of 1. To rank variants, we generated a disease severity x pesticide exposure score where the normalized average disease progression value was multiplied by the normalized average mean weighted sum of pesticide exposure (Supplementary Fig. 2).

To independently assess the relationship of the identified variants to PD independent of pesticide exposure, variants were compared to the whole genome sequencing data from 496 PD patient and 192 healthy controls (HC) from the Parkinson’s Progression Marker Initiative (PPMI) (https://www.ppmi-info.org/access-data-specimens/download-data, accessed January 2023)^16^. Gene enrichment analysis was performed comparing the variants identified in the PEG study cohort to the PPMI PD and PPMI HC cohorts for enrichment.

### Statistical analysis

Two-proportion testing was used to determine if identified variants were enriched compared to the gnomAD Non-Finnish European or Latino Admixed American Populations. Two-proportion testing was also used to compare variant enrichment between the PPMI PD and HC cohorts.

### Reporting summary

Further information on research design is available in the Nature Research Reporting Summary linked to this article.

### Supplementary information

Supplementary Material

Supplementary Tables - EXCEL

Reporting Summary

## Data Availability

Sequencing data is available at the Sequence Read Archive at the National Center for Biotechnology Information under the accession number PRJNA1089195.

## Code availability

No software was used for data collection. The following software was used for data analysis: QIAGEN IPA v01.07, Illumina’s DesignStudio, BWA-MEM v.0.7.5a-r405, GATK3 v.3.8-0-ge9d806836, Illumina’s Pisces v.5.1.6, VarSeq v2.2.0, CADD v1.6, and PLINK v1.9, R v.4.0.4.
