# Supplementary material for: Lysosomal genes contribute to Parkinson’s disease near agriculture with high intensity pesticide use
Source: NPJ Parkinsons Dis. 2024 Apr 25;10:87. doi: 10.1038/s41531-024-00703-4 (PMC11045791; doi:10.1038/s41531-024-00703-4)
Supplement: Supplementary file 1 — Supplementary Material [file 41531_2024_703_MOESM1_ESM.pdf]

Supplementary Text:

Assessment for Known Mutations and Risk Alleles Associated with Parkinson's Disease:

We evaluated the cohort for well-established genetic causes of Parkinson's disease and previously reported risk-associated alleles. Variant analysis identified twelve previously reported PD risk variants in the genes *GBA, LRRK2,* and *MAPT* of which 10 were missense and 2 were synonymous (Supplementary Table S2).[1-5] Variant analysis also identified five known pathogenic variants in the *LRRK2, PRKN,* and *PINK1* genes that were previously reported in the ClinVar database (Supplementary Table S3).[1] These variants were diagnostic for autosomal dominant Parkinson's disease 8 (*LRRK2,* PARK8) in 4 individuals and for autosomal recessive juvenile Parkinson's disease 2 (*PRKN,* PARK2/PDJ) in 2 homozygous individuals. In addition, 4 heterozygous carriers of a pathogenic *PRKN* variant and 1 heterozygous carrier for a pathogenic variant in *PINK1,* associated with autosomal recessive Parkinson's disease 6 PARK6), were also identified.[1] None of the subjects had known family histories of disease suggestive of a Mendelian disorder.

As noted above, a frameshift variant in the *PRKN* gene was found to be homozygous in two unrelated patients in the cohort who presented with sporadic young-onset Parkinson disease. Biallelic mutations in the *PRKN* gene are known to cause Parkinson's disease, juvenile, type 2 (PARK2 or PDJ, MIM# 600116).[6] The p.Asn52Metfs*29 (chr6:g.162864358delT, hg19) mutation has been previously reported, and is predicted to result in premature termination of the protein.[7-12] This mutation is

observed in the gnomAD database with a MAF (minor allele frequency) of 0.001 in the Latino/Admixed American population and MAF of 0.0002 in the European (non-Finnish) population.[13] Both patients were male, of Latino descent, and diagnosed with Parkinson's disease prior to the age of 40 years.

It is relevant that genetic forms of PD were observed in this cohort. Although subjects with atypical features (e.g., early cognitive involvement or dysautonomia) were excluded from PEG, phenotypes for Mendelian forms may sufficiently overlap with sporadic PD to have been included in this cohort. Interestingly, we were able to genetically diagnose four patients with dominant PARK8 and two patients with recessive PARK2 in a cohort of PD patients initially thought to have developed disease sporadically. Young-onset PD has an estimated incidence rate at 3 per 100,000 people per year.[14] In our cohort, we were able to diagnose 3.7% (2/54) patients with an early age at diagnosis (age <51 years)[15] with a Mendelian disorder by examining the most common PD-associated genes. This reinforces the clinical practice that all patients with an early age of PD diagnosis should be considered for genetic testing.

References:

1. Landrum MJ, Lee JM, Benson M, Brown GR, Chao C, Chitipiralla S, Gu B, Hart J, Hoffman D, Jang W, Karapetyan K, Katz K, Liu C, Maddipatla Z, Malheiro A, McDaniel K, Ovetsky M, Riley G, Zhou G, Holmes JB, Kattman BL, Maglott DR. ClinVar: improving access to variant interpretations and supporting evidence. Nucleic acids research. 2018;46(D1):D1062-D7. doi: 10.1093/nar/gkx1153.

2. Davis AA, Andruska KM, Benitez BA, Racette BA, Perlmutter JS, Cruchaga C. Variants in GBA , SNCA , and MAPT influence Parkinson disease risk, age at onset, and progression. Neurobiology of Aging. 2016;37:209.e1-.e7. doi: 10.1016/j.neurobiolaging.2015.09.014.

3. Lesage S, Anheim M, Condroyer C, Pollak P, Durif F, Dupuits C, Viallet F, Lohmann E, Corvol J-C, Honoré A, Rivaud S, Vidailhet M, Dürr A, Brice A, Agid Y, Bonnet AM,

Borg M, Brice A, Broussolle E, Damier P, Destée A, Dürr A, Durif F, Lesage S, Lohmann E, Martinez M, Pollak P, Rascol O, Tison F, Tranchant C, Troiano A, Vérin M, Viallet F, Vidailhet M. Large-scale screening of the Gaucher's disease-related glucocerebrosidase gene in Europeans with Parkinson's disease. Human Molecular Genetics. 2011;20(1):202-10. doi: 10.1093/hmg/ddq454.

4. Simpson C, Vinikoor-Imler L, Nassan FL, Shirvan J, Lally C, Dam T, Maserejian N. Prevalence of ten LRRK2 variants in Parkinson's disease: A comprehensive review. Parkinsonism & Related Disorders. 2022;98:103-13. doi: 10.1016/j.parkreldis.2022.05.012.

5. Maple-Grodem J, Paul KC, Dalen I, Ngo KJ, Wong D, Macleod AD, Counsell CE, Backstrom D, Forsgren L, Tysnes OB, Kusters CDJ, Fogel BL, Bronstein JM, Ritz B, Alves G. Lack of Association Between GBA Mutations and Motor Complications in European and American Parkinson's Disease Cohorts. J Parkinsons Dis. 2021;11(4):1569-78. doi: 10.3233/JPD-212657. PubMed PMID: 34275908; PMCID: PMC8609705.

6. Online Mendelian Inheritance in Man, OMIM. [Internet]. McKusick-Nathans Institute of Genetic Medicine, Johns Hopkins University (Baltimore, MD). [accessed October 2023]. Available from: World Wide Web URL: http://omim.org/.

7. Abbas N, Lücking CB, Ricard S, Dürr A, Bonifati V, De Michele G, Bouley S, Vaughan JR, Gasser T, Marconi R, Broussolle E, Brefel-Courbon C, Harhangi BS, Oostra BA, Fabrizio E, Böhme GA, Pradier L, Wood NW, Filla A, Meco G, Denefle P, Agid Y, Brice A. A wide variety of mutations in the parkin gene are responsible for autosomal recessive parkinsonism in Europe. French Parkinson's Disease Genetics Study Group and the European Consortium on Genetic Susceptibility in Parkinson's Disease. Human molecular genetics. 1999;8(4):567-74. doi: 10.1093/hmg/8.4.567.

8. Guerrero Camacho JL, Monroy Jaramillo N, Yescas Gómez P, Rodríguez Violante M, Boll Woehrlen C, Alonso Vilatela ME, López López M. High frequency of Parkin exon rearrangements in Mexican-mestizo patients with early-onset Parkinson's disease. Movement disorders : official journal of the Movement Disorder Society. 2012;27(8):1047-51. doi: 10.1002/mds.25030.

9. Hoenicka J, Vidal L, Morales B, Ampuero I, Jiménez-Jiménez FJ, Berciano J, del Ser T, Jiménez A, Ruíz PG, de Yébenes JG. Molecular findings in familial Parkinson disease in Spain. Archives of neurology. 2002;59(6):966-70. doi: 10.1001/archneur.59.6.966.

10. Lücking CB, Dürr A, Bonifati V, Vaughan J, De Michele G, Gasser T, Harhangi BS, Meco G, Denèfle P, Wood NW, Agid Y, Brice A, French Parkinson's Disease Genetics Study G, European Consortium on Genetic Susceptibility in Parkinson's D. Association between early-onset Parkinson's disease and mutations in the parkin gene. The New England journal of medicine. 2000;342(21):1560-7. doi: 10.1056/NEJM200005253422103.

11. Marder KS, Tang MX, Mejia-Santana H, Rosado L, Louis ED, Comella CL, Colcher A, Siderowf AD, Jennings D, Nance MA, Bressman S, Scott WK, Tanner CM, Mickel SF, Andrews HF, Waters C, Fahn S, Ross BM, Cote LJ, Frucht S, Ford B, Alcalay RN, Rezak M, Novak K, Friedman JH, Pfeiffer RF, Marsh L, Hiner B, Neils GD, Verbitsky M, Kisselev S, Caccappolo E, Ottman R, Clark LN. Predictors of parkin mutations in early-onset Parkinson disease: the consortium on risk for early-onset Parkinson disease study. Archives of neurology. 2010;67(6):731-8. doi: 10.1001/archneurol.2010.95.

12. Muñoz E, Tolosa E, Pastor P, Martí MJ, Valldeoriola F, Campdelacreu J, Oliva R. Relative high frequency of the c.255delA parkin gene mutation in Spanish patients with autosomal recessive parkinsonism. Journal of neurology, neurosurgery, and psychiatry. 2002;73(5):582-4. doi: 10.1136/jnnp.73.5.582.

13. Karczewski KJ, Francioli LC, Tiao G, Cummings BB, Alföldi J, Wang Q, Collins RL, Laricchia KM, Ganna A, Birnbaum DP, Gauthier LD, Brand H, Solomonson M, Watts NA, Rhodes D, Singer-Berk M, England EM, Seaby EG, Kosmicki JA, Walters RK, Tashman K, Farjoun Y, Banks E, Poterba T, Wang A, Seed C, Whiffin N, Chong JX, Samocha KE, Pierce-Hoffman E, Zappala Z, O'Donnell-Luria AH, Minikel EV, Weisburd B, Lek M, Ware JS, Vittal C, Armean IM, Bergelson L, Cibulskis K, Connolly KM, Covarrubias M, Donnelly S, Ferriera S, Gabriel S, Gentry J, Gupta N, Jeandet T, Kaplan D, Llanwarne C, Munshi R, Novod S, Petrillo N, Roazen D, Ruano-Rubio V, Saltzman A, Schleicher M, Soto J, Tibbetts K, Tolonen C, Wade G, Talkowski ME, Aguilar Salinas CA, Ahmad T, Albert CM, Ardissino D, Atzmon G, Barnard J, Beaugerie L, Benjamin EJ, Boehnke M, Bonnycastle LL, Bottinger EP, Bowden DW, Bown MJ, Chambers JC, Chan JC, Chasman D, Cho J, Chung MK, Cohen B, Correa A, Dabelea D, Daly MJ, Darbar D, Duggirala R, Dupuis J, Ellinor PT, Elosua R, Erdmann J, Esko T, Färkkilä M, Florez J, Franke A, Getz G, Glaser B, Glatt SJ, Goldstein D, Gonzalez C, Groop L, Haiman C, Hanis C, Harms M, Hiltunen M, Holi MM, Hultman CM, Kallela M, Kaprio J, Kathiresan S, Kim B-J, Kim YJ, Kirov G, Kooner J, Koskinen S, Krumholz HM, Kugathasan S, Kwak SH, Laakso M, Lehtimäki T, Loos RJF, Lubitz SA, Ma RCW, MacArthur DG, Marrugat J, Mattila KM, McCarroll S, McCarthy MI, McGovern D, McPherson R, Meigs JB, Melander O, Metspalu A, Neale BM, Nilsson PM, O'Donovan MC, Ongur D, Orozco L, Owen MJ, Palmer CNA, Palotie A, Park KS, Pato C, Pulver AE, Rahman N, Remes AM, Rioux JD, Ripatti S, Roden DM, Saleheen D, Salomaa V, Samani NJ, Scharf J, Schunkert H, Shoemaker MB, Sklar P, Soininen H, Sokol H, Spector T, Sullivan PF, Suvisaari J, Tai ES, Teo YY, Tiinamaija T, Tsuang M, Turner D, Tusie-Luna T, Vartiainen E, Vawter MP, Ware JS, Watkins H, Weersma RK, Wessman M, Wilson JG, Xavier RJ, Neale BM, Daly MJ, MacArthur DG. The mutational constraint spectrum quantified from variation in 141,456 humans. Nature. 2020;581(7809):434-43. doi: 10.1038/s41586-020-2308-7.

14. Bower JH, Maraganore DM, McDonnell SK, Rocca WA. Incidence and distribution of parkinsonism in Olmsted County, Minnesota, 1976-1990. Neurology. 1999;52(6):1214-. doi: 10.1212/WNL.52.6.1214.

15. Schrag A, Schott JM. Epidemiological, clinical, and genetic characteristics of early-onset parkinsonism. The Lancet Neurology. 2006;5(4):355-63. doi: 10.1016/S1474-4422(06)70411-2.

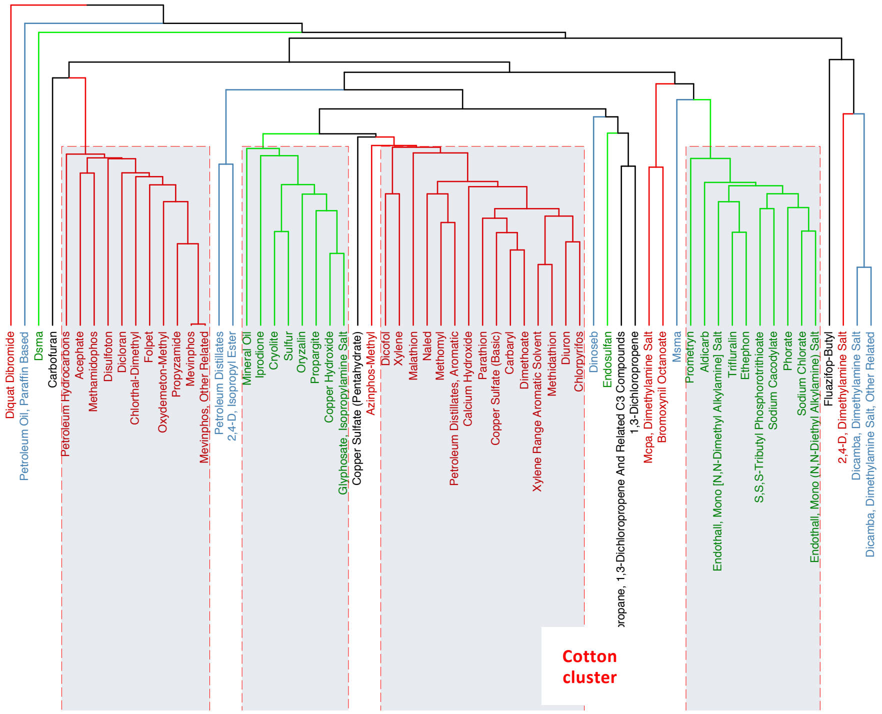

Supplementary Figure 1. Cotton Cluster. Results from hierarchical Pearson correlation clustering to group pesticides with correlated exposures. Cluster cut-point set at R≥0.45. Height is shown as 1-R.

Pesticides which were associated with PD at an FDR<0.05 in our previous untargeted analysis of proximity to agricultural pesticide application ingredients (Paul et al, 2023), were included in the clustering. Hierarchical clustering was done using the R hclust argument. We first obtained pairwise correlations for each pesticide pair. We then turned the correlations into distance measures by subtracting it from 1, meaning perfectly positively correlated pairs have distance 0. With this dissimilarity matrix, we then generated the hierarchical clustering using hclust and average-linkage, which puts every pesticide in its own cluster then begins iteratively merging the closest pairs based on the distance matrix. The threshold for merging was set at 0.55 (e.g. R≥0.45). The average-linkage calculates the average distance between pesticides in the clusters before merging.

a.

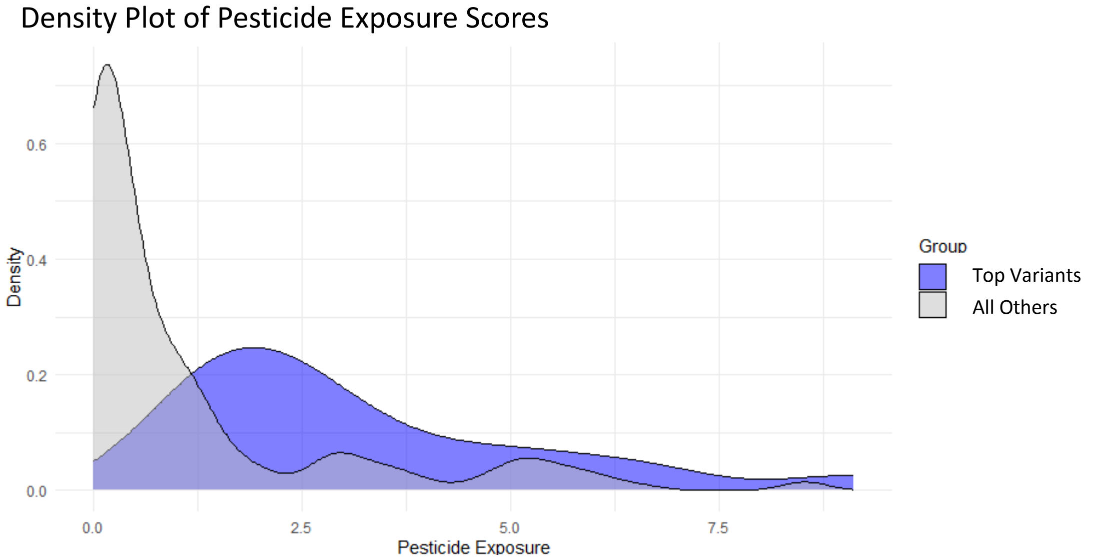

b.

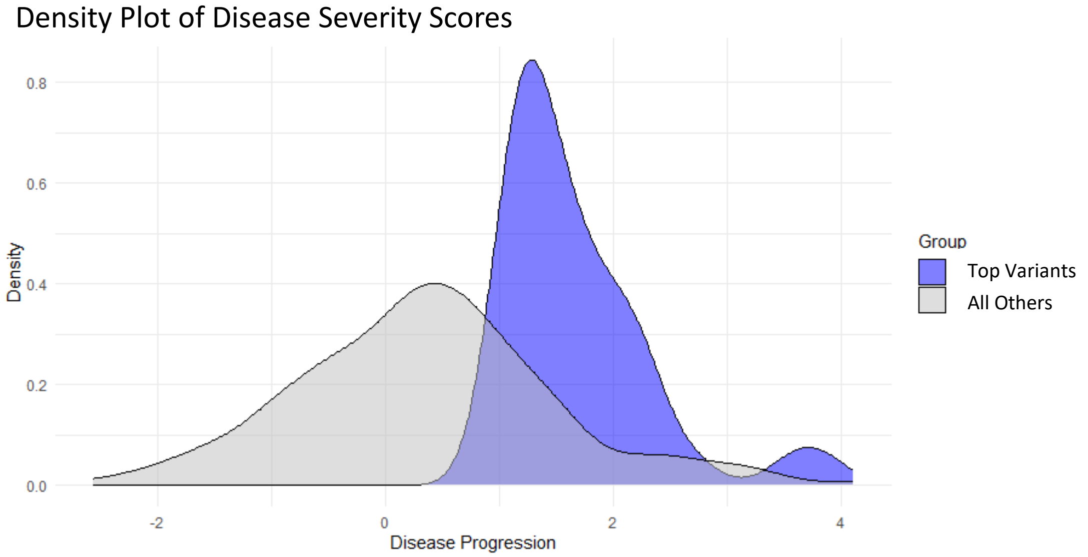

c.

Top Variants

	Disease Severity Score	Pesticide Exposure Score	Disease X Pesticide Score
max	3.850514885	9.111125264	0.538403837
min	1.009940779	1.006277778	0.029211969
average	1.671209062	3.191328698	0.145629801
std	0.65066158	2.180825655	0.121876276

All Others

	Disease Severity Score	Pesticide Exposure Score	Disease X Pesticide Score
max	4.104459397	8.524499177	0.113040862
min	-2.558526134	0	-0.160016712
average	0.366877121	1.157583438	0.002923086
std	1.133874169	1.757583535	0.040597196

Supplementary Figure 2. Pesticide Exposure and Disease Severity Score Metrics. a) A density plot is shown illustrating the distribution of pesticide exposure scores between the top 36 enriched variants (see text) and all other enriched variants. b) A density plot is shown illustrating the distribution of disease severity scores between the top 36 enriched variants (see text) and all other enriched variants. c). The distribution of pesticide exposure scores and disease severity scores between the top 36 enriched variants (see text) and all other enriched variants with corresponding disease X pesticide scores.

Supplementary Table 1

Group	*Gene Symbol*	Gene Name	Gene Function	Chromosomal Location	gnomAD pLI	gnomAD Missense Z-score
1. Known PD Risk Genes 14	*ACMSD*	Aminocarboxymuconate Semialdehyde Decarboxylase	NAD synthesis	2q21.3	0	0.01
	BST1	Bone Marrow Stromal Cell Antigen 1	Facilitates pre-B-cell growth	4p15.32	0	-0.1
	CCDC62	Coiled-Coil Domain Containing 62	Cellular response to estradiol stimulus	12q24.31	0	1.21
	GAK	Cyclin G Associated Kinase	Cyclin-dependent protein kinase	4p16.3	0.01	0.02
	GBA1 (GBA)	Glucosylceramidase Beta 1	Glycolipid metabolism	1q22	0	1.17
	HIP1R	Huntingtin Interacting Protein 1 Related	Phosphatidylinositol phosphate binding activity	12q24.31	0	-0.03
	HLA-DRB5	Major Histocompatibility Complex, Class II, DR Beta 5	Presents peptides for immune system	6p21.32	0	0.61
	LAMP3	Lysosomal Associated Membrane Protein 3	Differentiate dendritic cells	3q27.1	0	0.28
	LRRK2	Leucine Rich Repeat Kinase 2	Leucine-rich repeat kinase	12q12	0	1.26
	MAPT	Microtubule Associated Protein Tau	Maintain stability of microtubules in axons	17q21.31	0.01	1.52
	MCCC1	Methylcrotonyl-CoA Carboxylase Subunit 1	Catalyzes the carboxylation of 3-methylcrotonyl-CoA to form 3-methylglutaconyl-CoA	3q27.1	0	0.54
	SNCA	Synuclein Alpha	Integrate presynaptic signaling and membrane trafficking	4q22.1	0.88	0.46
	STK39	Serine/Threonine Kinase 39	Response to hypotonic stress	2q24.3	1	2.11
	SYT11	Synaptotagmin 11	Synaptic transmission	1q22	0.93	1.68
2. Lysosomal Function Genes 13	*ABHD5*	Abhydrolase Domain Containing 5, Lysophosphatidic Acid Acyltransferase	Acyltransferase	3p21.33	0	0.47
	ATG5	Autophagy Related 5	Autophagic vesicle formation	6q21	0.98	1.82
	ATG7	Autophagy Related 7	Autophagy and cytoplasmic to vacuole transport	3p25.3	0	1.37
	BAX	BCL2 Associated X, Apoptosis Regulator	Apoptotic activator	19q13.33	0.32	0.3
	BECN1	Beclin 1	Tumorigenesis, neurodegeneration and apoptosis	17q21.31	0.94	1.88
	CDKN2A	Cyclin Dependent Kinase Inhibitor 2A	Inhibit CDK4 kinase	9p21.3	0.39	-1.01
	FBXO7	F-Box Protein 7	SCFs (SKP1-cullin-F-box) ubiquitin protein ligase complex for phosphorylation-dependent ubiquitination	22q12.3	0	-0.44
	MAP1LC3A	Microtubule Associated Protein 1 Light Chain 3 Alpha	Mediate the physical interactions between microtubules and components of the cytoskeleton	20q11.22	0.03	1.46
	MAP1LC3B	Microtubule Associated Protein 1 Light Chain 3 Beta	Microtubule assembly and neurogenesis	16q24.2	0	0.29
	PINK1	PTEN Induced Kinase 1	Protect cells from stress-induced mitochondrial dysfunction	1p36.12	0	0.13
	PRKN (PARK2)	Parkin RBR E3 Ubiquitin Protein Ligase	Multiprotein E3 ubiquitin ligase complex mediate substrate proteins for proteasomal degradation	6q26	0	-0.86
	SH3GLB1	SH3 Domain Containing GRB2 Like, Endophilin B1	Interacts with the proapoptotic member of the Bcl-2 family, Bcl-2-associated X protein (Bax)	1p22.3	0.99	1.63
	TFEB	Transcription Factor EB	DNA-binding transcription factor activity	6p21.1	0.9	1.77
3. Lysosomal Function Genes that are PD Interactors 18	*APP*	Amyloid Beta Precursor Protein	Promote transcriptional activation	21q21.3	0.05	1.39
	BAG3	BAG Cochaperone 3	Binds to Hsc70/Hsp70 ATPase domain and promote substrate release	10q26.11	0.62	-0.74
	BAG6	BAG Cochaperone 6	Control of apoptosis and acetylation of p53 in response to DNA damage	6p21.33	1	2.72
	CRYAB	Crystallin Alpha B	Autokinase activity	11q23.1	0.02	0.48
	CTSD	Cathepsin D	Protein turnover and in the proteolytic activation of hormones and growth factors	11p15.5	0	1.38
	EP300	E1A Binding Protein P300	Regulates transcription via chromatin remodeling and cell proliferation and differentiation	22q13.2	1	2.03
	GSK3B	Glycogen Synthase Kinase 3 Beta	Regulate glucose homeostasis	3q13.33	0.96	2.8
	HDAC6	Histone Deacetylase 6	Alters chromosome structure and affects transcription factor access to DNA	Xp11.23	1	3.29
	HSP90AA1	Heat Shock Protein 90 Alpha Family Class A Member 1	Aids in target protein folding by use of an ATPase activity that is modulated by co-chaperones	14q32.31	0.86	1.04
	HSPA5	Heat Shock Protein Family A (Hsp70) Member 5	Regulator of ER homeostasis	9q33.3	0.77	4.03
	HSPA8	Heat Shock Protein Family A (Hsp70) Member 8	Binds to nascent polypeptides to facilitate correct folding	11q24.1	1	4.46
	HTT	Huntingtin	Regulate transcription and is required for normal development	4p16.3	1	2.78
	LAMP2	Lysosomal Associated Membrane Protein 2	Protection, maintenance, and adhesion of the lysosome	Xq24	0.27	0.84
	PRKCD	Protein Kinase C Delta	Tumor suppressor, regulator of cell cycle progression, and apoptosis	3p21.1	1	3.11
	PRNP	Prion Protein	Glycosylphosphatidylinositol-anchored glycoprotein	20p13	0	1.18
	PSEN1	Presenilin 1	Regulate APP processing and gamma-secretase activity	14q24.2	0.97	2.16
	STUB1	STIP1 Homology And U-box Containing Protein 1	Ubiquitin ligase/cochaperone in protein quality control for proteasomal degradation	16p13.3	0.02	1.16

	TP53	Tumor Protein P53	Induces cell cycle arrest, apoptosis, senescence, DNA repair, or changes in metabolism	17p13.1	0.53	0.98
4. Lysosomal Function Genes Highly Expressed in the Substantia Nigra 36	*ACP2*	Acid Phosphatase 2, Lysosomal	Hydrolyze orthophosphoric monoesters to alcohol and phosphate	11p11.2	0	0.83
	ARSA	Arylsulfatase A	Hydrolyzes cerebroside sulfate to cerebroside and sulfate	22q13.33	0	0.38
	ATG13	Autophagy Related 13	Autophagy factor and target of TOR kinase signaling pathway	11p11.2	0.96	1.52
	ATG3	Autophagy Related 3	Regulation of autophagy during cell death	3q13.2	0	1.76
	ATG4B	Autophagy Related 4B Cysteine Peptidase	Autophagy	2q37.3	0.96	1.41
	ATG4C	Autophagy Related 4C Cysteine Peptidase	Autophagy	1p31.3	0	-0.44
	ATG9A	Autophagy Related 9A	Autophagosome assembly	2q35	0.97	2.6
	ATP13A2	ATPase Cation Transporting 13A2	Transports inorganic cations as well as other substrates	1p36.13	0	1.4
	BLOC1S1	Biogenesis Of Lysosomal Organelles Complex 1 Subunit 1	Biogenesis of specialized organelles of the endosomal-lysosomal system	12q13.2	0.07	-0.03
	CHMP3	Charged Multivesicular Body Protein 3	Sorts transmembrane proteins into lysosomes/vacuoles via multivesicular body (MVB) pathway	2p11.2	0	0.8
	FLCN	Folliculin	Cellular response to amino acid availability and in the regulation of glycolysis	17p11.2	0.79	1.13
	GAA	Alpha Glucosidase	Degradation of glycogen to glucose in lysosomes	17q25.3	0	-0.63
	HDAC1	Histone Deacetylase 1	Control of cell proliferation and differentiation	1p35.2-p35.1	0.61	3.04
	HGS	Hepatocyte Growth Factor-Regulated Tyrosine Kinase Substrate	Regulates endosomal sorting and recycling and degradation of membrane receptors	17q25.3	0.01	1.28
	LAMP1	Lysosomal Associated Membrane Protein 1	Provides selectins with carbohydrate ligands	13q34	0.87	0.83
	LAPTM4B	Lysosomal Protein Transmembrane 4 Beta	Regulation of lysosomal membrane permeability and lysosome organization	8q22.1	0	-0.11
	MCOLN1	Mucolipin TRP Cation Channel 1	Regulation of lysosomal exocytosis	19p13.2	0	1.61
	MT3	Metallothionein 3	Growth inhibitory factor	16q13	0.06	0.66
	NPC1	NPC Intracellular Cholesterol Transporter 1	Mediates intracellular cholesterol trafficking	18q11.2	0	1.09
	PIK3R1	Phosphoinositide-3-Kinase Regulatory Subunit 1	Regulate metabolic actions of insulin	5q13.1	1	2.72
	PSAP	Prosaposin	Facilitate the catabolism of glycosphingolipids with short oligosaccharide groups	10q22.1	0.99	-0.52
	RAB1A	RAB1A, Member RAS Oncogene Family	Controls vesicle traffic from the endoplasmic reticulum to the Golgi apparatus	2p14	0.82	2.31
	RAB7A	RAB7A, Member RAS Oncogene Family	Regulates vesicle traffic in late endosomes and from late endosomes to lysosomes	3q21.3	0.97	2.28
	RB1CC1	RB1 Inducible Coiled-Coil 1	Regulate cell growth, cell proliferation, apoptosis, autophagy, and cell migration	8q11.23	1	0.81
	SMPD1	Sphingomyelin Phosphodiesterase 1	Converts sphingomyelin to ceramide	11p15.4	0	-0.1
	SNRPE	Small Nuclear Ribonucleoprotein Polypeptide E	3' end processing of histone transcripts	1q32.1	0.91	1.32
	TMBIM6	Transmembrane BAX Inhibitor Motif Containing 6	Regulation of RNA metabolic process, intrinsic apoptotic signaling pathway, and response to L-glutamate	12q13.12	0.02	0.59
	TP53INP2	Tumor Protein P53 Inducible Nuclear Protein 2	Promotes autophagy and autophagosome formation and processing	20q11.22	0.81	0.32
	TSC1	TSC Complex Subunit 1	Rapamycin complex 1 (mTORC1) signaling	9q34	1	2.32
	TSG101	Tumor Susceptibility 101	Regulates cell growth and differentiation, and negative growth regulator	11p15.1	0.34	2
	UBQLN1	Ubiquilin 1	Link ubiquitination machinery to proteasome to affect in vivo protein degradation	9q21.32	1	2.39
	ULK1	Unc-51 Like Autophagy Activating Kinase 1	Autophagosome assembly, host autophagy, and protein phosphorylation	12q24.33	0.99	1.07
	USP33	Ubiquitin Specific Peptidase 33	Slit-dependent cell migration and beta-2 adrenergic receptor signaling	1p31.1	0.86	2.1
	VCP	Valosin Containing Protein	DNA repair and replication, regulation of the cell cycle, and activation of the NF-kappa B pathway	9p13.3	1	5.41
	VPS39	VPS39 Subunit Of HOPS Complex	Promote clustering and fusion of late endosomes and lysosomes	15q15.1	0	2.65
	VTI1B	Vesicle Transport Through Interaction With T-SNAREs 1B	SNARE binding activity and chloride channel inhibitor activity	14q24.1	0.11	0.43
5. Other LSD Genes 4	*ASAH1*	N-Acylsphingosine Amidohydrolase 1	Catalyzes the degradation of ceramide into sphingosine and free fatty acid	8p22	0	-2.21
	GALC	Galactosylceramidase	Hydrolyzes galactose ester bonds of galactosylceramide, galactosylsphingosine, lactosylceramide, and monogalactosyldiglyceride	14q31.3	0	0.19
	GNPTAB	N-Acetylglucosamine-1-Phosphate Transferase Subunits Alpha And Beta	Catalyzes synthesis of mannose 6-phosphate recognition markers for trafficking of lysosomal enzymes	12q23.2	0	1.19
	SLC17A5	Solute Carrier Family 17 Member 5	Exports free sialic acids cleaved off of cell surface lipids and proteins from lysosomes	6q13	0	0.71

Supplementary Table 2

Gene	Genomic Change	Coding Change	Protein Change	Het	Hom	#Subjects
GBA1	1:155204793C>T	NM_001005742.3:c.1604G>A	NP_001005742.1:p.Arg535His	1	0	1
GBA1	1:155205043A>G	NM_001005742.3:c.1448T>C	NP_001005742.1:p.Leu483Pro	4	0	4
GBA1	1:155205518C>G	NM_001005742.3:c.1342G>C	NP_001005742.1:p.Asp448His	1	0	1
GBA1	1:155205634T>C	NM_001005742.3:c.1226A>G	NP_001005742.1:p.Asn409Ser	3	0	3
GBA1	1:155206037G>A	NM_001005742.3:c.1223C>T	NP_001005742.1:p.Thr408Met	12	0	12
GBA1	1:155206167C>T	NM_001005742.3:c.1093G>A	NP_001005742.1:p.Glu365Lys	27	0	27
GBA1	1:155207244C>T	NM_001005742.3:c.887G>A	NP_001005742.1:p.Arg296Gln	1	0	1
GBA1	1:155207965C>T	NM_001005742.3:c.721G>A	NP_001005742.1:p.Gly241Arg	1	0	1
GBA1	1:155208388G>A	NM_001005742.3:c.508C>T	NP_001005742.1:p.Arg170Cys	1	0	1
LRRK2	12:40713845G>C	NM_198578.4:c.4883G>C	NP_940980.4:p.Arg1628Pro	3	0	3
MAPT	17:44073889A>G	NM_001123066.3:c.1686A>G	NP_005901.2:p.Ala227=	227	13	240
MAPT	17:44073973T>C	NM_001123066.3:c.1770T>C	NP_005901.2:p.Asn255=	228	13	241

Supplementary Table 3

Gene	Genomic Change	Coding Change	Protein Change	Het	Hom	# Subjects
LRRK2	12:40734202G>A	NM_198578.4:c.6055G>A	NP_940980.4:p.Gly2019Ser	4	0	4
PRKN	6:162206852G>A	NM_004562.3:c.823C>T	NP_004553.2:p.Arg275Trp	3	0	3
PRKN	6:162206917C>T	NM_004562.3:c.758G>A	NP_004553.2:p.Cys253Tyr	1	0	1
PRKN	6:162864358_del	NM_004562.3:c.155delA	NP_004553.2:p.Asn52Metfs*29	0	2	2
PINK1	1:20972133T>C	NM_032409.3:c.1040T>C	NP_115785.1:p.Leu347Pro	1	0	1

Supplementary Table 4

Population	Gene Name	Genomic	HGVS c.	HGVS p.	CADD Score	#Het PPMI PD	#Hom PPMI PD	#Subjects PPMI PD	#Het PPMI HC	#Hom PPMI HC	#Subjects PPMI HC	European #Het PEG	European #HomoVar PEG	European #Subjects PEG	European PEG v gnomAD P-value	European PEG v PPMI PD P-value	European PEG vs.PPMI HC P-value	Hispanic #Het PEG	Hispanic #HomoVar PEG	Hispanic #Subjects PEG	Hispanic PEG v gnomAD P-value	Hispanic PEG v PPMI PD P-value	Hispanic PEG vs.PPMI HC P-value
Hispanic	ACMSD	2:135625179 G>A	NM_138326.3:c.517G>A	NP_612199.2:p.Val173Met	27.7	-	-	-	-	-	-	-	-	-	-	-	-	1	0	1	2.31E-09	-	-
Hispanic	ACP2	11:47266419_47266420insTG	NM_001610.4:c.640-3_640-2dupCA		22.9	-	-	-	-	-	-	-	-	-	-	-	-	3	0	3	2.90E-14	-	-
Hispanic	APP	21:27284122 T>C	NM_000484.4:c.1840A>G	NP_000475.1:p.Ser614Gly	21.3	-	-	-	-	-	-	-	-	-	-	-	-	1	0	1	2.83E-03	-	-
Hispanic	ASAH1	8:17927327 T>C	NM_004315.6:c.325A>G	NP_004306.3:p.Ile109Val	0.001	241	152	393	97	49	146	-	-	-	-	-	-	35	16	51	3.55E-04	1.10E-08	3.10E-09
Hispanic	ASAH1	8:17942197_17942203delAGCAAAG	NM_004315.6:c.108_114delCTTTGCT	NP_004306.3:p.Ser36Argfs*2	23.2	-	-	-	-	-	-	-	-	-	-	-	-	1	0	1	5.93E-06	-	-
Hispanic	ASAH1	8:17942273 C>T	NM_004315.6:c.38G>A	NP_004306.3:p.Gly13Glu	13.6	-	-	-	-	-	-	-	-	-	-	-	-	1	0	1	1.92E-04	-	-
Hispanic	ATG4C	1:63284955 G>C	NM_032852.4:c.674G>C	NP_116241.2:p.Gly225Ala	24.4	-	-	-	-	-	-	-	-	-	-	-	-	1	0	1	1.41E-04	-	-
Hispanic	ATG7	3:11402163 G>A	NM_006395.3:c.1588G>A	NP_006386.1:p.Ala530Thr	14.96	1	0	1	-	-	-	-	-	-	-	-	-	2	0	2	5.38E-03	1.49E-05	-
Hispanic	ATG9A	2:220085847 T>C	NM_001077198.3:c.2323A>G	NP_001070666.1:p.Thr775Ala	18.4	-	-	-	-	-	-	-	-	-	-	-	-	1	0	1	2.53E-02	-	-
Hispanic	BST1	4:15717385 G>A	NM_004334.3:c.667G>A	NP_004325.2:p.Glu223Lys	24.5	-	-	-	-	-	-	-	-	-	-	-	-	1	0	1	1.81E-52	-	-
Hispanic	CHMP3	2:86756394 T>C	NM_001198954.1:c.320A>G	NP_001185883.1:p.Tyr107Cys	24.5	-	-	-	-	-	-	-	-	-	-	-	-	1	0	1	1.38E-58	-	-
Hispanic	CTSD	11:1778666 T>C	NM_001909.5:c.592A>G	NP_001900.1:p.Ile198Val	19.43	-	-	-	-	-	-	-	-	-	-	-	-	1	0	1	5.40E-13	-	-
Hispanic	EP300	22:41542780 T>G	NM_001429.4:c.2091T>G	NP_001420.2:p.Ser697Arg	25.8	6	0	6	4	0	4	-	-	-	-	-	-	1	0	1	4.10E-02	3.78E-01	7.35E-01
Hispanic	EP300	22:41551055 C>T	NM_001429.4:c.3199C>T	NP_001420.2:p.Arg1067Cys	32	-	-	-	-	-	-	-	-	-	-	-	-	1	0	1	6.08E-59	-	-
Hispanic	FBXO7	22:32875209 G>A	NM_012179.4:c.364G>A	NP_036311.3:p.Asp122Asn	18.57	-	-	-	-	-	-	-	-	-	-	-	-	1	0	1	1.68E-07	-	-
Hispanic	FBXO7	22:32894494 G>C	NM_012179.4:c.1546G>C	NP_036311.3:p.Asp516His	24.7	1	0	1	2	0	2	-	-	-	-	-	-	1	0	1	1.76E-04	1.01E-02	3.56E-01
Hispanic	FBXO7	22:32894494 G>T	NM_012179.4:c.1546G>T	NP_036311.3:p.Asp516Tyr	25	-	-	-	-	-	-	-	-	-	-	-	-	1	0	1	4.55E-21	-	-
Hispanic	GAA	17:78082181 G>A	NM_000152.5:c.1048G>A	NP_000143.2:p.Val350Met	25.6	-	-	-	-	-	-	-	-	-	-	-	-	1	0	1	2.49E-60	-	-
Hispanic	GAA	17:78091484 C>T	NM_000152.5:c.2417C>T	NP_000143.2:p.Thr806Met	19.29	1	0	1	-	-	-	-	-	-	-	-	-	1	0	1	3.54E-02	1.05E-02	-
Hispanic	GAK	4:844728 A>C	NM_005255.4:c.3653T>G	NP_005246.2:p.Leu1218Arg	25.9	1	0	1	1	0	1	-	-	-	-	-	-	1	0	1	2.99E-02	1.05E-02	1.57E-01
Hispanic	GAK	4:860170 C>T	NM_005255.4:c.3025G>A	NP_005246.2:p.Ala1009Thr	0.004	-	-	-	-	-	-	-	-	-	-	-	-	1	0	1	5.72E-16	-	-
Hispanic	GAK	4:877843 T>C	NM_005255.4:c.1237A>G	NP_005246.2:p.Ile413Val	19.63	-	-	-	1	0	1	-	-	-	-	-	-	1	0	1	4.40E-03	-	1.57E-01
Hispanic	GAK	4:887799 T>G	NM_005255.4:c.742-2A>C		34	-	-	-	-	-	-	-	-	-	-	-	-	1	0	1	-	-	-
Hispanic	GBA1	1:155205043 A>G	NM_001005742.3:c.1448T>C	NP_001005742.1:p.Leu483Pro	23.6	-	-	-	2	0	2	-	-	-	-	-	-	1	0	1	2.72E-02	-	3.60E-01
Hispanic	GBA1	1:155209752 G>A	NM_001005742.3:c.232C>T	NP_001005742.1:p.Arg78Cys	23.8	2	0	2	-	-	-	-	-	-	-	-	-	1	0	1	8.43E-31	5.22E-02	-
Hispanic	GNPTAB	12:102164907 G>A	NM_024312.5:c.800C>T	NP_077288.2:p.Ala267Val	27	-	-	-	-	-	-	-	-	-	-	-	-	1	0	1	3.59E-04	-	-
Hispanic	HDAC1	1:32782306 A>G	NM_004964.3:c.203A>G	NP_004955.2:p.His68Arg	24.1	-	-	-	-	-	-	-	-	-	-	-	-	1	0	1	1.40E-09	-	-
Hispanic	HDAC6	X:48682972 A>G	NM_006044.4:c.3598A>G	NP_006035.2:p.Asn1200Asp	23.9	2	1	3	2	0	2	-	-	-	-	-	-	4	6	10	4.21E-02	5.00E-38	5.04E-18
Hispanic	HIP1R	12:123339915 T>C	NM_003959.3:c.956T>C	NP_003950.1:p.Ile319Thr	23.4	3	0	3	-	-	-	-	-	-	-	-	-	1	0	1	1.29E-02	1.20E-01	-
Hispanic	HIP1R	12:123342688 G>A	NM_003959.3:c.1855G>A	NP_003950.1:p.Glu619Lys	20.3	2	0	2	2	0	2	-	-	-	-	-	-	2	0	2	1.11E-09	2.92E-04	4.53E-02
Hispanic	HIP1R	12:123345509 G>T	NM_003959.3:c.2813G>T	NP_003950.1:p.Cys938Phe	24.1	30	1	31	22	0	22	-	-	-	-	-	-	5	0	5	1.98E-02	6.49E-02	5.66E-01
Hispanic	HSP90AA1	14:102551722 T>G	NM_001017963.3:c.942A>C	NP_001017963.2:p.Glu314Asp	22.9	-	-	-	-	-	-	-	-	-	-	-	-	1	0	1	1.36E-04	-	-
Hispanic	HTT	4:3101038 A>G	NM_002111.8:c.385A>G	NP_002102.4:p.Met129Val	22.9	-	-	-	-	-	-	-	-	-	-	-	-	1	0	1	2.20E-02	-	-
Hispanic	HTT	4:3132038 A>G	NM_002111.8:c.1874A>G	NP_002102.4:p.Gln625Arg	18.81	9	0	9	1	0	1	-	-	-	-	-	-	2	0	2	2.67E-02	1.01E-01	1.08E-02
Hispanic	HTT	4:3216894 A>G	NM_002111.8:c.7010A>G	NP_002102.4:p.Lys2337Arg	8.307	-	-	-	1	0	1	-	-	-	-	-	-	1	0	1	2.25E-03	-	1.57E-01
Hispanic	HTT	4:3234956 A>G	NM_002111.8:c.8332A>G	NP_002102.4:p.Ser2778Gly	26.6	-	-	-	-	-	-	-	-	-	-	-	-	1	0	1	9.57E-11	-	-
Hispanic	LAMP2	X:119581776 C>T	NM_002294.3:c.661G>A	NP_002285.1:p.Gly221Arg	24.2	-	-	-	0	2	2	-	-	-	-	-	-	0	1	1	2.82E-02	-	4.39E-01
Hispanic	LRRK2	12:40626147 G>C	NM_198578.4:c.309G>C	NP_940980.4:p.Gln103His	14.86	-	-	-	-	-	-	-	-	-	-	-	-	1	0	1	2.27E-04	-	-
Hispanic	LRRK2	12:40687426 G>C	NM_198578.4:c.2769G>C	NP_940980.4:p.Gln923His	14.21	1	0	1	-	-	-	-	-	-	-	-	-	1	0	1	5.46E-03	1.05E-02	-
Hispanic	MAPT	17:44049306 C>T	NM_001123066.4:c.215C>T	NP_001116538.2:p.Ala72Val	14.61	-	-	-	-	-	-	-	-	-	-	-	-	1	0	1	4.09E-30	-	-
Hispanic	MAPT	17:44068849 C>G	NM_001123066.4:c.1404C>G	NP_001116538.2:p.Ile468Met	21	-	-	-	-	-	-	-	-	-	-	-	-	1	0	1	1.77E-20	-	-
Hispanic	MCCC1	3:182775100 G>A	NM_020166.5:c.872C>T	NP_064551.3:p.Ala291Val	25.1	-	-	-	-	-	-	-	-	-	-	-	-	1	0	1	9.84E-06	-	-
Hispanic	NPC1	18:21112206 C>T	NM_000271.5:c.3797G>A	NP_000262.2:p.Arg1266Gln	16.46	29	0	29	27	0	27	-	-	-	-	-	-	16	3	19	4.75E-02	2.75E-28	7.67E-10
Hispanic	NPC1	18:21116700 A>G	NM_000271.5:c.3182T>C	NP_000262.2:p.Ile1061Thr	23.9	1	0	1	-	-	-	-	-	-	-	-	-	1	0	1	5.23E-11	1.05E-02	-
Hispanic	NPC1	18:21119839 C>T	NM_000271.5:c.2731G>A	NP_000262.2:p.Gly911Ser	23.7	-	-	-	-	-	-	-	-	-	-	-	-	1	0	1	6.60E-04	-	-
Hispanic	NPC1	18:21120489 C>A	NM_000271.5:c.2527G>T	NP_000262.2:p.Val843Leu	22.9	-	-	-	-	-	-	-	-	-	-	-	-	1	0	1	7.72E-07	-	-
Hispanic	NPC1	18:21134743 G>A	NM_000271.5:c.1532C>T	NP_000262.2:p.Thr511Met	24.6	-	-	-	-	-	-	-	-	-	-	-	-	1	0	1	4.39E-03	-	-
Hispanic	PIK3R1	5:67522783 C>T	NM_181523.3:c.280C>T	NP_852664.1:p.Pro94Ser	27.6	-	-	-	-	-	-	-	-	-	-	-	-	1	0	1	2.59E-08	-	-
Hispanic	PINK1	1:20975021 G>A	NM_032409.3:c.1147G>A	NP_115785.1:p.Ala383Thr	13.23	-	-	-	-	-	-	-	-	-	-	-	-	1	0	1	1.10E-04	-	-
Hispanic	PRKCD	3:53220000 A>G	NM_212539.2:c.1043A>G	NP_997704.1:p.Asn348Ser	17.5	-	-	-	-	-	-	-	-	-	-	-	-	1	0	1	6.09E-21	-	-
Hispanic	PRKN	6:162864358delT	NM_004562.3:c.155delA	NP_004553.2:p.Asn52Metfs*29	33	-	-	-	-	-	-	-	-	-	-	-	-	0	1	1	2.04E-06	-	-
Hispanic	PSAP	10:73580011 T>C	NM_002778.4:c.991A>G	NP_002769.1:p.Asn331Asp	14.38	-	-	-	-	-	-	-	-	-	-	-	-	1	0	1	-	-	-
Hispanic	RB1CC1	8:53558307 A>T	NM_014781.5:c.3942T>A	NP_055596.3:p.Asn1314Lys	23.6	8	0	8	2	0	2	-	-	-	-	-	-	3	0	3	6.99E-05	3.83E-03	4.06E-03
Hispanic	SMPD1	11:6415770_6415772delGCC	NM_001007593.3:c.1826_1828delGCC	NP_001007594.2:p.Arg609del	21.7	-	-	-	-	-	-	-	-	-	-	-	-	1	0	1	2.28E-04	-	-
Hispanic	TFEB	6:41653869 G>A	NM_007162.3:c.910C>T	NP_009093.1:p.Arg304Cys	31	-	-	-	-	-	-	-	-	-	-	-	-	1	0	1	5.24E-13	-	-
Hispanic	TSC1	9:135786013 G>A	NM_001162427.2:c.1055C>T	NP_001155899.1:p.Ser352Leu	21.2	-	-	-	-	-	-	-	-	-	-	-	-	1	0	1	5.23E-13	-	-
Hispanic	TSG101	11:18531139 C>T	NM_006292.4:c.431G>A	NP_006283.1:p.Arg144His	31	-	-	-	-	-	-	-	-	-	-	-	-	1	0	1	6.17E-59	-	-
Hispanic	UBQLN1	9:86297871 C>G	NM_053067.3:c.443G>C	NP_444295.1:p.Gly148Ala	18.9	-	-	-	-	-	-	-	-	-	-	-	-	1	0	1	1.23E-04	-	-
Hispanic	ULK1	12:132402007 G>T	NM_003565.4:c.2234G>T	NP_003556.2:p.Gly745Val	23.2	3	0	3	-	-	-	-	-	-	-	-	-	1	0	1	4.60E-02	1.20E-01	-
Hispanic	VPS39	15:42479957 T>G	NM_015289.5:c.440A>C	NP_056104.2:p.Gln147Pro	23	-	-	-	-	-	-	-	-	-	-	-	-	1	0	1	3.58E-04	-	-
Hispanic	VTI1B	14:68123147 G>A	NM_006370.3:c.526C>T	NP_006361.1:p.Arg176Cys	31	-	-	-	-	-	-	-	-	-	-	-	-	1	0	1	1.10E-12	-	-
European	ABHD5	3:43743718 A>G	NM_016006.6:c.145A>G	NP_057090.2:p.Thr49Ala	13.66	-	-	-	-	-	-	1	0	1	5.77E-14	-	-	-	-	-	-	-	-
European	ABHD5	3:43759162 G>A	NM_016006.6:c.774-1G>A		34	-	-	-	-	-	-	1	0	1	-	-	-	-	-	-	-	-	-
European	ACMSD	2:135621115 A>G	NM_138326.3:c.400A>G	NP_612199.2:p.Lys134Glu	21.3	1	0	1	-	-	-	1	0	1	3.36E-02	3.99E-01	-	-	-	-	-	-	-
European	ACMSD	2:135625174 T>C	NM_138326.3:c.512T>C	NP_612199.2:p.Leu171Pro	25.4	-	-	-	-	-	-	1	0	1	7.05E-21	-	-	-	-	-	-	-	-
European	ACMSD	2:135655939 C>T	NM_138326.3:c.878C>T	NP_612199.2:p.Pro293Leu	31	-	-	-	-	-	-	1	0	1	1.14E-03	-	-	-	-	-	-	-	-
European	ACP2	11:47261798 C>A	NM_001610.4:c.1141G>T	NP_001601.1:p.Val381Leu	21.4	-	-	-	-	-	-	1	0	1	4.16E-03	-	-	-	-	-	-	-	-
European	ACP2	11:47264289 C>T	NM_001610.4:c.1123G>A	NP_001601.1:p.Gly375Ser	2.021	-	-	-	-	-	-	1	0	1	2.90E-12	-	-	-	-	-	-	-	-
European	ACP2	11:47264290 G>C	NM_001610.4:c.1122C>G	NP_001601.1:p.Ser374Arg	7.479	-	-	-	-	-	-	1	0	1	-	-	-	-	-	-	-	-	-
European	ACP2	11:47264339 G>A	NM_001610.4:c.1073C>T	NP_001601.1:p.Thr358Ile	22.8	-	-	-	-	-	-	1	0	1	1.26E-06	-	-	-	-	-	-	-	-
European	ACP2	11:47264370 G>A	NM_001610.4:c.1042C>T	NP_001601.1:p.Arg348Cys	21.7	-	-	-	-	-	-	1	0	1	2.71E-12	-	-	-	-	-	-	-	-
European	ACP2	11:47267092 C>T	NM_001610.4:c.482G>A	NP_001601.1:p.Arg161His	24.5	-	-	-	-	-	-	1	0	1	7.01E-21	-	-	-	-	-	-	-	-
European	ACP2	11:47270231_47270232insT	NM_001610.4:c.109dupA	NP_001601.1:p.Thr37Asnfs*72	33	-	-	-	-	-	-	1	0	1	-	-	-	-	-	-	-	-	-
European	ACP2	11:47270253 C>T	NM_001610.4:c.88G>A	NP_001601.1:p.Ala30Thr	22.7	-	-	-	-	-	-	1	0	1	2.14E-20	-	-	-	-	-	-	-	-
European	APP	21:27269956 C>T	NM_000484.4:c.1993G>A	NP_000475.1:p.Glu665Lys	25.7	-	-	-	-	-	-	1	0	1	7.18E-21	-	-	-	-	-	-	-	-
European	APP	21:27394201_27394202insGGC	NM_000484.4:c.817_819dupGCC	NP_000475.1:p.Ala273dup	16.45	-	-	-	-	-	-	1	0	1	4.77E-12	-	-	-	-	-	-	-	-
European	ARSA	22:51063656 C>T	NM_000487.6:c.1447G>A	NP_000478.3:p.Glu483Lys	6.5	1	0	1	-	-	-	2	0	2	8.54E-04	8.80E-02	-	-	-	-	-	-	-
European	ARSA	22:51063661 C>T	NM_000487.6:c.1442G>A	NP_000478.3:p.Arg481Gln	5.112	-	-	-	-	-	-	1	0	1	1.18E-10	-	-	-	-	-	-	-	-
European	ARSA	22:51064019 A>G	NM_000487.6:c.1198T>C	NP_000478.3:p.Phe400Leu	27.6	-	-	-	-	-	-	1	0	1	-	-	-	-	-	-	-	-	-
Both	ARSA	22:51064087 A>G	NM_000487.6:c.1130T>C	NP_000478.3:p.Phe377Ser	27.4	-	-	-	-	-	-	22	0	22	-	-	-	12	0	12	-	-	-
European	ARSA	22:51065361 C>A	NM_000487.6:c.585G>T	NP_000478.3:p.Trp195Cys	24.4	80	3	8.30E+01	27	0	27	62	6	68	2.23E-04	3.04E-11	5.66E-09	-	-	-	-	-	-
European	ARSA	22:51065755delG	NM_000487.6:c.304delC	NP_000478.3:p.Leu102Cysfs*6	25.3	-	-	-	-	-	-	1	0	1	-	-	-	-	-	-	-	-	-
European	ARSA	22:51066194 G>A	NM_000487.6:c.14C>T	NP_000478.3:p.Ala5Val	10.13	-	-	-	-	-	-	1	0	1	7.45E-03	-	-	-	-	-	-	-	-
European	ASAH1	8:17915075 G>A	NM_004315.6:c.1204C>T	NP_004306.3:p.Arg402Trp	32	-	-	-	-	-	-	1	0	1	7.14E-21	-	-	-	-	-	-	-	-
European	ASAH1	8:17917141 T>C	NM_004315.6:c.905A>G	NP_004306.3:p.Asn302Ser	18.85	-	-	-	-	-	-	1	0	1	5.96E-06	-	-	-	-	-	-	-	-
European	ASAH1	8:17919919 T>C	NM_004315.6:c.565A>G	NP_004306.3:p.Asn189Asp	27	-	-	-	-	-	-	1	0	1	-	-	-	-	-	-	-	-	-
European	ATG13	11:46690985 C>T	NM_001205119.2:c.1478C>T	NP_001192048.1:p.Pro493Leu	31	-	-	-	-	-	-	1	0	1	7.01E-21	-	-	-	-	-	-	-	-
Both	ATG3	3:112267485 G>A	NM_022488.5:c.238C>T	NP_071933.2:p.Pro80Ser	24.6	-	-	-	-	-	-	77	0	77	-	-	-	21	0	21	-	-	-
Both	ATG3	3:112267488 C>A	NM_022488.5:c.236-1G>T		35	-	-	-	-	-	-	319	0	319	-	-	-	66	0	66	-	-	-
Both	ATG3	3:112267489 T>A	NM_022488.5:c.236-2A>T		35	-	-	-	-	-	-	264	0	264	-	-	-	56	0	56	-	-	-
European	ATG4B	2:242590449 G>A	NM_178326.3:c.35G>A	NP_847896.1:p.Arg12Gln	23.2	-	-	-	-	-	-	1	0	1	6.74E-11	-	-	-	-	-	-	-	-
European	ATG4B	2:242590694delT	NM_178326.3:c.128delT	NP_847896.1:p.Leu43Cysfs*39	24.3	-	-	-	-	-	-	1	0	1	-	-	-	-	-	-	-	-	-
European	ATG4B	2:242606080 G>A	NM_178326.3:c.559G>A	NP_847896.1:p.Val187Ile	1.74	3	0	3	4	0	4	9	1	10	2.23E-02	2.21E-06	2.92E-02	-	-	-	-	-	-
Both	ATG4C	1:63299734_63299735delTG	NM_032852.4:c.959_960delTG	NP_116241.2:p.Val320Glyfs*8	33	-	-	-	1	0	1	4	0	4	1.67E-02	-	1.20E-01	2	0	2	1.22E-03	-	1.08E-02
European	ATG4C	1:63300486 C>G	NM_032852.4:c.1052C>G	NP_116241.2:p.Ser351Cys	27.1	-	-	-	-	-	-	1	0	1	-	-	-	-	-	-	-	-	-
European	ATG4C	1:63329808 C>T	NM_032852.4:c.1355C>T	NP_116241.2:p.Thr452Met	16.36	-	-	-	-	-	-	1	0	1	7.90E-10	-	-	-	-	-	-	-	-
European	ATG7	3:11389387 G>A	NM_006395.3:c.1162G>A	NP_006386.1:p.Ala388Thr	29.9	1	0	1	-	-	-	2	0	2	8.77E-04	8.80E-02	-	-	-	-	-	-	-
European	ATG7	3:11399910 A>G	NM_006395.3:c.1303A>G	NP_006386.1:p.Met435Val	23.2	-	-	-	-	-	-	1	0	1	1.23E-06	-	-	-	-	-	-	-	-
European	ATG7	3:11421464 C>T	NM_006395.3:c.1894C>T	NP_006386.1:p.Arg632Trp	32	-	-	-	-	-	-	1	0	1	2.21E-05	-	-	-	-	-	-	-	-
European	ATG9A	2:220085477 C>A	NM_001077198.3:c.2506G>T	NP_001070666.1:p.Val836Leu	22.5	-	-	-	-	-	-	1	0	1	5.43E-03	-	-	-	-	-	-	-	-
European	ATG9A	2:220087040 C>T	NM_001077198.3:c.1981G>A	NP_001070666.1:p.Gly661Arg	13.74	-	-	-	-	-	-	1	0	1	2.08E-08	-	-	-	-	-	-	-	-
European	ATG9A	2:220087458 C>T	NM_001077198.3:c.1757G>A	NP_001070666.1:p.Arg586Gln	26.2	-	-	-	-	-	-	1	0	1	1.66E-08	-	-	-	-	-	-	-	-
European	ATP13A2	1:17318282 G>A	NM_022089.4:c.2198C>T	NP_071372.1:p.Thr733Met	22.3	1	0	1	-	-	-	1	0	1	8.59E-46	3.99E-01	-	-	-	-	-	-	-
European	ATP13A2	1:17318873 C>T	NM_022089.4:c.1870G>A	NP_071372.1:p.Val624Ile	5.982	-	-	-	-	-	-	1	0	1	1.57E-07	-	-	-	-	-	-	-	-
European	ATP13A2	1:17322780 A>G	NM_022089.4:c.1322T>C	NP_071372.1:p.Ile441Thr	22.4	-	-	-	-	-	-	1	0	1	-	-	-	-	-	-	-	-	-
European	ATP13A2	1:17328821 T>A	NM_022089.4:c.605A>T	NP_071372.1:p.His202Leu	9.687	-	-	-	-	-	-	1	0	1	-	-	-	-	-	-	-	-	-
Both	ATP13A2	1:17332264 G>A	NM_022089.4:c.20C>T	NP_071372.1:p.Pro7Leu	18.47	-	-	-	-	-	-	62	0	62	-	-	-	12	0	12	-	-	-
European	BAG3	10:121431866 C>T	NM_004281.4:c.607C>T	NP_004272.2:p.Arg203Trp	29.4	-	-	-	-	-	-	1	0	1	2.25E-40	-	-	-	-	-	-	-	-
European	BAG3	10:121432002 A>G	NM_004281.4:c.743A>G	NP_004272.2:p.His248Arg	23.1	-	-	-	-	-	-	1	0	1	6.07E-11	-	-	-	-	-	-	-	-
European	BAG3	10:121436256 C>G	NM_004281.4:c.1190C>G	NP_004272.2:p.Ala397Gly	22.8	-	-	-	-	-	-	1	0	1	-	-	-	-	-	-	-	-	-
European	BAG3	10:121436279 G>T	NM_004281.4:c.1213G>T	NP_004272.2:p.Ala405Ser	10.3	-	-	-	-	-	-	1	0	1	-	-	-	-	-	-	-	-	-
European	BAG6	6:31607374 C>T	NM_080703.3:c.3188G>A	NP_542434.1:p.Arg1063Gln	22.5	-	-	-	-	-	-	1	0	1	2.61E-40	-	-	-	-	-	-	-	-
European	BAG6	6:31611890 T>C	NM_080703.3:c.1529A>G	NP_542434.1:p.His510Arg	22.7	-	-	-	-	-	-	1	0	1	-	-	-	-	-	-	-	-	-
European	BAG6	6:31612356 C>T	NM_080703.3:c.1393G>A	NP_542434.1:p.Val465Ile	22.7	-	-	-	-	-	-	1	0	1	-	-	-	-	-	-	-	-	-
European	BAG6	6:31612806 G>A	NM_080703.3:c.1286C>T	NP_542434.1:p.Pro429Leu	23.2	-	-	-	-	-	-	0	1	1	7.72E-88	-	-	-	-	-	-	-	-
European	BAG6	6:31614212 C>T	NM_080703.3:c.877G>A	NP_542434.1:p.Glu293Lys	24.2	-	-	-	-	-	-	1	0	1	8.78E-04	-	-	-	-	-	-	-	-
European	BAG6	6:31615477 G>T	NM_080703.3:c.679C>A	NP_542434.1:p.Pro227Thr	21	-	-	-	-	-	-	1	0	1	1.55E-08	-	-	-	-	-	-	-	-
European	BAG6	6:31616513 G>A	NM_080703.3:c.493C>T	NP_542434.1:p.Arg165Trp	32	-	-	-	-	-	-	1	0	1	4.68E-16	-	-	-	-	-	-	-	-
European	BAX	19:49459468 G>A	NM_004324.4:c.247G>A	NP_004315.1:p.Val83Met	18.02	-	-	-	-	-	-	1	0	1	7.94E-04	-	-	-	-	-	-	-	-
European	BAX	19:49464172 G>A	NM_004324.4:c.475G>A	NP_004315.1:p.Val159Met	29.8	-	-	-	-	-	-	1	0	1	-	-	-	-	-	-	-	-	-
European	BLOC1S1	12:56110773 G>A	NM_001487.4:c.202G>A	NP_001478.2:p.Asp68Asn	32	-	-	-	-	-	-	3	0	3	4.36E-02	-	-	-	-	-	-	-	-
European	BLOC1S1	12:56112974 C>G	NM_001487.4:c.318C>G	NP_001478.2:p.Ile106Met	17.04	-	-	-	-	-	-	1	0	1	-	-	-	-	-	-	-	-	-
European	BST1	4:15709246 G>A	NM_004334.3:c.428G>A	NP_004325.2:p.Trp143Ter	40	-	-	-	-	-	-	1	0	1	5.63E-11	-	-	-	-	-	-	-	-
European	CCDC62	12:123281928 C>T	NM_201435.5:c.847C>T	NP_958843.2:p.His283Tyr	23.2	-	-	-	-	-	-	1	0	1	3.82E-04	-	-	-	-	-	-	-	-
European	CDKN2A	9:21971122 G>C	NM_001195132.2:c.236C>G	NP_001182061.1:p.Thr79Ser	22.1	-	-	-	-	-	-	1	0	1	-	-	-	-	-	-	-	-	-
European	CTSD	11:1782594 G>A	NM_001909.5:c.173C>T	NP_001900.1:p.Ala58Val	0.974	81	2	83	30	3	33	39	0	39	4.97E-02	6.07E-02	2.42E-01	-	-	-	-	-	-
European	EP300	22:41489027 G>C	NM_001429.4:c.19G>C	NP_001420.2:p.Glu7Gln	25.4	-	-	-	-	-	-	1	0	1	-	-	-	-	-	-	-	-	-
European	EP300	22:41521890 A>G	NM_001429.4:c.752A>G	NP_001420.2:p.Asn251Ser	9.748	-	-	-	-	-	-	1	0	1	2.98E-02	-	-	-	-	-	-	-	-
European	EP300	22:41545804 A>G	NM_001429.4:c.2419A>G	NP_001420.2:p.Ile807Val	16.44	-	-	-	-	-	-	1	0	1	5.61E-09	-	-	-	-	-	-	-	-
European	EP300	22:41547957 G>A	NM_001429.4:c.2938G>A	NP_001420.2:p.Ala980Thr	13.1	-	-	-	-	-	-	1	0	1	-	-	-	-	-	-	-	-	-
European	EP300	22:41565569 C>T	NM_001429.4:c.4235C>T	NP_001420.2:p.Ala1412Val	28.7	-	-	-	-	-	-	1	0	1	2.82E-14	-	-	-	-	-	-	-	-
European	EP300	22:41572401 C>T	NM_001429.4:c.4930C>T	NP_001420.2:p.Leu1644Phe	23.5	-	-	-	-	-	-	1	0	1	2.72E-14	-	-	-	-	-	-	-	-
European	EP300	22:41573672 C>T	NM_001429.4:c.5957C>T	NP_001420.2:p.Pro1986Leu	22.9	-	-	-	-	-	-	1	0	1	4.72E-05	-	-	-	-	-	-	-	-
European	EP300	22:41574383 A>C	NM_001429.4:c.6668A>C	NP_001420.2:p.Gln2223Pro	20.4	26	1	27	12	0	12	12	0	12	2.42E-02	4.05E-01	6.52E-01	-	-	-	-	-	-
European	EP300	22:41574511_41574513delCAG	NM_001429.4:c.6798_6800delGCA	NP_001420.2:p.Gln2268del	21.8	4	0	4	-	-	-	6	0	6	8.46E-03	8.61E-03	-	-	-	-	-	-	-
European	EP300	22:41574951 C>G	NM_001429.4:c.7236C>G	NP_001420.2:p.Asp2412Glu	22.3	-	-	-	-	-	-	1	0	1	8.40E-21	-	-	-	-	-	-	-	-
European	FBXO7	22:32881154 A>C	NM_012179.4:c.745A>C	NP_036311.3:p.Thr249Pro	14.3	-	-	-	-	-	-	1	0	1	-	-	-	-	-	-	-	-	-
European	FBXO7	22:32894401 G>A	NM_012179.4:c.1453G>A	NP_036311.3:p.Val485Ile	0.009	1	0	1	-	-	-	2	0	2	1.71E-02	8.80E-02	-	-	-	-	-	-	-
European	FBXO7	22:32894443 G>A	NM_012179.4:c.1495G>A	NP_036311.3:p.Gly499Ser	22.9	-	-	-	-	-	-	1	0	1	2.99E-03	-	-	-	-	-	-	-	-
European	FLCN	17:17118542 G>C	NM_144997.7:c.1389C>G	NP_659434.2:p.Tyr463Ter	33	-	-	-	-	-	-	1	0	1	-	-	-	-	-	-	-	-	-
European	FLCN	17:17124920 G>A	NM_144997.7:c.802C>T	NP_659434.2:p.Arg268Trp	28.7	-	-	-	-	-	-	1	0	1	5.92E-10	-	-	-	-	-	-	-	-
European	FLCN	17:17127268 T>C	NM_144997.7:c.586A>G	NP_659434.2:p.Ile196Val	16.51	-	-	-	-	-	-	1	0	1	3.08E-12	-	-	-	-	-	-	-	-
European	GAA	17:78078516 G>T	NM_000152.5:c.131G>T	NP_000143.2:p.Gly44Val	9.383	-	-	-	-	-	-	1	0	1	3.94E-02	-	-	-	-	-	-	-	-
European	GAA	17:78078651 G>A	NM_000152.5:c.266G>A	NP_000143.2:p.Arg89His	26.2	-	-	-	-	-	-	1	0	1	4.39E-03	-	-	-	-	-	-	-	-
European	GAA	17:78081458 T>A	NM_000152.5:c.795T>A	NP_000143.2:p.Ser265Arg	11.02	-	-	-	-	-	-	1	0	1	6.18E-11	-	-	-	-	-	-	-	-
European	GAA	17:78082512 A>G	NM_000152.5:c.1211A>G	NP_000143.2:p.Asp404Gly	24.6	-	-	-	-	-	-	1	0	1	-	-	-	-	-	-	-	-	-
European	GAA	17:78086445 G>T	NM_000152.5:c.1823G>T	NP_000143.2:p.Arg608Leu	6.212	-	-	-	-	-	-	1	0	1	-	-	-	-	-	-	-	-	-
European	GAA	17:78090815 G>C	NM_000152.5:c.2238G>C	NP_000143.2:p.Trp746Cys	32	-	-	-	-	-	-	2	0	2	7.34E-03	-	-	-	-	-	-	-	-
European	GAK	4:843771 C>T	NM_005255.4:c.3743G>A	NP_005246.2:p.Arg1248His	25.6	-	-	-	-	-	-	1	0	1	6.94E-14	-	-	-	-	-	-	-	-
European	GAK	4:845630 G>C	NM_005255.4:c.3416C>G	NP_005246.2:p.Pro1139Arg	15.25	2	0	2	-	-	-	1	0	1	1.56E-06	7.18E-01	-	-	-	-	-	-	-
European	GAK	4:862455 C>A	NM_005255.4:c.2267G>T	NP_005246.2:p.Arg756Leu	24.2	-	-	-	-	-	-	1	0	1	1.57E-22	-	-	-	-	-	-	-	-
European	GAK	4:870891 C>T	NM_005255.4:c.1961G>A	NP_005246.2:p.Arg654Gln	26.2	-	-	-	-	-	-	1	0	1	3.44E-02	-	-	-	-	-	-	-	-
European	GAK	4:871472 C>T	NM_005255.4:c.1787G>A	NP_005246.2:p.Arg596Lys	28.8	1	0	1	-	-	-	1	0	1	5.56E-10	3.99E-01	-	-	-	-	-	-	-
European	GAK	4:882731 G>A	NM_005255.4:c.1109C>T	NP_005246.2:p.Pro370Leu	22	-	-	-	1	0	1	2	0	2	2.42E-02	-	4.60E-01	-	-	-	-	-	-
European	GAK	4:884350 C>T	NM_005255.4:c.1051G>A	NP_005246.2:p.Val351Met	11.82	-	-	-	-	-	-	1	0	1	3.69E-44	-	-	-	-	-	-	-	-
European	GAK	4:887275 C>T	NM_005255.4:c.880G>A	NP_005246.2:p.Ala294Thr	22.3	-	-	-	-	-	-	2	0	2	1.60E-06	-	-	-	-	-	-	-	-
European	GAK	4:887700 G>A	NM_005255.4:c.839C>T	NP_005246.2:p.Pro280Leu	6.538	1	0	1	-	-	-	1	0	1	1.66E-03	3.99E-01	-	-	-	-	-	-	-
European	GAK	4:905556 G>A	NM_005255.4:c.287C>T	NP_005246.2:p.Pro96Leu	24.7	-	-	-	-	-	-	1	0	1	6.95E-21	-	-	-	-	-	-	-	-
European	GALC	14:88411981 G>A	NM_001201401.2:c.1517C>T	NP_001188330.1:p.Thr506Met	24.7	-	-	-	-	-	-	1	0	1	1.27E-02	-	-	-	-	-	-	-	-
European	GALC	14:88452845delT	NM_001201401.2:c.361delA	NP_001188330.1:p.Ile121Leufs*27	32	-	-	-	-	-	-	1	0	1	6.21E-09	-	-	-	-	-	-	-	-
European	GALC	14:88452941 T>C	NM_001201401.2:c.265A>G	NP_001188330.1:p.Thr89Ala	24.4	5	0	5	3	0	3	7	0	7	6.04E-03	6.06E-03	1.18E-01	-	-	-	-	-	-
European	GBA1	1:155206165 C>G	NM_001005742.3:c.1095G>C	NP_001005742.1:p.Glu365Asp	15.59	-	-	-	-	-	-	1	0	1	-	-	-	-	-	-	-	-	-
European	GBA1	1:155207965 C>T	NM_001005742.3:c.721G>A	NP_001005742.1:p.Gly241Arg	23.5	-	-	-	-	-	-	1	0	1	5.59E-09	-	-	-	-	-	-	-	-
European	GBA1	1:155208423 A>G	NM_001005742.3:c.473T>C	NP_001005742.1:p.Ile158Thr	22.8	-	-	-	-	-	-	1	0	1	-	-	-	-	-	-	-	-	-
European	GNPTAB	12:102147227 C>A	NM_024312.5:c.3525G>T	NP_077288.2:p.Met1175Ile	25.8	-	-	-	-	-	-	1	0	1	2.78E-14	-	-	-	-	-	-	-	-
European	GNPTAB	12:102158892 T>C	NM_024312.5:c.1803A>G	NP_077288.2:p.Ile601Met	14.8	-	-	-	-	-	-	1	0	1	1.60E-23	-	-	-	-	-	-	-	-
European	GSK3B	3:119545685 G>A	NM_002093.4:c.1252C>T	NP_002084.2:p.Arg418Cys	29.3	-	-	-	-	-	-	1	0	1	7.27E-21	-	-	-	-	-	-	-	-
European	GSK3B	3:119585455 A>T	NM_002093.4:c.930T>A	NP_002084.2:p.His310Gln	21.4	-	-	-	-	-	-	1	0	1	3.11E-02	-	-	-	-	-	-	-	-
European	HDAC1	1:32798331_32798333delGAG	NM_004964.3:c.1402_1404delGAG	NP_004955.2:p.Glu468del	22.4	1	0	1	-	-	-	1	0	1	1.23E-05	3.99E-01	-	-	-	-	-	-	-
European	HDAC6	X:48661360 T>C	NM_006044.4:c.176T>C	NP_006035.2:p.Leu59Pro	24.9	-	-	-	-	-	-	0	1	1	3.06E-03	-	-	-	-	-	-	-	-
European	HDAC6	X:48674577 T>C	NM_006044.4:c.1523T>C	NP_006035.2:p.Leu508Ser	1.232	-	-	-	-	-	-	1	0	1	5.34E-05	-	-	-	-	-	-	-	-
European	HDAC6	X:48681339 G>A	NM_006044.4:c.2530G>A	NP_006035.2:p.Gly844Arg	11.87	1	0	1	-	-	-	1	0	1	7.30E-22	2.49E-01	-	-	-	-	-	-	-
European	HGS	17:79660570 C>T	NM_004712.5:c.700C>T	NP_004703.1:p.Pro234Ser	24.5	1	0	1	-	-	-	1	0	1	8.68E-03	3.99E-01	-	-	-	-	-	-	-
European	HGS	17:79660590 C>A	NM_004712.5:c.720C>A	NP_004703.1:p.Ser240Arg	25.9	1	0	1	-	-	-	1	0	1	4.34E-02	3.99E-01	-	-	-	-	-	-	-
European	HIP1R	12:123341703 C>T	NM_003959.3:c.1756C>T	NP_003950.1:p.Arg586Trp	24.1	1	0	1	-	-	-	1	0	1	2.32E-07	3.99E-01	-	-	-	-	-	-	-
European	HIP1R	12:123341749 G>A	NM_003959.3:c.1802G>A	NP_003950.1:p.Arg601Gln	5.354	-	-	-	-	-	-	1	0	1	2.82E-02	-	-	-	-	-	-	-	-
European	HIP1R	12:123342671 G>A	NM_003959.3:c.1838G>A	NP_003950.1:p.Arg613Gln	9.163	-	-	-	-	-	-	1	0	1	4.44E-23	-	-	-	-	-	-	-	-
European	HIP1R	12:123345975 C>T	NM_003959.3:c.3073C>T	NP_003950.1:p.Arg1025Trp	21.3	-	-	-	-	-	-	1	0	1	4.17E-03	-	-	-	-	-	-	-	-
Both	HLA-DRB5	6:32486354 T>G	NM_002125.4:c.742A>C	NP_002116.2:p.Ile248Leu	23.4	-	-	-	-	-	-	29	0	29	-	-	-	3	0	3	-	-	-
Both	HLA-DRB5	6:32487158_32487159insCA	NM_002125.4:c.640_641insTG	NP_002116.2:p.Thr214Metfs*17	25.1	-	-	-	-	-	-	167	0	167	-	-	-	30	0	30	-	-	-
Both	HLA-DRB5	6:32487164 G>A	NM_002125.4:c.635C>T	NP_002116.2:p.Pro212Leu	20.9	-	-	-	-	-	-	167	0	167	-	-	-	30	0	30	-	-	-
Both	HLA-DRB5	6:32487189 C>G	NM_002125.4:c.610G>C	NP_002116.2:p.Val204Leu	26.2	-	-	-	-	-	-	167	0	167	-	-	-	30	0	30	-	-	-
Both	HLA-DRB5	6:32487209 C>G	NM_002125.4:c.590G>C	NP_002116.2:p.Gly197Ala	25.6	-	-	-	-	-	-	167	0	167	-	-	-	30	0	30	-	-	-
Both	HLA-DRB5	6:32487212 C>T	NM_002125.4:c.587G>A	NP_002116.2:p.Ser196Asn	22.3	-	-	-	-	-	-	167	0	167	-	-	-	30	0	30	-	-	-
Both	HLA-DRB5	6:32487333 T>C	NM_002125.4:c.466A>G	NP_002116.2:p.Ile156Val	20.3	-	-	-	-	-	-	5	0	5	-	-	-	1	0	1	-	-	-
Both	HLA-DRB5	6:32489935 C>G	NM_002125.4:c.117G>C	NP_002116.2:p.Gln39His	16.05	-	-	-	-	-	-	81	12	93	-	-	-	23	1	24	-	-	-
Both	HLA-DRB5	6:32489937 G>A	NM_002125.4:c.115C>T	NP_002116.2:p.Gln39Ter	67	-	-	-	-	-	-	74	12	86	-	-	-	23	1	24	-	-	-
European	HLA-DRB5	6:32497904 C>T	NM_002125.4:c.98G>A	NP_002116.2:p.Arg33Gln	0.53	-	-	-	-	-	-	70	0	70	6.82E-07	-	-	-	-	-	-	-	-
Both	HLA-DRB5	6:32497928 G>C	NM_002125.4:c.74C>G	NP_002116.2:p.Pro25Arg	19.77	-	-	-	-	-	-	40	55	95	-	-	-	7	15	22	-	-	-
Both	HLA-DRB5	6:32497986 G>A	NM_002125.4:c.16C>T	NP_002116.2:p.Leu6Phe	1.328	-	-	-	-	-	-	55	0	55	-	-	-	16	0	16	-	-	-
European	HSPA8	11:122930585 T>C	NM_006597.6:c.716A>G	NP_006588.1:p.Asn239Ser	22.7	-	-	-	-	-	-	1	0	1	7.55E-21	-	-	-	-	-	-	-	-
European	HTT	4:3101073 G>C	NM_002111.8:c.420G>C	NP_002102.4:p.Glu140Asp	22.8	-	-	-	-	-	-	1	0	1	5.36E-03	-	-	-	-	-	-	-	-
European	HTT	4:3129281 A>G	NM_002111.8:c.1693A>G	NP_002102.4:p.Thr565Ala	25	-	-	-	-	-	-	1	0	1	-	-	-	-	-	-	-	-	-
European	HTT	4:3133442 C>T	NM_002111.8:c.2176C>T	NP_002102.4:p.His726Tyr	13.16	-	-	-	-	-	-	1	0	1	1.84E-05	-	-	-	-	-	-	-	-
European	HTT	4:3134324 G>A	NM_002111.8:c.2272G>A	NP_002102.4:p.Asp758Asn	22	-	-	-	-	-	-	1	0	1	2.30E-40	-	-	-	-	-	-	-	-
European	HTT	4:3138013 G>A	NM_002111.8:c.2758G>A	NP_002102.4:p.Asp920Asn	32	-	-	-	-	-	-	2	0	2	2.92E-02	-	-	-	-	-	-	-	-
European	HTT	4:3189553 C>T	NM_002111.8:c.5165C>T	NP_002102.4:p.Thr1722Met	4.719	-	-	-	-	-	-	1	0	1	5.83E-04	-	-	-	-	-	-	-	-
European	HTT	4:3210510 C>T	NM_002111.8:c.6163C>T	NP_002102.4:p.Leu2055Phe	22.7	1	0	1	-	-	-	1	0	1	4.88E-02	3.99E-01	-	-	-	-	-	-	-
European	HTT	4:3214342 A>G	NM_002111.8:c.6680A>G	NP_002102.4:p.Gln2227Arg	24.1	-	-	-	-	-	-	1	0	1	8.38E-21	-	-	-	-	-	-	-	-
European	HTT	4:3231719 G>A	NM_002111.8:c.8215G>A	NP_002102.4:p.Ala2739Thr	0.944	1	0	1	-	-	-	1	0	1	4.22E-03	3.99E-01	-	-	-	-	-	-	-
European	HTT	4:3241683 G>A	NM_002111.8:c.9326G>A	NP_002102.4:p.Arg3109His	32	-	-	-	-	-	-	1	0	1	1.38E-17	-	-	-	-	-	-	-	-
European	LAMP1	13:113960866 T>C	NM_005561.4:c.128T>C	NP_005552.3:p.Met43Thr	21.3	-	-	-	-	-	-	1	0	1	8.41E-21	-	-	-	-	-	-	-	-
Both	LAMP1	13:113973786 A>T	NM_005561.4:c.565A>T	NP_005552.3:p.Thr189Ser	15.07	-	-	-	1	0	1	1	0	1	8.29E-06	-	8.97E-01	1	0	1	4.12E-02	-	1.57E-01
European	LAMP1	13:113974752 G>T	NM_005561.4:c.843G>T	NP_005552.3:p.Glu281Asp	8.8	-	-	-	-	-	-	1	0	1	9.48E-21	-	-	-	-	-	-	-	-
European	LAMP1	13:113974753 G>T	NM_005561.4:c.844G>T	NP_005552.3:p.Gly282Cys	15.21	-	-	-	-	-	-	1	0	1	9.45E-21	-	-	-	-	-	-	-	-
Both	LAMP1	13:113975768 T>C	NM_005561.4:c.926T>C	NP_005552.3:p.Ile309Thr	0.388	2	0	2	2	0	2	2	0	2	7.91E-03	2.33E-01	8.55E-01	2	0	2	3.38E-02	2.92E-04	4.53E-02
European	LAMP3	3:182870252 C>T	NM_014398.4:c.799G>A	NP_055213.2:p.Ala267Thr	10.76	-	-	-	-	-	-	1	0	1	5.36E-10	-	-	-	-	-	-	-	-
European	LAPTM4B	8:98817669 A>C	NM_018407.6:c.188A>C	NP_060877.4:p.Asp63Ala	26.5	-	-	-	-	-	-	1	0	1	9.60E-04	-	-	-	-	-	-	-	-
European	LAPTM4B	8:98837319 C>T	NM_018407.6:c.541C>T	NP_060877.4:p.Arg181Ter	40	-	-	-	-	-	-	1	0	1	5.71E-11	-	-	-	-	-	-	-	-
European	LRRK2	12:40631860 G>C	NM_198578.4:c.526G>C	NP_940980.4:p.Val176Leu	12.73	-	-	-	-	-	-	1	0	1	5.89E-11	-	-	-	-	-	-	-	-
European	LRRK2	12:40634393 A>G	NM_198578.4:c.680A>G	NP_940980.4:p.His227Arg	13.77	-	-	-	-	-	-	1	0	1	2.17E-40	-	-	-	-	-	-	-	-
European	LRRK2	12:40671989 A>G	NM_198578.4:c.2167A>G	NP_940980.4:p.Ile723Val	12.73	77	2	7.90E+01	24	1	25	53	3	56	3.86E-02	4.97E-07	4.74E-06	-	-	-	-	-	-
European	LRRK2	12:40734202 G>A	NM_198578.4:c.6055G>A	NP_940980.4:p.Gly2019Ser	31	62	1	63	-	-	-	4	0	4	3.50E-19	3.17E-04	-	-	-	-	-	-	-
European	LRRK2	12:40740660 G>C	NM_198578.4:c.6215G>C	NP_940980.4:p.Arg2072Thr	26.8	-	-	-	-	-	-	1	0	1	-	-	-	-	-	-	-	-	-
European	MAPT	17:44039792 C>T	NM_001123066.4:c.89C>T	NP_001116538.2:p.Thr30Ile	21.7	-	-	-	-	-	-	1	0	1	9.77E-04	-	-	-	-	-	-	-	-
European	MAPT	17:44039819 C>T	NM_001123066.4:c.116C>T	NP_001116538.2:p.Thr39Met	7.412	-	-	-	-	-	-	1	0	1	8.80E-21	-	-	-	-	-	-	-	-
European	MAPT	17:44060598 C>G	NM_001123066.4:c.428C>G	NP_001116538.2:p.Pro143Arg	8.283	-	-	-	-	-	-	1	0	1	-	-	-	-	-	-	-	-	-
European	MAPT	17:44060775 C>T	NM_001123066.4:c.605C>T	NP_001116538.2:p.Pro202Leu	11.57	162	21	1.83E+02	65	8	73	112	2	114	4.41E-02	1.35E-07	4.47E-05	-	-	-	-	-	-
European	MAPT	17:44061048 A>G	NM_001123066.4:c.878A>G	NP_001116538.2:p.Lys293Arg	10.62	-	-	-	-	-	-	1	0	1	2.29E-03	-	-	-	-	-	-	-	-
European	MAPT	17:44067307 A>G	NM_001123066.4:c.1246A>G	NP_001116538.2:p.Thr416Ala	19.75	-	-	-	-	-	-	1	0	1	4.78E-10	-	-	-	-	-	-	-	-
European	MCCC1	3:182733255 C>T	NM_020166.5:c.2149G>A	NP_064551.3:p.Glu717Lys	23	1	0	1	-	-	-	1	0	1	1.89E-05	3.99E-01	-	-	-	-	-	-	-
European	MCCC1	3:182733287 T>C	NM_020166.5:c.2117A>G	NP_064551.3:p.Asn706Ser	16.24	-	-	-	-	-	-	1	0	1	-	-	-	-	-	-	-	-	-
European	MCCC1	3:182740306 A>G	NM_020166.5:c.1768T>C	NP_064551.3:p.Tyr590His	7.715	-	-	-	-	-	-	1	0	1	1.50E-04	-	-	-	-	-	-	-	-
European	MCCC1	3:182756852 C>A	NM_020166.5:c.1339G>T	NP_064551.3:p.Ala447Ser	25.1	-	-	-	-	-	-	1	0	1	6.30E-05	-	-	-	-	-	-	-	-
European	MCOLN1	19:7591731 G>A	NM_020533.3:c.490G>A	NP_065394.1:p.Ala164Thr	17.3	-	-	-	-	-	-	1	0	1	2.17E-03	-	-	-	-	-	-	-	-
European	MCOLN1	19:7591764 G>A	NM_020533.3:c.523G>A	NP_065394.1:p.Val175Met	22.7	-	-	-	-	-	-	1	0	1	1.24E-06	-	-	-	-	-	-	-	-
European	MCOLN1	19:7592763 A>C	NM_020533.3:c.694A>C	NP_065394.1:p.Thr232Pro	27.7	-	-	-	-	-	-	1	0	1	5.63E-09	-	-	-	-	-	-	-	-

Population	Gene Name	Genomic	HGVS c.	HGVS p.	CADD Score	#Het PPMI PD	#Hom PPMI PD	#Subjects PPMI PD	#Het PPMI HC	#Hom PPMI HC	#Subjects PPMI HC	European #Het PEG	European #HomoVar PEG	European #Subjects PEG	European PEG v gnomAD P-value	European PEG v PPMI PD P-value	European PEG vs.PPMI HC P-value	Hispanic #Het PEG	Hispanic #HomoVar PEG	Hispanic #Subjects PEG	Hispanic PEG v gnomAD P-value	Hispanic PEG v PPMI PD P-value	Hispanic PEG vs.PPMI HC P-value
European	*NPC1*	18:21113384 A>G	NM_000271.5:c.3689T>C	NP_000262.2:p.Leu1230Ser	26.3	-	-	-	-	-	-	1	0	1	1.12E-06	-	-	-	-	-	-	-	-
European	*NPC1*	18:21119822 A>C	NM_000271.5:c.2748T>G	NP_000262.2:p.Asn916Lys	17.94	-	-	-	-	-	-	1	0	1	2.73E-14	-	-	-	-	-	-	-	-
European	*NPC1*	18:21119949 T>A	NM_000271.5:c.2621A>T	NP_000262.2:p.Asp874Val	25.2	-	-	-	-	-	-	1	0	1	2.11E-04	-	-	-	-	-	-	-	-
European	*NPC1*	18:21121284 T>C	NM_000271.5:c.2359A>G	NP_000262.2:p.Ile787Val	16.66	-	-	-	-	-	-	1	0	1	2.63E-02	-	-	-	-	-	-	-	-
European	*NPC1*	18:21141402 T>C	NM_000271.5:c.553A>G	NP_000262.2:p.Asn185Asp	22.9	-	-	-	-	-	-	1	0	1	2.99E-03	-	-	-	-	-	-	-	-
European	*PIK3R1*	5:67593425 G>A	NM_181523.3:c.2171G>A	NP_852664.1:p.Arg724Gln	26.5	-	-	-	-	-	-	1	0	1	3.57E-40	-	-	-	-	-	-	-	-
European	*PINK1*	1:20966479 C>T	NM_032409.3:c.770C>T	NP_115785.1:p.Thr257Ile	12.7	-	-	-	-	-	-	1	0	1	6.46E-04	-	-	-	-	-	-	-	-
European	*PINK1*	1:20971008 C>G	NM_032409.3:c.802C>G	NP_115785.1:p.Leu268Val	22.8	-	-	-	-	-	-	1	0	1	1.01E-03	-	-	-	-	-	-	-	-
European	*PINK1*	1:20972055 A>G	NM_032409.3:c.962A>G	NP_115785.1:p.Tyr321Cys	29	-	-	-	-	-	-	1	0	1	7.31E-21	-	-	-	-	-	-	-	-
European	*PINK1*	1:20977011 G>A	NM_032409.3:c.1573G>A	NP_115785.1:p.Asp525Asn	23.1	-	-	-	-	-	-	1	0	1	9.57E-04	-	-	-	-	-	-	-	-
European	*PRKCD*	3:53226254 C>A	NM_212539.2:c.2003C>A	NP_997704.1:p.Pro668His	25.8	-	-	-	-	-	-	1	0	1	1.49E-40	-	-	-	-	-	-	-	-
European	*PRKN*	6:161781200 C>T	NM_004562.3:c.1205G>A	NP_004553.2:p.Arg402His	25.9	-	-	-	-	-	-	1	0	1	2.72E-14	-	-	-	-	-	-	-	-
European	*PRKN*	6:161781201 G>A	NM_004562.3:c.1204C>T	NP_004553.2:p.Arg402Cys	24.2	1	0	1	1	0	1	5	0	5	3.17E-03	7.63E-04	6.18E-02	-	-	-	-	-	-
Both	*PRKN*	6:161807855 C>G	NM_004562.3:c.1138G>C	NP_004553.2:p.Val380Leu	11.19	132	7	139	-	-	-	103	11	114	1.98E-02	1.38E-18	-	21	1	22	4.43E-02	3.62E-05	-
European	*PRKN*	6:162394341 C>T	NM_004562.3:c.727G>A	NP_004553.2:p.Asp243Asn	23.8	-	-	-	-	-	-	1	0	1	1.51E-03	-	-	-	-	-	-	-	-
European	*PRKN*	6:162683589 C>T	NM_004562.3:c.380G>A	NP_004553.2:p.Ser127Asn	2.313	-	-	-	-	-	-	1	0	1	-	-	-	-	-	-	-	-	-
European	*PRKN*	6:162683713 C>A	NM_004562.3:c.256G>T	NP_004553.2:p.Asp86Tyr	12.03	-	-	-	-	-	-	1	0	1	-	-	-	-	-	-	-	-	-
European	*PRKN*	6:162864403 G>A	NM_004562.3:c.110C>T	NP_004553.2:p.Pro37Leu	25	2	0	2	-	-	-	1	0	1	4.59E-02	7.18E-01	-	-	-	-	-	-	-
European	*PSAP*	10:73587784 C>A	NM_002778.4:c.707G>T	NP_002769.1:p.Gly236Val	23.7	-	-	-	-	-	-	1	0	1	1.23E-07	-	-	-	-	-	-	-	-
European	*PSAP*	10:73587879 C>T	NM_002778.4:c.612G>A	NP_002769.1:p.Met204Ile	22.9	-	-	-	-	-	-	1	0	1	5.59E-09	-	-	-	-	-	-	-	-
European	*PSAP*	10:73587913 T>C	NM_002778.4:c.578A>G	NP_002769.1:p.Asp193Gly	1.461	-	-	-	-	-	-	2	0	2	2.64E-03	-	-	-	-	-	-	-	-
European	*RAB1A*	2:65315643 C>T	NM_004161.5:c.602G>A	NP_004152.1:p.Gly201Asp	21.3	-	-	-	-	-	-	1	0	1	6.79E-14	-	-	-	-	-	-	-	-
European	*RB1CC1*	8:53548616 T>C	NM_014781.5:c.4351A>G	NP_055596.3:p.Met1451Val	15.24	-	-	-	2	0	2	9	0	9	4.57E-02	-	1.50E-02	-	-	-	-	-	-
European	*RB1CC1*	8:53570467 G>A	NM_014781.5:c.1922C>T	NP_055596.3:p.Ala641Val	13.74	-	-	-	-	-	-	1	0	1	2.29E-04	-	-	-	-	-	-	-	-
European	*SMPD1*	11:6411935_6411946delTGCTGGCGCTGG	NM_001007593.3:c.107_118delTGCTGGCGCTGG	NP_001007594.2:p.Val36_Leu39del	14.95	-	-	-	-	-	-	1	0	1	1.29E-13	-	-	-	-	-	-	-	-
European	*SMPD1*	11:6411935_6411958del24	NM_001007593.3:c.107_130del	NP_001007594.2:p.Val36_Leu43del	16.58	-	-	-	-	-	-	1	1	2	1.21E-02	-	-	-	-	-	-	-	-
European	*SMPD1*	11:6413115 A>G	NM_001007593.3:c.817A>G	NP_001007594.2:p.Met273Val	18.63	-	-	-	-	-	-	1	0	1	1.29E-07	-	-	-	-	-	-	-	-
European	*SMPD1*	11:6413196 G>A	NM_001007593.3:c.898G>A	NP_001007594.2:p.Val300Ile	0.64	1	0	1	1	0	1	1	0	1	1.55E-08	3.99E-01	8.97E-01	-	-	-	-	-	-
Both	*SMPD1*	11:6415245 C>T	NM_001007593.3:c.1457C>T	NP_001007594.2:p.Ala486Val	21	5	0	5	1	0	1	7	0	7	2.10E-02	6.06E-03	1.66E-02	1	0	1	9.33E-04	2.90E-01	1.57E-01
European	*SMPD1*	11:6415434 G>T	NM_001007593.3:c.1490G>T	NP_001007594.2:p.Arg497Leu	26.6	-	-	-	1	0	1	1	0	1	1.96E-07	-	8.97E-01	-	-	-	-	-	-
Both	*SMPD1*	11:6415566 G>A	NM_001007593.3:c.1622G>A	NP_001007594.2:p.Arg541Gln	23.1	-	-	-	-	-	-	1	0	1	1.91E-07	-	-	1	0	1	2.84E-03	-	-
European	*SNCA*	4:90756788 C>A	NM_000345.4:c.31G>T	NP_000336.1:p.Ala11Ser	26.9	-	-	-	-	-	-	1	0	1	6.93E-21	-	-	-	-	-	-	-	-
European	*STK39*	2:168931737 C>T	NM_013233.3:c.1094G>A	NP_037365.2:p.Arg365Lys	22.6	-	-	-	-	-	-	1	0	1	6.34E-11	-	-	-	-	-	-	-	-
European	*TFEB*	6:41652437 G>A	NM_007162.3:c.1331C>T	NP_009093.1:p.Pro444Leu	24.4	-	-	-	-	-	-	1	0	1	2.34E-40	-	-	-	-	-	-	-	-
European	*TFEB*	6:41652531 A>T	NM_007162.3:c.1237T>A	NP_009093.1:p.Tyr413Asn	24.5	-	-	-	-	-	-	1	0	1	-	-	-	-	-	-	-	-	-
European	*TFEB*	6:41655688 T>C	NM_007162.3:c.628A>G	NP_009093.1:p.Ser210Gly	23.6	-	-	-	-	-	-	1	0	1	-	-	-	-	-	-	-	-	-
European	*TFEB*	6:41658508 C>T	NM_007162.3:c.361G>A	NP_009093.1:p.Ala121Thr	17.87	-	-	-	-	-	-	1	0	1	1.40E-06	-	-	-	-	-	-	-	-
European	*TMBIM6*	12:50146842 A>G	NM_001098576.2:c.317A>G	NP_001092046.1:p.His106Arg	25.3	-	-	-	-	-	-	1	0	1	7.68E-42	-	-	-	-	-	-	-	-
European	*TP53*	17:7578280 G>C	NM_001276761.3:c.452C>G	NP_001263690.1:p.Pro151Arg	23.6	-	-	-	-	-	-	1	0	1	-	-	-	-	-	-	-	-	-
European	*TP53INP2*	20:33298008 A>T	NM_021202.3:c.560A>T	NP_067025.1:p.Lys187Met	29.9	-	-	-	-	-	-	1	0	1	-	-	-	-	-	-	-	-	-
European	*TSC1*	9:135772014 C>T	NM_001162427.2:c.2950G>A	NP_001155899.1:p.Gly984Ser	16	1	0	1	-	-	-	5	0	5	7.49E-04	7.63E-04	-	-	-	-	-	-	-
European	*TSG101*	11:18531089 T>C	NM_006292.4:c.481A>G	NP_006283.1:p.Thr161Ala	18.23	-	-	-	-	-	-	1	0	1	1.80E-23	-	-	-	-	-	-	-	-
European	*ULK1*	12:132399651 G>A	NM_003565.4:c.1397G>A	NP_003556.2:p.Gly466Asp	16.49	-	-	-	-	-	-	1	0	1	2.45E-12	-	-	-	-	-	-	-	-
European	*ULK1*	12:132401517 C>T	NM_003565.4:c.2092C>T	NP_003556.2:p.Leu698Phe	24.8	1	0	1	-	-	-	1	0	1	8.20E-09	3.99E-01	-	-	-	-	-	-	-
Both	*ULK1*	12:132402057 G>A	NM_003565.4:c.2284G>A	NP_003556.2:p.Gly762Arg	25.6	-	-	-	-	-	-	2	0	2	3.78E-03	-	-	1	0	1	2.04E-03	-	-
European	*ULK1*	12:132403899 A>C	NM_003565.4:c.2654A>C	NP_003556.2:p.Asp885Ala	24.9	-	-	-	-	-	-	7	0	7	-	-	-	-	-	-	-	-	-
European	*ULK1*	12:132404089 C>G	NM_003565.4:c.2757C>G	NP_003556.2:p.Asp919Glu	12.68	-	-	-	-	-	-	1	0	1	4.02E-14	-	-	-	-	-	-	-	-
European	*ULK1*	12:132404102 G>A	NM_003565.4:c.2770G>A	NP_003556.2:p.Gly924Ser	28.2	-	-	-	-	-	-	1	0	1	7.55E-11	-	-	-	-	-	-	-	-
European	*USP33*	1:78180445 T>C	NM_015017.5:c.2162A>G	NP_055832.3:p.Lys721Arg	16.17	-	-	-	-	-	-	1	0	1	7.99E-21	-	-	-	-	-	-	-	-
European	*VCP*	9:35060820 C>T	NM_007126.5:c.1460G>A	NP_009057.1:p.Arg487His	26.7	-	-	-	-	-	-	1	0	1	4.58E-16	-	-	-	-	-	-	-	-
European	*VPS39*	15:42457275 G>A	NM_015289.5:c.1816C>T	NP_056104.2:p.Arg606Trp	23	-	-	-	-	-	-	1	0	1	1.13E-06	-	-	-	-	-	-	-	-

Supplementary Table 5

Population	Gene Name	Genomic	HGVS c.	HGVS p.	CADD Score	European #Het PEG	European #HomoVar PEG	European #Subjects PEG	Allele Count European (non-Finnish)	Allele Number European (non-Finnish)	Allele Frequency European (non-Finnish)	European PEG v gnomAD P-value	European PEG v PPMI PD P-value	European PEG vs.PPMI HC P-value	European Disease x Pesticide Score	Hispanic #Het PEG	Hispanic #HomoVar PEG	Hispanic #Subjects PEG	Allele Count Latino/Admixed American	Allele Number Latino/Admixed American	Allele Frequency Latino/Admixed American	Hispanic PEG v gnomAD P-value	Hispanic PEG v PPMI PD P-value	Hispanic PEG vs.PPMI HC P-value	Hispanic Disease x Pesticide Score
Hispanic	*FBXO7*	22:32894494 G>C	NM_012179.4:c.1546G>C	NP_036311.3:p.Asp516His	24.7	-	-	-	246	128760	1.91E-03	-	-	-	-	1	0	1	16	35386	4.52E-04	1.76E-04	1.01E-02	3.56E-01	0.230029499
Hispanic	*CTSD*	11:1778666 T>C	NM_001909.5:c.592A>G	NP_001900.1:p.Ile198Val	19.43	-	-	-	0	128524	0.00E+00	-	-	-	-	1	0	1	4	35398	1.13E-04	5.40E-13	-	-	0.229657881
Hispanic	*GBA1*	1:155209752 G>A	NM_001005742.3:c.232C>T	NP_001005742.1:p.Arg78Cys	23.8	-	-	-	18	129116	1.39E-04	-	-	-	-	1	0	1	1	35410	2.82E-05	8.43E-31	5.22E-02	-	0.110114186
Hispanic	*GAK*	4:877843 T>C	NM_005255.4:c.1237A>G	NP_005246.2:p.Ile413Val	19.63	-	-	-	229	128834	1.78E-03	-	-	-	-	1	0	1	26	35412	7.34E-04	4.40E-03	-	1.57E-01	0.076492955
Hispanic	*PINK1*	1:20975021 G>A	NM_032409.3:c.1147G>A	NP_115785.1:p.Ala383Thr	13.23	-	-	-	31	129022	2.40E-04	-	-	-	-	1	0	1	15	35424	4.23E-04	1.10E-04	-	-	0.076492955
European	*EP300*	22:41574951 C>G	NM_001429.4:c.7236C>G	NP_001420.2:p.Asp2412Glu	22.3	1	0	1	1	113282	8.83E-06	8.40E-21	-	-	0.538403837	-	-	-	0	34560	0.00E+00	-	-	-	-
European	*EP300*	22:41521890 A>G	NM_001429.4:c.752A>G	NP_001420.2:p.Asn251Ser	9.748	1	0	1	30	129164	2.32E-04	2.98E-02	-	-	0.45615682	-	-	-	5	35426	1.41E-04	-	-	-	-
European	*HDAC6*	X:48681339 G>A	NM_006044.4:c.2530G>A	NP_006035.2:p.Gly844Arg	11.87	1	0	1	1	83628	1.20E-05	7.30E-22	2.49E-01	-	0.45615682	-	-	-	6	25378	2.36E-04	-	-	-	-
European	*HTT*	4:3134324 G>A	NM_002111.8:c.2272G>A	NP_002102.4:p.Asp758Asn	22	1	0	1	0	113214	0.00E+00	2.30E-40	-	-	0.235120761	-	-	-	0	34522	0.00E+00	-	-	-	-
European	*HTT*	4:3241683 G>A	NM_002111.8:c.9326G>A	NP_002102.4:p.Arg3109His	32	1	0	1	1	94558	1.06E-05	1.38E-17	-	-	0.235120761	-	-	-	0	31006	0.00E+00	-	-	-	-
European	*ACP2*	11:47264339 G>A	NM_001610.4:c.1073C>T	NP_001601.1:p.Thr358Ile	22.8	1	0	1	7	129004	5.43E-05	1.26E-06	-	-	0.228617856	-	-	-	0	35438	0.00E+00	-	-	-	-
European	*PRKN*	6:162864403 G>A	NM_004562.3:c.110C>T	NP_004553.2:p.Pro37Leu	25	1	0	1	34	129090	2.63E-04	4.59E-02	7.18E-01	-	0.228617856	-	-	-	6	35440	1.69E-04	-	-	-	-
European	*FBXO7*	22:32894401 G>A	NM_012179.4:c.1453G>A	NP_036311.3:p.Val485Ile	0.009	2	0	2	86	129168	6.66E-04	1.71E-02	8.80E-02	-	0.184037074	-	-	-	10	35434	2.82E-04	-	-	-	-
Both	*LAMP1*	13:113973786 A>T	NM_005561.4:c.565A>T	NP_005552.3:p.Thr189Ser	15.07	1	0	1	8	124476	6.43E-05	8.29E-06	-	8.97E-01	0.178481344	1	0	1	43	34600	1.24E-03	4.12E-02	-	1.57E-01	0.090331568
European	*APP*	21:27269956 C>T	NM_000484.4:c.1993G>A	NP_000475.1:p.Glu665Lys	25.7	1	0	1	1	113680	8.80E-06	7.18E-21	-	-	0.16616192	-	-	-	0	34592	0.00E+00	-	-	-	-
European	*HIP1R*	12:123342671 G>A	NM_003959.3:c.1838G>A	NP_003950.1:p.Arg613Gln	9.163	1	0	1	1	124984	8.00E-06	4.44E-23	-	-	0.160292531	-	-	-	1	34676	2.88E-05	-	-	-	-
European	*MAPT*	17:44039792 C>T	NM_001123066.4:c.89C>T	NP_001116538.2:p.Thr30Ile	21.7	1	0	1	13	113424	1.15E-04	9.77E-04	-	-	0.15956442	-	-	-	0	34530	0.00E+00	-	-	-	-
European	*MAPT*	17:44061048 A>G	NM_001123066.4:c.878A>G	NP_001116538.2:p.Lys293Arg	10.62	1	0	1	15	113588	1.32E-04	2.29E-03	-	-	0.15956442	-	-	-	0	34576	0.00E+00	-	-	-	-
European	*ATG4C*	1:63329808 C>T	NM_032852.4:c.1355C>T	NP_116241.2:p.Thr452Met	16.36	1	0	1	4	125974	3.18E-05	7.90E-10	-	-	0.129933969	-	-	-	17	32250	5.27E-04	-	-	-	-
European	*GNPTAB*	12:102147227 C>A	NM_024312.5:c.3525G>T	NP_077288.2:p.Met1175Ile	25.8	1	0	1	2	113678	1.76E-05	2.78E-14	-	-	0.111059739	-	-	-	0	34582	0.00E+00	-	-	-	-
European	*HIP1R*	12:123341703 C>T	NM_003959.3:c.1756C>T	NP_003950.1:p.Arg586Trp	24.1	1	0	1	3	72262	4.15E-05	2.32E-07	3.99E-01	-	0.08775281	-	-	-	0	25514	0.00E+00	-	-	-	-
European	*SMPD1*	11:6415434 G>T	NM_001007593.3:c.1490G>T	NP_001007594.2:p.Arg497Leu	26.6	1	0	1	6	128826	4.66E-05	1.96E-07	-	8.97E-01	0.08775281	-	-	-	0	35428	0.00E+00	-	-	-	-
European	*GALC*	14:88452845delT	NM_000153.4:c.430delA	NP_000144.2:p.Ile144Leufs*27	32	1	0	1	4	113110	3.54E-05	6.21E-09	-	-	0.082415342	-	-	-	0	34482	0.00E+00	-	-	-	-
European	*BAG6*	6:31614212 C>T	NM_080703.3:c.877G>A	NP_542434.1:p.Glu293Lys	24.2	1	0	1	1	15420	6.49E-05	8.78E-04	-	-	0.078713615	-	-	-	0	848	0.00E+00	-	-	-	-
European	*BLOC1S1*	12:56110773 G>A	NM_001487.4:c.202G>A	NP_001478.2:p.Asp68Asn	32	3	0	3	191	124230	1.54E-03	4.36E-02	-	-	0.073941695	-	-	-	20	31556	6.34E-04	-	-	-	-
European	*MCCC1*	3:182733255 C>T	NM_020166.5:c.2149G>A	NP_064551.3:p.Glu717Lys	23	1	0	1	9	128372	7.01E-05	1.89E-05	3.99E-01	-	0.073497168	-	-	-	24	35440	6.77E-04	-	-	-	-
European	*HDAC6*	X:48674577 T>C	NM_006044.4:c.1523T>C	NP_006035.2:p.Leu508Ser	1.232	1	0	1	9	80981	1.11E-04	5.34E-05	-	-	0.061941494	-	-	-	5	27408	1.82E-04	-	-	-	-
European	*NPC1*	18:21121284 T>C	NM_000271.5:c.2359A>G	NP_000262.2:p.Ile787Val	16.66	1	0	1	29	129186	2.24E-04	2.63E-02	-	-	0.054704559	-	-	-	0	35438	0.00E+00	-	-	-	-
European	*ACMSD*	2:135621115 A>G	NM_138326.3:c.400A>G	NP_612199.2:p.Lys134Glu	21.3	1	0	1	31	129080	2.40E-04	3.36E-02	3.99E-01	-	0.048657095	-	-	-	25	35430	7.06E-04	-	-	-	-
European	*BAG6*	6:31612806 G>A	NM_080703.3:c.1286C>T	NP_542434.1:p.Pro429Leu	23.2	0	1	1	2	124468	1.61E-05	7.72E-88	-	-	0.047010377	-	-	-	0	35018	0.00E+00	-	-	-	-
European	*GAK*	4:870891 C>T	NM_005255.4:c.1961G>A	NP_005246.2:p.Arg654Gln	26.2	1	0	1	31	128204	2.42E-04	3.44E-02	-	-	0.047010377	-	-	-	3	35340	8.49E-05	-	-	-	-
European	*MCOLN1*	19:7591731 G>A	NM_020533.3:c.490G>A	NP_065394.1:p.Ala164Thr	17.3	1	0	1	17	129162	1.32E-04	2.17E-03	-	-	0.047010377	-	-	-	2	35440	5.64E-05	-	-	-	-
European	*LAMP1*	13:113975768 T>C	NM_005561.4:c.926T>C	NP_005552.3:p.Ile309Thr	0.388	2	0	2	74	128716	5.75E-04	7.91E-03	2.33E-01	8.55E-01	0.046436454	-	-	-	2071	35366	5.86E-02	-	-	-	-
European	*TSC1*	9:135772014 C>T	NM_001162427.2:c.2950G>A	NP_001155899.1:p.Gly984Ser	16	5	0	5	246	128108	1.92E-03	7.49E-04	7.63E-04	-	0.045819774	-	-	-	62	35380	1.75E-03	-	-	-	-
European	*LAMP3*	3:182870252 C>T	NM_014398.4:c.799G>A	NP_055213.2:p.Ala267Thr	10.76	1	0	1	4	128402	3.12E-05	5.36E-10	-	-	0.036017553	-	-	-	0	35066	0.00E+00	-	-	-	-
European	*GALC*	14:88452941 T>C	NM_000153.4:c.334A>G	NP_000144.2:p.Thr112Ala	24.4	7	0	7	516	128292	4.02E-03	6.04E-03	6.06E-03	1.18E-01	0.029211969	-	-	-	34	35290	9.63E-04	-	-	-	-

Supplementary File 1

chr6	31610008	31610235	chr6:31610008:31610235:216023294	227	+
chr6	31607836	31608061	chr6:31607836:31608061:216023295	225	-
chr6	31608164	31608389	chr6:31608164:31608389:216023296	225	-
chr6	31608504	31608729	chr6:31608504:31608729:216023297	225	-
chr6	31608836	31609062	chr6:31608836:31609062:216023298	226	-
chr6	31609178	31609403	chr6:31609178:31609403:216023299	225	-
chr6	31609484	31609709	chr6:31609484:31609709:216023300	225	-
chr6	31609830	31610058	chr6:31609830:31610058:216023301	228	-
chr6	31611837	31612082	chr6:31611837:31612082:216023304	245	+
chr6	31611625	31611890	chr6:31611625:31611890:216023305	265	-
chr6	31615512	31615751	chr6:31615512:31615751:216023310	239	+
chr6	31615330	31615560	chr6:31615330:31615560:216023311	230	-
chr6	31612274	31612529	chr6:31612274:31612529:216023351	255	-
chr19	49458888	49459152	chr19:49458888:49459152:216023566	264	+
chr19	49459316	49459569	chr19:49459316:49459569:216023567	253	+
chr19	49458670	49458942	chr19:49458670:49458942:216023568	272	-
chr19	49459100	49459372	chr19:49459100:49459372:216023569	272	-
chr19	49459514	49459769	chr19:49459514:49459769:216023570	255	-
chr12	123285803	123286044	chr12:123285803:123286044:216023392	241	+
chr12	123286209	123286468	chr12:123286209:123286468:216023393	259	+
chr12	123285621	123285858	chr12:123285621:123285858:216023394	237	-
chr12	123285989	123286263	chr12:123285989:123286263:216023395	274	-
chr12	56110689	56110961	chr12:56110689:56110961:216023356	272	-
chr12	123265679	123265926	chr12:123265679:123265926:216023390	247	-
chr12	123276519	123276746	chr12:123276519:123276746:216023388	227	-
chr12	102155054	102155323	chr12:102155054:102155323:216023645	269	+
chr12	102155472	102155715	chr12:102155472:102155715:216023646	243	+
chr12	102154840	102155108	chr12:102154840:102155108:216023647	268	-
chr12	102155268	102155527	chr12:102155268:102155527:216023648	259	-
chr12	102174111	102174381	chr12:102174111:102174381:216023653	270	+
chr12	102173895	102174166	chr12:102173895:102174166:216023654	271	-
chr12	102174325	102174552	chr12:102174325:102174552:216023655	227	-
chr12	50135892	50136161	chr12:50135892:50136161:216024069	269	-
chr12	50149368	50149593	chr12:50149368:50149593:216024071	225	-
chr12	50155457	50155718	chr12:50155457:50155718:216024072	261	-
chr12	50152158	50152387	chr12:50152158:50152387:216024052	229	+
chr12	50151968	50152209	chr12:50151968:50152209:216024053	241	-
chr12	50152332	50152601	chr12:50152332:50152601:216024054	269	-
chr12	132398262	132398501	chr12:132398262:132398501:216023578	239	-
chr1	203830760	203830995	chr1:203830760:203830995:216024032	235	-
chr1	63286758	63286991	chr1:63286758:63286991:216023171	233	-
chr1	63294789	63295033	chr1:63294789:63295033:216023172	244	+
chr1	63294583	63294844	chr1:63294583:63294844:216023173	261	-
chr1	63300415	63300676	chr1:63300415:63300676:216023175	261	-
chr1	63307169	63307420	chr1:63307169:63307420:216023176	251	+
chr1	63306999	63307224	chr1:63306999:63307224:216023177	225	-
chr1	155208086	155208357	chr1:155208086:155208357:216023583	271	+

chr1	155207868	155208140	chr1:155207868:155208140:216023584	272	-
chr1	155208302	155208531	chr1:155208302:155208531:216023585	229	-
chr1	17320274	17320501	chr1:17320274:17320501:216023205	227	+
chr1	17320098	17320324	chr1:17320098:17320324:216023206	226	-
chr1	17323491	17323734	chr1:17323491:17323734:216023207	243	-
chr1	17314805	17315060	chr1:17314805:17315060:216023214	255	+
chr1	17314591	17314851	chr1:17314591:17314851:216023215	260	-
chr1	17318381	17318630	chr1:17318381:17318630:216023219	249	+
chr1	17318797	17319067	chr1:17318797:17319067:216023220	270	+
chr1	17318187	17318434	chr1:17318187:17318434:216023221	247	-
chr1	17318579	17318852	chr1:17318579:17318852:216023222	273	-
chr1	17319017	17319250	chr1:17319017:17319250:216023223	233	-
chr1	17326696	17326969	chr1:17326696:17326969:216023227	273	+
chr1	17326470	17326742	chr1:17326470:17326742:216023228	272	-
chr1	17326918	17327172	chr1:17326918:17327172:216023229	254	-
chr1	17332009	17332263	chr1:17332009:17332263:216023237	254	+
chr1	17331829	17332058	chr1:17331829:17332058:216023238	229	-
chr1	17332213	17332445	chr1:17332213:17332445:216023239	232	-
chr1	87170547	87170774	chr1:87170547:87170774:216023981	227	-
chr1	155851145	155851386	chr1:155851145:155851386:216024088	241	+
chr1	155850961	155851196	chr1:155850961:155851196:216024089	235	-
chr1	87188185	87188452	chr1:87188185:87188452:216024025	267	-
chr1	87189946	87190207	chr1:87189946:87190207:216023982	261	-
chr1	155837906	155838131	chr1:155837906:155838131:216024056	225	+
chr1	155838276	155838515	chr1:155838276:155838515:216024057	239	+
chr1	155837726	155837962	chr1:155837726:155837962:216024058	236	-
chr1	155838076	155838331	chr1:155838076:155838331:216024059	255	-
chr1	155838462	155838721	chr1:155838462:155838721:216024060	259	-
chr12	102159830	102160101	chr12:102159830:102160101:216023370	271	-
chr12	102147283	102147532	chr12:102147283:102147532:216023742	249	+
chr12	102147111	102147338	chr12:102147111:102147338:216023743	227	-
chr12	102153771	102154046	chr12:102153771:102154046:216023728	275	-
chr12	102179951	102180216	chr12:102179951:102180216:216023730	265	+
chr12	102179733	102180007	chr12:102179733:102180007:216023731	274	-
chr12	102182289	102182528	chr12:102182289:102182528:216023740	239	-
chr12	102183673	102183928	chr12:102183673:102183928:216023745	255	-
chr12	102190418	102190649	chr12:102190418:102190649:216023733	231	-
chr12	123273271	123273546	chr12:123273271:123273546:216023368	275	-
chr12	123290666	123290927	chr12:123290666:123290927:216023387	261	-
chr12	123297785	123298042	chr12:123297785:123298042:216023385	257	-
chr16	731184	731412	chr16:731184:731412:216024012	228	+
chr16	731496	731722	chr16:731496:731722:216024036	226	+
chr16	731814	732039	chr16:731814:732039:216024037	225	+
chr16	732174	732446	chr16:732174:732446:216024038	272	+
chr16	731014	731239	chr16:731014:731239:216024039	225	-
chr16	731348	731575	chr16:731348:731575:216024040	227	-
chr16	731642	731868	chr16:731642:731868:216024041	226	-

chr16	731984	732229	chr16:731984:732229:216024042	245	-
chr16	732392	732621	chr16:732392:732621:216024043	229	-
chr2	168986215	168986466	chr2:168986215:168986466:216023986	251	+
chr2	168986011	168986269	chr2:168986011:168986269:216023987	258	-
chr2	169103828	169104094	chr2:169103828:169104094:216023998	266	+
chr2	169103634	169103902	chr2:169103634:169103902:216023999	268	-
chr2	169018245	169018470	chr2:169018245:169018470:216024019	225	-
chr2	169020217	169020446	chr2:169020217:169020446:216024020	229	-
chr2	168869106	168869361	chr2:168869106:168869361:216024014	255	-
chr2	168811979	168812216	chr2:168811979:168812216:216024031	237	-
chr4	15720480	15720727	chr4:15720480:15720727:216023360	247	-
chr4	15733331	15733588	chr4:15733331:15733588:216023362	257	-
chr4	15707108	15707383	chr4:15707108:15707383:216023358	275	-
chr6	41653800	41654037	chr6:41653800:41654037:216024065	237	-
chr6	41657441	41657700	chr6:41657441:41657700:216024067	259	-
chr6	41703858	41704133	chr6:41703858:41704133:216024068	275	-
chr6	41655641	41655889	chr6:41655641:41655889:216024046	248	+
chr6	41655447	41655688	chr6:41655447:41655688:216024047	241	-
chr17	79651071	79651339	chr17:79651071:79651339:216023747	268	-
chr17	79654009	79654261	chr17:79654009:79654261:216023749	252	-
chr14	102548222	102548450	chr14:102548222:102548450:216023954	228	+
chr14	102548582	102548819	chr14:102548582:102548819:216023955	237	+
chr14	102548012	102548278	chr14:102548012:102548278:216023956	266	-
chr14	102548392	102548637	chr14:102548392:102548637:216023957	245	-
chr14	102551601	102551866	chr14:102551601:102551866:216023893	265	-
chr14	102605557	102605818	chr14:102605557:102605818:216023896	261	-
chr14	102550914	102551151	chr14:102550914:102551151:216023897	237	+
chr14	102551284	102551511	chr14:102551284:102551511:216023898	227	+
chr14	102550694	102550969	chr14:102550694:102550969:216023899	275	-
chr14	102551096	102551339	chr14:102551096:102551339:216023900	243	-
chr11	122928994	122929220	chr11:122928994:122929220:216023876	226	+
chr11	122929320	122929545	chr11:122929320:122929545:216023877	225	+
chr11	122929654	122929879	chr11:122929654:122929879:216023878	225	+
chr11	122929962	122930187	chr11:122929962:122930187:216023879	225	+
chr11	122930238	122930464	chr11:122930238:122930464:216023880	226	+
chr11	122930596	122930845	chr11:122930596:122930845:216023881	249	+
chr11	122928830	122929055	chr11:122928830:122929055:216023882	225	-
chr11	122929158	122929383	chr11:122929158:122929383:216023883	225	-
chr11	122929486	122929711	chr11:122929486:122929711:216023884	225	-
chr11	122929822	122930048	chr11:122929822:122930048:216023885	226	-
chr11	122930100	122930325	chr11:122930100:122930325:216023886	225	-
chr11	122930410	122930651	chr11:122930410:122930651:216023887	241	-
chr11	122930790	122931053	chr11:122930790:122931053:216023888	263	-
chr11	122931471	122931730	chr11:122931471:122931730:216023960	259	+
chr11	122931273	122931526	chr11:122931273:122931526:216023961	253	-
chr12	40702856	40703092	chr12:40702856:40703092:216022864	236	-
chr12	40761404	40761659	chr12:40761404:40761659:216022869	255	-

chr12	40631733	40631976	chr12:40631733:40631976:216022775	243	-
chr12	40651112	40651385	chr12:40651112:40651385:216022782	273	+
chr12	40650932	40651167	chr12:40650932:40651167:216022783	235	-
chr12	40681293	40681538	chr12:40681293:40681538:216022788	245	+
chr12	40681121	40681352	chr12:40681121:40681352:216022789	231	-
chr12	40688597	40688826	chr12:40688597:40688826:216022791	229	-
chr12	40692877	40693116	chr12:40692877:40693116:216022796	239	-
chr12	40696497	40696740	chr12:40696497:40696740:216022797	243	-
chr12	40697888	40698133	chr12:40697888:40698133:216022798	245	+
chr12	40697718	40697944	chr12:40697718:40697944:216022799	226	-
chr12	40699661	40699910	chr12:40699661:40699910:216022800	249	+
chr12	40699487	40699716	chr12:40699487:40699716:216022801	229	-
chr12	40708982	40709253	chr12:40708982:40709253:216022804	271	-
chr12	40722122	40722361	chr12:40722122:40722361:216022810	239	-
chr12	40728935	40729208	chr12:40728935:40729208:216022811	273	+
chr12	40728737	40728991	chr12:40728737:40728991:216022812	254	-
chr12	40734066	40734325	chr12:40734066:40734325:216022813	259	-
chr12	40745481	40745728	chr12:40745481:40745728:216022817	247	+
chr12	40745299	40745537	chr12:40745299:40745537:216022818	238	-
chr12	40753204	40753463	chr12:40753204:40753463:216022822	259	+
chr12	40753022	40753259	chr12:40753022:40753259:216022823	237	-
chr12	40757156	40757425	chr12:40757156:40757425:216022824	269	-
chr12	40758724	40758991	chr12:40758724:40758991:216022825	267	+
chr12	40758516	40758779	chr12:40758516:40758779:216022826	263	-
chr13	113960774	113961047	chr13:113960774:113961047:216022856	273	-
chr13	113964980	113965216	chr13:113964980:113965216:216022857	236	-
chr13	113976573	113976844	chr13:113976573:113976844:216022868	271	-
chr16	87425938	87426175	chr16:87425938:87426175:216022880	237	-
chr16	87436497	87436752	chr16:87436497:87436752:216022883	255	-
chr17	44039676	44039919	chr17:44039676:44039919:216022977	243	-
chr17	44101290	44101563	chr17:44101290:44101563:216022978	273	-
chr17	44049197	44049436	chr17:44049197:44049436:216022954	239	-
chr17	44060700	44060930	chr17:44060700:44060930:216022829	230	+
chr17	44061068	44061317	chr17:44061068:44061317:216022830	249	+
chr17	44060516	44060754	chr17:44060516:44060754:216022831	238	-
chr17	44060882	44061122	chr17:44060882:44061122:216022832	240	-
chr17	44061262	44061521	chr17:44061262:44061521:216022833	259	-
chr17	44064368	44064625	chr17:44064368:44064625:216022956	257	-
chr17	44073913	44074162	chr17:44073913:44074162:216022960	249	+
chr17	44073735	44073963	chr17:44073735:44073963:216022961	228	-
chr17	44068794	44069029	chr17:44068794:44069029:216022834	235	-
chr20	33137752	33137982	chr20:33137752:33137982:216022878	230	-
chr3	182733201	182733454	chr3:182733201:182733454:216022983	253	-
chr3	182817114	182817373	chr3:182817114:182817373:216022965	259	-
chr3	182812315	182812570	chr3:182812315:182812570:216022950	255	-
chr3	182788869	182789143	chr3:182788869:182789143:216022951	274	+
chr3	182788657	182788926	chr3:182788657:182788926:216022952	269	-

chr3	182789089	182789336	chr3:182789089:182789336:216022953	247	-
chr3	182756786	182757053	chr3:182756786:182757053:216022942	267	-
chr3	182763163	182763402	chr3:182763163:182763402:216022944	239	-
chr3	182790104	182790356	chr3:182790104:182790356:216022947	252	-
chr3	182737886	182738147	chr3:182737886:182738147:216022936	261	-
chr3	182740173	182740431	chr3:182740173:182740431:216022937	258	-
chr3	182751737	182751962	chr3:182751737:182751962:216022939	225	-
chr4	3088620	3088889	chr4:3088620:3088889:216022734	269	-
chr4	3105515	3105776	chr4:3105515:3105776:216022836	261	-
chr4	3107056	3107309	chr4:3107056:3107309:216022837	253	-
chr4	3108982	3109244	chr4:3108982:3109244:216022732	262	-
chr4	3117786	3118049	chr4:3117786:3118049:216022838	263	-
chr4	3214263	3214512	chr4:3214263:3214512:216022721	249	-
chr4	3221877	3222134	chr4:3221877:3222134:216022743	257	-
chr4	3224080	3224305	chr4:3224080:3224305:216022723	225	-
chr4	3225679	3225916	chr4:3225679:3225916:216022740	237	-
chr4	3234862	3235137	chr4:3234862:3235137:216022727	275	-
chr4	3237844	3238099	chr4:3237844:3238099:216022750	255	-
chr4	3158759	3158986	chr4:3158759:3158986:216022761	227	-
chr4	3174795	3175034	chr4:3174795:3175034:216022752	239	+
chr4	3174599	3174846	chr4:3174599:3174846:216022753	247	-
chr4	3179027	3179262	chr4:3179027:3179262:216022758	235	-
chr4	3179991	3180244	chr4:3179991:3180244:216022730	253	-
chr4	3182214	3182481	chr4:3182214:3182481:216022725	267	-
chr4	3184051	3184300	chr4:3184051:3184300:216022719	249	-
chr4	3201631	3201876	chr4:3201631:3201876:216022737	245	+
chr4	3201427	3201681	chr4:3201427:3201681:216022738	254	-
chr4	3210596	3210863	chr4:3210596:3210863:216022755	267	+
chr4	3210418	3210649	chr4:3210418:3210649:216022756	231	-
chr4	3076657	3076932	chr4:3076657:3076932:216022746	275	+
chr4	3076453	3076708	chr4:3076453:3076708:216022747	255	-
chr6	32489757	32490032	chr6:32489757:32490032:216022706	275	-
chr6	32485684	32485956	chr6:32485684:32485956:216023799	272	+
chr6	32485468	32485743	chr6:32485468:32485743:216023800	275	-
chr6	32497874	32498121	chr6:32497874:32498121:216023851	247	-
chr9	128002393	128002665	chr9:128002393:128002665:216023974	272	-
chrX	119590566	119590827	chrX:119590566:119590827:216022771	261	+
chrX	119590384	119590621	chrX:119590384:119590621:216022772	237	-
chrX	119580112	119580341	chrX:119580112:119580341:216022765	229	-
chr12	123320052	123320278	chr12:123320052:123320278:216023797	226	-
chr12	123342801	123343071	chr12:123342801:123343071:216023772	270	+
chr12	123342601	123342847	chr12:123342601:123342847:216023773	246	-
chr12	123333157	123333427	chr12:123333157:123333427:216023755	270	+
chr12	123332975	123333203	chr12:123332975:123333203:216023756	228	-
chr12	123333377	123333652	chr12:123333377:123333652:216023757	275	-
chr12	123334394	123334651	chr12:123334394:123334651:216023795	257	-
chr12	123335354	123335625	chr12:123335354:123335625:216023793	271	-

chr13	113964028	113964270	chr13:113964028:113964270:216022715	242	+
chr13	113963828	113964083	chr13:113963828:113964083:216022716	255	-
chr17	79655706	79655966	chr17:79655706:79655966:216023753	260	-
chr9	86293478	86293750	chr9:86293478:86293750:216022702	272	+
chr9	86292556	86292825	chr9:86292556:86292825:216022703	269	-
chr9	86293298	86293533	chr9:86293298:86293533:216022704	235	-
chr9	135778168	135778421	chr9:135778168:135778421:216024121	253	+
chr9	135777964	135778223	chr9:135777964:135778223:216024122	259	-
chr9	135781152	135781413	chr9:135781152:135781413:216023023	261	+
chr9	135781566	135781800	chr9:135781566:135781800:216023024	234	+
chr9	135780940	135781207	chr9:135780940:135781207:216023025	267	-
chr9	135781360	135781620	chr9:135781360:135781620:216023026	260	-
chr9	135786357	135786618	chr9:135786357:135786618:216023027	261	-
chr9	135796696	135796967	chr9:135796696:135796967:216023021	271	-
chr9	135800942	135801217	chr9:135800942:135801217:216023022	275	-
chr9	135802556	135802783	chr9:135802556:135802783:216023029	227	-
chr9	135786812	135787065	chr9:135786812:135787065:216023028	253	-
chr20	33296510	33296738	chr20:33296510:33296738:216023015	228	-
chr6	74331458	74331733	chr6:74331458:74331733:216024090	275	-
chr9	135772715	135772947	chr9:135772715:135772947:216023016	232	+
chr9	135772531	135772769	chr9:135772531:135772769:216023017	238	-
chr9	135772893	135773154	chr9:135772893:135773154:216023018	261	-
chr9	135779010	135779265	chr9:135779010:135779265:216023019	255	-
chr9	135779766	135780007	chr9:135779766:135780007:216023020	241	-
chr9	86278758	86279003	chr9:86278758:86279003:216024259	245	-
chr9	86281233	86281468	chr9:86281233:86281468:216024261	235	-
chr9	86284068	86284338	chr9:86284068:86284338:216024141	270	-
chr9	86297828	86298053	chr9:86297828:86298053:216024262	225	-
chr9	86300887	86301154	chr9:86300887:86301154:216024138	267	-
chr11	18537614	18537845	chr11:18537614:18537845:216024127	231	-
chr11	18548292	18548517	chr11:18548292:18548517:216024135	225	-
chr11	18502068	18502313	chr11:18502068:18502313:216024124	245	-
chr11	18503355	18503584	chr11:18503355:18503584:216024132	229	+
chr11	18503145	18503413	chr11:18503145:18503413:216024133	268	-
chr11	18528276	18528532	chr11:18528276:18528532:216024130	256	-
chr14	68126524	68126766	chr14:68126524:68126766:216024256	242	+
chr14	68126352	68126579	chr14:68126352:68126579:216024257	227	-
chr17	7578327	7578597	chr17:7578327:7578597:216024097	270	+
chr17	7578137	7578379	chr17:7578137:7578379:216024098	242	-
chr17	7573899	7574140	chr17:7573899:7574140:216024104	241	-
chr17	7572902	7573131	chr17:7572902:7573131:216024102	229	-
chr12	132393329	132393568	chr12:132393329:132393568:216024191	239	+
chr12	132393693	132393947	chr12:132393693:132393947:216024192	254	+
chr12	132393151	132393381	chr12:132393151:132393381:216024193	230	-
chr12	132393515	132393745	chr12:132393515:132393745:216024194	230	-
chr12	132399802	132400073	chr12:132399802:132400073:216024199	271	+
chr12	132399588	132399850	chr12:132399588:132399850:216024200	262	-

chr12	50152968	50153215	chr12:50152968:50153215:216024092	247	-
chr12	132403899	132404155	chr12:132403899:132404155:216024205	256	+
chr12	132403721	132403952	chr12:132403721:132403952:216024206	231	-
chr12	132404105	132404337	chr12:132404105:132404337:216024207	232	-
chr12	132379479	132379726	chr12:132379479:132379726:216024182	247	-
chr12	132401470	132401725	chr12:132401470:132401725:216024186	255	-
chr14	68118075	68118310	chr14:68118075:68118310:216024255	235	-
chr14	68129084	68129321	chr14:68129084:68129321:216024258	237	-
chr15	42457166	42457402	chr15:42457166:42457402:216024240	236	-
chr15	42457878	42458123	chr15:42457878:42458123:216024241	245	-
chr15	42458331	42458564	chr15:42458331:42458564:216024242	233	-
chr15	42459564	42459799	chr15:42459564:42459799:216024243	235	-
chr15	42461892	42462117	chr15:42461892:42462117:216024244	225	-
chr15	42476863	42477094	chr15:42476863:42477094:216024246	231	+
chr15	42476687	42476918	chr15:42476687:42476918:216024247	231	-
chr15	42479926	42480179	chr15:42479926:42480179:216024249	253	-
chr15	42481280	42481551	chr15:42481280:42481551:216024250	271	-
chr15	42458895	42459149	chr15:42458895:42459149:216024173	254	+
chr15	42458723	42458949	chr15:42458723:42458949:216024174	226	-
chr15	42459095	42459326	chr15:42459095:42459326:216024175	231	-
chr15	42454048	42454291	chr15:42454048:42454291:216024165	243	+
chr15	42454406	42454637	chr15:42454406:42454637:216024166	231	+
chr15	42453840	42454103	chr15:42453840:42454103:216024167	263	-
chr15	42454236	42454461	chr15:42454236:42454461:216024168	225	-
chr15	42454582	42454817	chr15:42454582:42454817:216024169	235	-
chr9	35059201	35059436	chr9:35059201:35059436:216024149	235	+
chr9	35059585	35059851	chr9:35059585:35059851:216024150	266	+
chr9	35059019	35059256	chr9:35059019:35059256:216024151	237	-
chr9	35059381	35059639	chr9:35059381:35059639:216024152	258	-
chr9	35059795	35060060	chr9:35059795:35060060:216024153	265	-
chr9	35064123	35064380	chr9:35064123:35064380:216024146	257	-
chr1	78183518	78183787	chr1:78183518:78183787:216024214	269	-
chr1	78177381	78177618	chr1:78177381:78177618:216024216	237	-
chr1	78207137	78207378	chr1:78207137:78207378:216024220	241	+
chr1	78206933	78207192	chr1:78206933:78207192:216024221	259	-
chr1	78207323	78207590	chr1:78207323:78207590:216024222	267	-
chr1	78184157	78184422	chr1:78184157:78184422:216024210	265	-
chr1	78178843	78179107	chr1:78178843:78179107:216024211	264	-
chr1	78188984	78189258	chr1:78188984:78189258:216024228	274	-
chr1	78162971	78163218	chr1:78162971:78163218:216024224	247	-
chr1	78163512	78163739	chr1:78163512:78163739:216024225	227	-
chr1	78195597	78195872	chr1:78195597:78195872:216024233	275	+
chr1	78195421	78195650	chr1:78195421:78195650:216024234	229	-
chr1	78196209	78196458	chr1:78196209:78196458:216024235	249	-
chr1	78206490	78206761	chr1:78206490:78206761:216024238	271	-
chr12	132391315	132391576	chr12:132391315:132391576:216023030	261	-
chr12	132404498	132404760	chr12:132404498:132404760:216023033	262	-

chr12	132398871	132399118	chr12:132398871:132399118:216023032	247	-
chr9	35065218	35065449	chr9:35065218:35065449:216024147	231	-
chr9	35066640	35066911	chr9:35066640:35066911:216024148	271	-
chr9	35062158	35062433	chr9:35062158:35062433:216024159	275	+
chr9	35061962	35062208	chr9:35061962:35062208:216024160	246	-
chr6	31614143	31614388	chr6:31614143:31614388:216023352	245	-
chr19	49464749	49465003	chr19:49464749:49465003:216023320	254	-
chr17	40963645	40963888	chr17:40963645:40963888:216023324	243	-
chr17	40965934	40966159	chr17:40965934:40966159:216023325	225	-
chr17	40966514	40966742	chr17:40966514:40966742:216023326	228	-
chr17	40972764	40973009	chr17:40972764:40973009:216023329	245	-
chr17	40970407	40970674	chr17:40970407:40970674:216023240	267	+
chr17	40970837	40971098	chr17:40970837:40971098:216023241	261	+
chr17	40970195	40970461	chr17:40970195:40970461:216023242	266	-
chr17	40970619	40970891	chr17:40970619:40970891:216023243	272	-
chr1	63284844	63285074	chr1:63284844:63285074:216023169	230	+
chr1	63284644	63284899	chr1:63284644:63284899:216023170	255	-
chr1	63329627	63329860	chr1:63329627:63329860:216023178	233	-
chr11	46686367	46686642	chr11:46686367:46686642:216023157	275	-
chr11	46686903	46687177	chr11:46686903:46687177:216023158	274	-
chr11	46689293	46689554	chr11:46689293:46689554:216023159	261	-
chr2	242592901	242593150	chr2:242592901:242593150:216023197	249	-
chr2	242593923	242594189	chr2:242593923:242594189:216023198	266	-
chr2	242607530	242607765	chr2:242607530:242607765:216023202	235	-
chr2	242610111	242610343	chr2:242610111:242610343:216023204	232	-
chr2	242590589	242590862	chr2:242590589:242590862:216023136	273	+
chr2	242590383	242590644	chr2:242590383:242590644:216023137	261	-
chr2	242611564	242611823	chr2:242611564:242611823:216023139	259	-
chr3	112255234	112255493	chr3:112255234:112255493:216023182	259	-
chr3	112260618	112260861	chr3:112260618:112260861:216023183	243	-
chr3	112277191	112277440	chr3:112277191:112277440:216023188	249	-
chr3	112280276	112280510	chr3:112280276:112280510:216023189	234	-
chr3	112267272	112267523	chr3:112267272:112267523:216023185	251	-
chr5	67569739	67569998	chr5:67569739:67569998:216023013	259	-
chr5	67575356	67575617	chr5:67575356:67575617:216023040	261	-
chr5	67588057	67588320	chr5:67588057:67588320:216023011	263	-
chr5	67586525	67586800	chr5:67586525:67586800:216023038	275	-
chr5	67591971	67592206	chr5:67591971:67592206:216023036	235	-
chr5	67576508	67576763	chr5:67576508:67576763:216022999	255	+
chr5	67576308	67576562	chr5:67576308:67576562:216023000	254	-
chr5	67576708	67576949	chr5:67576708:67576949:216023001	241	-
chr5	67591108	67591359	chr5:67591108:67591359:216023007	251	+
chr5	67590934	67591163	chr5:67590934:67591163:216023008	229	-
chr6	161990361	161990594	chr6:161990361:161990594:216022991	233	-
chr6	162622133	162622404	chr6:162622133:162622404:216022995	271	-
chr6	162683729	162683972	chr6:162683729:162683972:216022988	243	+
chr6	162683525	162683783	chr6:162683525:162683783:216022989	258	-

chr6	162864314	162864571	chr6:162864314:162864571:216022997	257	-
chr6	162394302	162394541	chr6:162394302:162394541:216022993	239	-
chr22	51063716	51063951	chr22:51063716:51063951:216023115	235	+
chr22	51064086	51064337	chr22:51064086:51064337:216023116	251	+
chr22	51064496	51064750	chr22:51064496:51064750:216023117	254	+
chr22	51063540	51063765	chr22:51063540:51063765:216023118	225	-
chr22	51063900	51064134	chr22:51063900:51064134:216023119	234	-
chr22	51064286	51064546	chr22:51064286:51064546:216023120	260	-
chr4	907365	907596	chr4:907365:907596:216023618	231	-
chr4	853366	853611	chr4:853366:853611:216023626	245	-
chr4	858882	859154	chr4:858882:859154:216023627	272	-
chr4	860116	860375	chr4:860116:860375:216023508	259	-
chr4	862301	862551	chr4:862301:862551:216023628	250	-
chr4	870850	871089	chr4:870850:871089:216023510	239	-
chr4	875667	875938	chr4:875667:875938:216023513	271	-
chr4	876457	876724	chr4:876457:876724:216023514	267	-
chr4	877079	877332	chr4:877079:877332:216023515	253	-
chr4	884292	884547	chr4:884292:884547:216023631	255	-
chr4	890220	890480	chr4:890220:890480:216023517	260	-
chr4	891793	892041	chr4:891793:892041:216023632	248	-
chr4	906495	906740	chr4:906495:906740:216023519	245	-
chr4	843648	843878	chr4:843648:843878:216023552	230	+
chr4	843426	843696	chr4:843426:843696:216023553	270	-
chr4	843832	844065	chr4:843832:844065:216023554	233	-
chr4	3128789	3129021	chr4:3128789:3129021:216023562	232	+
chr4	3129161	3129405	chr4:3129161:3129405:216023563	244	+
chr4	3128601	3128843	chr4:3128601:3128843:216023564	242	-
chr4	3128967	3129216	chr4:3128967:3129216:216023565	249	-
chr17	79657184	79657444	chr17:79657184:79657444:216023709	260	-
chr17	79660697	79660931	chr17:79660697:79660931:216023692	234	+
chr17	79660491	79660752	chr17:79660491:79660752:216023693	261	-
chr17	79660877	79661146	chr17:79660877:79661146:216023694	269	-
chr17	79663525	79663783	chr17:79663525:79663783:216023698	258	+
chr17	79663937	79664162	chr17:79663937:79664162:216023699	225	+
chr17	79663345	79663575	chr17:79663345:79663575:216023702	230	-
chr17	79663733	79663983	chr17:79663733:79663983:216023703	250	-
chr17	17124858	17125129	chr17:17124858:17125129:216023542	271	+
chr17	17124656	17124910	chr17:17124656:17124910:216023543	254	-
chr17	17116944	17117207	chr17:17116944:17117207:216023544	263	-
chr17	78093043	78093303	chr17:78093043:78093303:216023545	260	-
chr17	17120353	17120608	chr17:17120353:17120608:216023494	255	-
chr17	17122303	17122578	chr17:17122303:17122578:216023495	275	-
chr17	17127380	17127631	chr17:17127380:17127631:216023497	251	+
chr17	17127208	17127433	chr17:17127208:17127433:216023498	225	-
chr17	78090737	78091006	chr17:78090737:78091006:216023410	269	-
chr17	78083718	78083955	chr17:78083718:78083955:216023415	237	-
chr17	78085756	78085989	chr17:78085756:78085989:216023420	233	-

chr17	78086525	78086760	chr17:78086525:78086760:216023437	235	+
chr17	78086895	78087169	chr17:78086895:78087169:216023438	274	+
chr17	78086341	78086572	chr17:78086341:78086572:216023439	231	-
chr17	78086705	78086944	chr17:78086705:78086944:216023440	239	-
chr17	78087113	78087352	chr17:78087113:78087352:216023441	239	-
chr17	78082239	78082485	chr17:78082239:78082485:216023432	246	+
chr17	78082051	78082291	chr17:78082051:78082291:216023433	240	-
chr17	78082429	78082704	chr17:78082429:78082704:216023434	275	-
chr22	32887029	32887256	chr22:32887029:32887256:216023446	227	-
chr22	32891448	32891687	chr22:32891448:32891687:216023449	239	-
chr22	41542705	41542930	chr22:41542705:41542930:216023466	225	-
chr22	41522004	41522275	chr22:41522004:41522275:216023454	271	+
chr22	41521782	41522057	chr22:41521782:41522057:216023455	275	-
chr22	41523617	41523886	chr22:41523617:41523886:216023456	269	+
chr22	41523443	41523668	chr22:41523443:41523668:216023457	225	-
chr22	32880014	32880265	chr22:32880014:32880265:216023442	251	+
chr22	32879796	32880069	chr22:32879796:32880069:216023443	273	-
chr22	32881025	32881290	chr22:32881025:32881290:216023444	265	-
chr22	41548017	41548254	chr22:41548017:41548254:216023424	237	+
chr22	41547799	41548074	chr22:41547799:41548074:216023425	275	-
chr22	41548195	41548454	chr22:41548195:41548454:216023426	259	-
chr22	41545905	41546135	chr22:41545905:41546135:216023469	230	+
chr22	41545721	41545962	chr22:41545721:41545962:216023470	241	-
chr22	41546083	41546312	chr22:41546083:41546312:216023471	229	-
chr22	41550959	41551210	chr22:41550959:41551210:216023472	251	-
chr22	41553341	41553567	chr22:41553341:41553567:216023473	226	+
chr22	41553141	41553391	chr22:41553141:41553391:216023474	250	-
chr22	41556618	41556845	chr22:41556618:41556845:216023476	227	-
chr22	41558697	41558950	chr22:41558697:41558950:216023477	253	-
chr22	41560027	41560284	chr22:41560027:41560284:216023478	257	-
chr22	41562501	41562736	chr22:41562501:41562736:216023479	235	-
chr22	41566378	41566625	chr22:41566378:41566625:216023481	247	-
chr22	41568471	41568698	chr22:41568471:41568698:216023482	227	-
chr22	32871174	32871447	chr22:32871174:32871447:216023523	273	+
chr22	32870952	32871223	chr22:32870952:32871223:216023524	271	-
chr22	32871390	32871655	chr22:32871390:32871655:216023525	265	-
chr22	41572287	41572521	chr22:41572287:41572521:216023526	234	+
chr22	41572651	41572876	chr22:41572651:41572876:216023527	225	+
chr22	41573001	41573229	chr22:41573001:41573229:216023528	228	+
chr22	41573349	41573575	chr22:41573349:41573575:216023529	226	+
chr22	41573695	41573921	chr22:41573695:41573921:216023530	226	+
chr22	41574041	41574268	chr22:41574041:41574268:216023531	227	+
chr22	41574389	41574660	chr22:41574389:41574660:216023532	271	+
chr22	41574799	41575072	chr22:41574799:41575072:216023533	273	+
chr22	41572115	41572342	chr22:41572115:41572342:216023534	227	-
chr22	41572467	41572702	chr22:41572467:41572702:216023535	235	-
chr22	41572825	41573050	chr22:41572825:41573050:216023536	225	-

chr22	41573179	41573404	chr22:41573179:41573404:216023537	225	-
chr22	41573523	41573749	chr22:41573523:41573749:216023538	226	-
chr22	41573867	41574096	chr22:41573867:41574096:216023539	229	-
chr22	41574213	41574445	chr22:41574213:41574445:216023540	232	-
chr22	41574605	41574854	chr22:41574605:41574854:216023541	249	-
chr9	21974603	21974829	chr9:21974603:21974829:216023398	226	+
chr9	21974425	21974653	chr9:21974425:21974653:216023399	228	-
chr9	21974777	21975048	chr9:21974777:21975048:216023400	271	-
chr9	21994308	21994570	chr9:21994308:21994570:216023402	262	+
chr9	21994110	21994361	chr9:21994110:21994361:216023403	251	-
chr1	32796355	32796594	chr1:32796355:32796594:216023616	239	+
chr1	32796139	32796410	chr1:32796139:32796410:216023617	271	-
chr1	32793109	32793354	chr1:32793109:32793354:216023659	245	-
chr1	32757741	32758013	chr1:32757741:32758013:216023657	272	-
chr1	32790048	32790283	chr1:32790048:32790283:216023662	235	-
chr1	32798442	32798712	chr1:32798442:32798712:216023700	270	+
chr1	32798262	32798496	chr1:32798262:32798496:216023701	234	-
chr1	155205444	155205712	chr1:155205444:155205712:216023581	268	-
chr1	155210393	155210641	chr1:155210393:155210641:216023582	248	-
chr1	20974968	20975229	chr1:20974968:20975229:216023049	261	-
chr1	20975660	20975935	chr1:20975660:20975935:216023043	275	+
chr1	20975462	20975714	chr1:20975462:20975714:216023044	252	-
chr1	20977071	20977346	chr1:20977071:20977346:216023046	275	+
chr1	20976899	20977126	chr1:20976899:20977126:216023047	227	-
chr1	87181527	87181800	chr1:87181527:87181800:216024023	273	+
chr1	87181309	87181582	chr1:87181309:87181582:216024024	273	-
chr1	87200730	87200968	chr1:87200730:87200968:216024027	238	-
chr1	203831274	203831505	chr1:203831274:203831505:216024033	231	-
chr11	6414576	6414851	chr11:6414576:6414851:216024002	275	+
chr11	6414966	6415193	chr11:6414966:6415193:216024003	227	+
chr11	6415314	6415557	chr11:6415314:6415557:216024004	243	+
chr11	6415714	6415981	chr11:6415714:6415981:216024005	267	+
chr11	6414404	6414630	chr11:6414404:6414630:216024006	226	-
chr11	6414794	6415019	chr11:6414794:6415019:216024007	225	-
chr11	6415138	6415367	chr11:6415138:6415367:216024008	229	-
chr11	6415502	6415766	chr11:6415502:6415766:216024009	264	-
chr11	47264420	47264659	chr11:47264420:47264659:216023140	239	+
chr11	47264822	47265069	chr11:47264822:47265069:216023141	247	+
chr11	47264238	47264473	chr11:47264238:47264473:216023142	235	-
chr11	47264606	47264877	chr11:47264606:47264877:216023143	271	-
chr11	46667382	46667617	chr11:46667382:46667617:216023129	235	-
chr11	1780718	1780983	chr11:1780718:1780983:216023414	265	-
chr11	1780171	1780398	chr11:1780171:1780398:216023422	227	-
chr11	111781009	111781252	chr11:111781009:111781252:216023423	243	-
chr11	1778714	1778941	chr11:1778714:1778941:216023411	227	+
chr11	1778528	1778768	chr11:1778528:1778768:216023412	240	-
chr11	46679042	46679269	chr11:46679042:46679269:216023154	227	-

chr11	46680900	46681155	chr11:46680900:46681155:216023155	255	-
chr11	47266258	47266514	chr11:47266258:47266514:216023146	256	-
chr11	47269164	47269401	chr11:47269164:47269401:216023147	237	-
chr11	46665718	46665989	chr11:46665718:46665989:216023149	271	-
chr11	46671701	46671970	chr11:46671701:46671970:216023151	269	-
chr11	46670531	46670784	chr11:46670531:46670784:216023161	253	-
chr11	47261640	47261865	chr11:47261640:47261865:216023163	225	-
chr11	111779463	111779718	chr11:111779463:111779718:216023520	255	-
chr14	88459465	88459713	chr14:88459465:88459713:216023639	248	+
chr14	88459253	88459519	chr14:88459253:88459519:216023640	266	-
chr14	88459657	88459926	chr14:88459657:88459926:216023641	269	-
chr14	88414035	88414266	chr14:88414035:88414266:216023726	231	-
chr14	88416138	88416369	chr14:88416138:88416369:216023719	231	-
chr14	88417042	88417317	chr14:88417042:88417317:216023716	275	+
chr14	88416872	88417097	chr14:88416872:88417097:216023717	225	-
chr14	88406214	88406485	chr14:88406214:88406485:216023714	271	-
chr14	88407860	88408131	chr14:88407860:88408131:216023723	271	+
chr14	88407690	88407917	chr14:88407690:88407917:216023724	227	-
chr14	88434754	88434979	chr14:88434754:88434979:216023711	225	+
chr14	88434584	88434809	chr14:88434584:88434809:216023712	225	-
chr14	88450691	88450918	chr14:88450691:88450918:216023721	227	-
chr14	73637673	73637920	chr14:73637673:73637920:216023861	247	+
chr14	73637471	73637725	chr14:73637471:73637725:216023862	254	-
chr14	73664687	73664958	chr14:73664687:73664958:216023867	271	-
chr14	73683796	73684061	chr14:73683796:73684061:216023870	265	-
chr14	73614684	73614926	chr14:73614684:73614926:216023892	242	-
chr14	73685814	73686067	chr14:73685814:73686067:216023918	253	-
chr2	65315760	65316005	chr2:65315760:65316005:216023966	245	+
chr2	65316120	65316353	chr2:65316120:65316353:216023967	233	+
chr2	65315590	65315815	chr2:65315590:65315815:216023968	225	-
chr2	65315950	65316175	chr2:65315950:65316175:216023969	225	-
chr2	65356997	65357240	chr2:65356997:65357240:216023980	243	-
chr2	168994564	168994790	chr2:168994564:168994790:216024018	226	-
chr2	65318087	65318360	chr2:65318087:65318360:216023977	273	-
chr2	86737443	86737678	chr2:86737443:86737678:216023405	235	-
chr2	86769337	86769572	chr2:86769337:86769572:216023409	235	-
chr2	86790399	86790668	chr2:86790399:86790668:216023407	269	-
chr2	135625119	135625372	chr2:135625119:135625372:216023192	253	-
chr2	135628435	135628662	chr2:135628435:135628662:216023193	227	-
chr2	135616801	135617034	chr2:135616801:135617034:216023075	233	-
chr2	135655863	135656134	chr2:135655863:135656134:216023078	271	-
chr2	135659240	135659465	chr2:135659240:135659465:216023195	225	-
chr2	220091561	220091806	chr2:220091561:220091806:216023355	245	-
chr2	220085354	220085626	chr2:220085354:220085626:216023255	272	+
chr2	220085776	220086001	chr2:220085776:220086001:216023256	225	+
chr2	220085134	220085405	chr2:220085134:220085405:216023257	271	-
chr2	220085574	220085829	chr2:220085574:220085829:216023258	255	-

chr2	220085948	220086211	chr2:220085948:220086211:216023259	263	-
chr2	220088341	220088566	chr2:220088341:220088566:216023265	225	+
chr2	220088683	220088908	chr2:220088683:220088908:216023266	225	+
chr2	220089027	220089254	chr2:220089027:220089254:216023267	227	+
chr2	220089373	220089598	chr2:220089373:220089598:216023268	225	+
chr2	220089711	220089937	chr2:220089711:220089937:216023269	226	+
chr2	220090061	220090312	chr2:220090061:220090312:216023270	251	+
chr2	220088171	220088396	chr2:220088171:220088396:216023271	225	-
chr2	220088511	220088740	chr2:220088511:220088740:216023272	229	-
chr2	220088853	220089078	chr2:220088853:220089078:216023273	225	-
chr2	220089199	220089425	chr2:220089199:220089425:216023274	226	-
chr2	220089543	220089768	chr2:220089543:220089768:216023275	225	-
chr2	220089879	220090116	chr2:220089879:220090116:216023276	237	-
chr2	220090257	220090528	chr2:220090257:220090528:216023277	271	-
chr20	4680005	4680231	chr20:4680005:4680231:216023913	226	+
chr20	4680385	4680616	chr20:4680385:4680616:216023914	231	+
chr20	4679827	4680052	chr20:4679827:4680052:216023915	225	-
chr20	4680179	4680440	chr20:4680179:4680440:216023916	261	-
chr20	4680561	4680796	chr20:4680561:4680796:216023917	235	-
chr3	53226094	53226368	chr3:53226094:53226368:216023912	274	-
chr3	53212411	53212670	chr3:53212411:53212670:216023905	259	-
chr3	53218862	53219137	chr3:53218862:53219137:216023813	275	-
chr3	128525395	128525661	chr3:128525395:128525661:216023817	266	+
chr3	128525185	128525450	chr3:128525185:128525450:216023818	265	-
chr3	128526358	128526621	chr3:128526358:128526621:216023819	263	-
chr3	53215399	53215627	chr3:53215399:53215627:216023055	228	+
chr3	53215189	53215450	chr3:53215189:53215450:216023056	261	-
chr3	53215573	53215834	chr3:53215573:53215834:216023057	261	-
chr3	53219746	53219997	chr3:53219746:53219997:216023061	251	+
chr3	53220146	53220414	chr3:53220146:53220414:216023062	268	+
chr3	53220576	53220817	chr3:53220576:53220817:216023063	241	+
chr3	53219570	53219799	chr3:53219570:53219799:216023064	229	-
chr3	53219942	53220196	chr3:53219942:53220196:216023065	254	-
chr3	53220364	53220630	chr3:53220364:53220630:216023066	266	-
chr3	43756341	43756591	chr3:43756341:43756591:216023068	250	-
chr3	11356786	11357045	chr3:11356786:11357045:216023336	259	-
chr3	11350428	11350699	chr3:11350428:11350699:216023334	271	-
chr3	11400054	11400281	chr3:11400054:11400281:216023342	227	+
chr3	11399862	11400109	chr3:11399862:11400109:216023343	247	-
chr3	11402203	11402430	chr3:11402203:11402430:216023344	227	+
chr3	11402025	11402258	chr3:11402025:11402258:216023345	233	-
chr3	11421414	11421671	chr3:11421414:11421671:216023348	257	-
chr3	11468230	11468483	chr3:11468230:11468483:216023349	253	-
chr3	11596253	11596516	chr3:11596253:11596516:216023350	263	-
chr3	11340124	11340391	chr3:11340124:11340391:216023331	267	-
chr3	11340804	11341059	chr3:11340804:11341059:216023332	255	-
chr3	43732455	43732730	chr3:43732455:43732730:216023071	275	-

chr3	43759903	43760154	chr3:43759903:43760154:216023073	251	-
chr3	119545608	119545845	chr3:119545608:119545845:216023664	237	-
chr3	119624570	119624843	chr3:119624570:119624843:216023599	273	-
chr3	119631615	119631854	chr3:119631615:119631854:216023600	239	+
chr3	119631439	119631668	chr3:119631439:119631668:216023601	229	-
chr3	119582412	119582639	chr3:119582412:119582639:216023594	227	+
chr3	119582236	119582466	chr3:119582236:119582466:216023595	230	-
chr3	119585395	119585652	chr3:119585395:119585652:216023596	257	-
chr6	106763946	106764181	chr6:106763946:106764181:216023127	235	-
chr6	106695979	106696228	chr6:106695979:106696228:216023110	249	-
chr6	106727648	106727887	chr6:106727648:106727887:216023111	239	+
chr6	106727456	106727703	chr6:106727456:106727703:216023112	247	-
chr8	17916981	17917208	chr8:17916981:17917208:216023101	227	+
chr8	17916815	17917041	chr8:17916815:17917041:216023102	226	-
chr8	17917149	17917406	chr8:17917149:17917406:216023103	257	-
chr8	17914997	17915223	chr8:17914997:17915223:216023377	226	-
chr8	17916297	17916550	chr8:17916297:17916550:216023375	253	-
chr8	17921927	17922192	chr8:17921927:17922192:216023373	265	-
chr8	17930730	17930957	chr8:17930730:17930957:216023383	227	-
chr8	17933015	17933240	chr8:17933015:17933240:216023379	225	-
chr8	17942148	17942414	chr8:17942148:17942414:216023381	266	-
chr8	53540550	53540779	chr8:53540550:53540779:216023919	229	-
chr8	53547449	53547700	chr8:53547449:53547700:216023950	251	-
chr8	53589067	53589300	chr8:53589067:53589300:216023951	233	+
chr8	53588895	53589122	chr8:53588895:53589122:216023952	227	-
chr8	53568602	53568828	chr8:53568602:53568828:216023923	226	+
chr8	53568904	53569130	chr8:53568904:53569130:216023924	226	+
chr8	53569208	53569433	chr8:53569208:53569433:216023925	225	+
chr8	53569538	53569763	chr8:53569538:53569763:216023926	225	+
chr8	53569844	53570070	chr8:53569844:53570070:216023927	226	+
chr8	53570174	53570440	chr8:53570174:53570440:216023928	266	+
chr8	53570602	53570863	chr8:53570602:53570863:216023929	261	+
chr8	53568436	53568662	chr8:53568436:53568662:216023930	226	-
chr8	53568764	53568990	chr8:53568764:53568990:216023931	226	-
chr8	53569040	53569265	chr8:53569040:53569265:216023932	225	-
chr8	53569374	53569599	chr8:53569374:53569599:216023933	225	-
chr8	53569678	53569904	chr8:53569678:53569904:216023934	226	-
chr8	53570004	53570229	chr8:53570004:53570229:216023935	225	-
chr8	53570386	53570656	chr8:53570386:53570656:216023936	270	-
chr8	53571355	53571596	chr8:53571355:53571596:216023944	241	-
chr8	53580549	53580790	chr8:53580549:53580790:216023945	241	-
chr8	53536219	53536464	chr8:53536219:53536464:216023976	245	-
chr8	53597911	53598156	chr8:53597911:53598156:216023975	245	-
chrX	48661282	48661517	chrX:48661282:48661517:216023665	235	+
chrX	48661062	48661337	chrX:48661062:48661337:216023666	275	-
chrX	48661468	48661721	chrX:48661468:48661721:216023667	253	-
chrX	48673017	48673257	chrX:48673017:48673257:216023668	240	+

chrX	48673425	48673684	chrX:48673425:48673684:216023669	259	+
chrX	48672807	48673068	chrX:48672807:48673068:216023670	261	-
chrX	48673207	48673477	chrX:48673207:48673477:216023671	270	-
chrX	48675707	48675955	chrX:48675707:48675955:216023635	248	-
chrX	48664939	48665170	chrX:48664939:48665170:216023609	231	+
chrX	48664733	48664994	chrX:48664733:48664994:216023610	261	-
chrX	48676602	48676841	chrX:48676602:48676841:216023613	239	+
chrX	48676408	48676657	chrX:48676408:48676657:216023614	249	-
chrX	48676790	48677055	chrX:48676790:48677055:216023615	265	-
chr10	121429527	121429796	chr10:121429527:121429796:216023208	269	+
chr10	121429327	121429582	chr10:121429327:121429582:216023209	255	-
chr10	121431923	121432148	chr10:121431923:121432148:216023210	225	+
chr10	121431741	121431978	chr10:121431741:121431978:216023211	237	-
chr10	121432097	121432346	chr10:121432097:121432346:216023212	249	-
chr10	73610911	73611174	chr10:73610911:73611174:216023970	263	-
chr10	73581593	73581841	chr10:73581593:73581841:216023874	248	-
chr10	73594103	73594350	chr10:73594103:73594350:216023807	247	-
chr10	73587737	73588002	chr10:73587737:73588002:216023808	265	-
chr10	73578346	73578581	chr10:73578346:73578581:216023810	235	-
chr10	73590853	73591080	chr10:73590853:73591080:216023802	227	-
chr10	73582381	73582614	chr10:73582381:73582614:216023805	233	-
chr21	27372302	27372539	chr21:27372302:27372539:216023094	237	-
chr21	27354629	27354854	chr21:27354629:27354854:216023092	225	-
chr21	27462229	27462462	chr21:27462229:27462462:216023100	233	-
chr21	27253954	27254189	chr21:27253954:27254189:216023128	235	-
chr21	27542776	27543002	chr21:27542776:27543002:216023145	226	-
chr21	27264004	27264272	chr21:27264004:27264272:216023083	268	-
chr21	27269853	27270088	chr21:27269853:27270088:216023084	235	-
chr21	27284217	27284484	chr21:27284217:27284484:216023086	267	+
chr21	27284025	27284272	chr21:27284025:27284272:216023087	247	-
chr21	27326874	27327127	chr21:27326874:27327127:216023088	253	-
chr4	15709102	15709375	chr4:15709102:15709375:216023366	273	-
chr4	15716876	15717141	chr4:15716876:15717141:216023364	265	-
chr4	3131810	3132067	chr4:3131810:3132067:216023820	257	+
chr4	3131596	3131867	chr4:3131596:3131867:216023821	271	-
chr4	3132010	3132247	chr4:3132010:3132247:216023822	237	-
chr4	3137746	3138007	chr4:3137746:3138007:216023829	261	+
chr4	3137572	3137799	chr4:3137572:3137799:216023830	227	-
chr4	3137952	3138187	chr4:3137952:3138187:216023831	235	-
chr4	3176583	3176811	chr4:3176583:3176811:216023832	228	+
chr4	3176385	3176638	chr4:3176385:3176638:216023833	253	-
chr4	3176757	3176986	chr4:3176757:3176986:216023834	229	-
chr4	3237160	3237386	chr4:3237160:3237386:216023843	226	+
chr4	3236958	3237214	chr4:3236958:3237214:216023844	256	-
chr4	3237336	3237607	chr4:3237336:3237607:216023845	271	-
chr4	90743367	90743622	chr4:90743367:90743622:216023992	255	-
chr4	90749262	90749509	chr4:90749262:90749509:216023993	247	-

chr4	3136096	3136369	chr4:3136096:3136369:216023853	273	-
chr4	3146847	3147086	chr4:3146847:3147086:216023854	239	-
chr4	3149874	3150103	chr4:3149874:3150103:216023855	229	+
chr4	3149702	3149932	chr4:3149702:3149932:216023856	230	-
chr4	3219462	3219721	chr4:3219462:3219721:216023859	259	-
chr4	90647752	90648007	chr4:90647752:90648007:216024010	255	-
chr6	74320086	74320347	chr6:74320086:74320347:216024075	261	-
chr6	74348196	74348421	chr6:74348196:74348421:216024080	225	+
chr6	74348012	74348249	chr6:74348012:74348249:216024081	237	-
chr6	74354117	74354344	chr6:74354117:74354344:216024084	227	+
chr6	74353931	74354171	chr6:74353931:74354171:216024085	240	-
chr6	74354289	74354564	chr6:74354289:74354564:216024086	275	-
chr6	74363485	74363742	chr6:74363485:74363742:216024087	257	-
chr6	31607990	31608217	chr6:31607990:31608217:216023288	227	+
chr6	31608332	31608558	chr6:31608332:31608558:216023289	226	+
chr6	31608670	31608896	chr6:31608670:31608896:216023290	226	+
chr6	31609008	31609233	chr6:31609008:31609233:216023291	225	+
chr6	31609344	31609570	chr6:31609344:31609570:216023292	226	+
chr6	31609654	31609882	chr6:31609654:31609882:216023293	228	+
chr19	7592550	7592820	chr19:7592550:7592820:216022969	270	+
chr19	7592958	7593217	chr19:7592958:7593217:216022970	259	+
chr19	7592326	7592599	chr19:7592326:7592599:216022971	273	-
chr19	7592764	7593012	chr19:7592764:7593012:216022972	248	-
chr19	7598551	7598778	chr19:7598551:7598778:216022979	227	+
chr19	7598371	7598601	chr19:7598371:7598601:216022980	230	-
chr19	7589819	7590073	chr19:7589819:7590073:216022717	254	-
chr19	7595310	7595537	chr19:7595310:7595537:216022904	227	+
chr19	7595136	7595364	chr19:7595136:7595364:216022905	228	-
chr16	56623687	56623929	chr16:56623687:56623929:216022984	242	+
chr16	56623467	56623742	chr16:56623467:56623742:216022985	275	-
chr18	21166213	21166484	chr18:21166213:21166484:216022981	271	-
chr18	21112137	21112412	chr18:21112137:21112412:216022982	275	-
chr18	21113289	21113514	chr18:21113289:21113514:216022906	225	-
chr18	21148749	21149012	chr18:21148749:21149012:216022910	263	-
chr18	21118478	21118721	chr18:21118478:21118721:216022914	243	-
chr18	21123385	21123616	chr18:21123385:21123616:216022916	231	-
chr18	21116609	21116872	chr18:21116609:21116872:216022922	263	-
chr18	21119289	21119560	chr18:21119289:21119560:216022924	271	-
chr18	21121176	21121435	chr18:21121176:21121435:216022932	259	+
chr18	21120992	21121231	chr18:21120992:21121231:216022933	239	-
chr6	161969858	161970105	chr6:161969858:161970105:216022934	247	-
chr3	182853475	182853708	chr3:182853475:182853708:216022894	233	-
chr3	182858296	182858521	chr3:182858296:182858521:216022895	225	-
chr3	182880371	182880616	chr3:182880371:182880616:216022901	245	-
chr8	98863599	98863824	chr8:98863599:98863824:216022892	225	-
chr8	98827468	98827693	chr8:98827468:98827693:216022888	225	-
chr8	98831338	98831612	chr8:98831338:98831612:216022890	274	-

chr12	40619114	40619367	chr12:40619114:40619367:216022865	253	+
chr12	40618894	40619167	chr12:40618894:40619167:216022866	273	-
chr12	40619312	40619541	chr12:40619312:40619541:216022867	229	-
chr12	40668527	40668768	chr12:40668527:40668768:216022844	241	+
chr12	40668345	40668584	chr12:40668345:40668584:216022845	239	-
chr12	40668713	40668976	chr12:40668713:40668976:216022846	263	-
chr12	40715963	40716238	chr12:40715963:40716238:216022850	275	+
chr12	40715795	40716020	chr12:40715795:40716020:216022851	225	-
chr12	40716183	40716418	chr12:40716183:40716418:216022852	235	-
chr18	21131559	21131792	chr18:21131559:21131792:216022925	233	-
chr18	21124984	21125210	chr18:21124984:21125210:216022918	226	+
chr18	21124800	21125040	chr18:21124800:21125040:216022919	240	-
chr18	21134876	21135119	chr18:21134876:21135119:216022920	243	+
chr18	21134692	21134930	chr18:21134692:21134930:216022921	238	-
chrX	119581872	119582109	chrX:119581872:119582109:216022766	237	+
chrX	119581652	119581927	chrX:119581652:119581927:216022767	275	-
chrX	119582779	119583028	chrX:119582779:119583028:216022768	249	-
chrX	119576426	119576701	chrX:119576426:119576701:216022764	275	-
chr12	40713898	40714123	chr12:40713898:40714123:216022805	225	+
chr12	40713692	40713952	chr12:40713692:40713952:216022806	260	-
chr12	40749881	40750152	chr12:40749881:40750152:216022821	271	-
chr12	40646786	40647053	chr12:40646786:40647053:216022780	267	+
chr12	40646588	40646840	chr12:40646588:40646840:216022781	252	-
chr9	135782086	135782341	chr9:135782086:135782341:216024123	255	-
chr9	35060452	35060695	chr9:35060452:35060695:216024154	243	+
chr9	35060820	35061089	chr9:35060820:35061089:216024155	269	+
chr9	35060270	35060507	chr9:35060270:35060507:216024156	237	-
chr9	35060640	35060874	chr9:35060640:35060874:216024157	234	-
chr9	35061034	35061295	chr9:35061034:35061295:216024158	261	-
chr9	35072304	35072544	chr9:35072304:35072544:216024164	240	-
chr9	35057263	35057530	chr9:35057263:35057530:216024179	267	+
chr9	35057081	35057318	chr9:35057081:35057318:216024180	237	-
chr9	35057475	35057728	chr9:35057475:35057728:216024181	253	-
chr9	86279913	86280178	chr9:86279913:86280178:216024260	265	-
chr9	86322379	86322651	chr9:86322379:86322651:216024263	272	-
chr9	86294874	86295147	chr9:86294874:86295147:216024139	273	+
chr9	86294658	86294929	chr9:86294658:86294929:216024140	271	-
chr9	86276773	86276998	chr9:86276773:86276998:216024142	225	+
chr9	86276599	86276828	chr9:86276599:86276828:216024143	229	-
chr12	132380298	132380556	chr12:132380298:132380556:216024189	258	+
chr12	132380102	132380353	chr12:132380102:132380353:216024190	251	-
chr12	132392008	132392268	chr12:132392008:132392268:216024183	260	-
chr12	132395227	132395498	chr12:132395227:132395498:216023031	271	-
chr12	132400596	132400826	chr12:132400596:132400826:216024201	230	+
chr12	132400950	132401209	chr12:132400950:132401209:216024202	259	+
chr12	132400380	132400646	chr12:132400380:132400646:216024203	266	-
chr12	132400776	132401002	chr12:132400776:132401002:216024204	226	-

chr12	132394452	132394692	chr12:132394452:132394692:216024195	240	+
chr12	132394838	132395101	chr12:132394838:132395101:216024196	263	+
chr12	132394280	132394505	chr12:132394280:132394505:216024197	225	-
chr12	132394644	132394891	chr12:132394644:132394891:216024198	247	-
chr12	132405829	132406098	chr12:132405829:132406098:216024208	269	+
chr12	132405605	132405877	chr12:132405605:132405877:216024209	272	-
chr15	42500239	42500492	chr15:42500239:42500492:216024254	253	-
chr14	68120112	68120377	chr14:68120112:68120377:216023034	265	-
chr1	78186825	78187056	chr1:78186825:78187056:216024215	231	-
chr1	78187636	78187890	chr1:78187636:78187890:216024217	254	+
chr1	78187456	78187693	chr1:78187456:78187693:216024218	237	-
chr1	78187834	78188109	chr1:78187834:78188109:216024219	275	-
chr1	78180269	78180529	chr1:78180269:78180529:216024212	260	-
chr1	78181395	78181648	chr1:78181395:78181648:216024213	253	-
chr1	78199950	78200201	chr1:78199950:78200201:216024236	251	-
chr1	78204918	78205191	chr1:78204918:78205191:216024237	273	-
chr1	78167106	78167331	chr1:78167106:78167331:216024226	225	+
chr1	78166928	78167161	chr1:78166928:78167161:216024227	233	-
chr1	78201709	78201980	chr1:78201709:78201980:216024223	271	-
chr1	78191275	78191522	chr1:78191275:78191522:216024229	247	-
chr1	78194146	78194383	chr1:78194146:78194383:216024230	237	+
chr1	78193948	78194205	chr1:78193948:78194205:216024231	257	-
chr1	78194328	78194553	chr1:78194328:78194553:216024232	225	-
chr15	42470574	42470829	chr15:42470574:42470829:216024176	255	+
chr15	42470376	42470629	chr15:42470376:42470629:216024177	253	-
chr15	42456528	42456773	chr15:42456528:42456773:216024178	245	-
chr15	42455722	42455949	chr15:42455722:42455949:216024170	227	+
chr15	42455512	42455775	chr15:42455512:42455775:216024171	263	-
chr15	42455894	42456119	chr15:42455894:42456119:216024172	225	-
chr15	42452925	42453198	chr15:42452925:42453198:216024239	273	-
chr15	42465891	42466142	chr15:42465891:42466142:216024245	251	-
chr15	42483291	42483566	chr15:42483291:42483566:216024251	275	-
chr15	42483666	42483911	chr15:42483666:42483911:216024252	245	-
chr15	42491982	42492225	chr15:42491982:42492225:216024253	243	-
chr15	42479423	42479656	chr15:42479423:42479656:216024248	233	-
chr11	18541034	18541271	chr11:18541034:18541271:216024131	237	-
chr11	18531167	18531400	chr11:18531167:18531400:216024128	233	+
chr11	18530969	18531222	chr11:18530969:18531222:216024129	253	-
chr11	18536305	18536540	chr11:18536305:18536540:216024136	235	+
chr11	18536133	18536360	chr11:18536133:18536360:216024137	227	-
chr11	18505575	18505806	chr11:18505575:18505806:216024125	231	+
chr11	18505369	18505629	chr11:18505369:18505629:216024126	260	-
chr11	18524001	18524274	chr11:18524001:18524274:216024134	273	-
chr9	35068032	35068299	chr9:35068032:35068299:216024161	267	+
chr9	35067848	35068087	chr9:35067848:35068087:216024162	239	-
chr9	35068244	35068489	chr9:35068244:35068489:216024163	245	-
chr9	35061546	35061789	chr9:35061546:35061789:216024144	243	-

chr9	35062941	35063190	chr9:35062941:35063190:216024145	249	-
chr12	132401932	132402192	chr12:132401932:132402192:216024187	260	-
chr12	132403018	132403259	chr12:132403018:132403259:216024188	241	-
chr12	132397645	132397892	chr12:132397645:132397892:216024185	247	-
chr14	68141066	68141339	chr14:68141066:68141339:216023035	273	-
chr12	132396459	132396726	chr12:132396459:132396726:216024184	267	-
chr9	135804108	135804375	chr9:135804108:135804375:216024115	267	-
chr9	135797174	135797415	chr9:135797174:135797415:216024120	241	-
chr9	135798705	135798980	chr9:135798705:135798980:216024119	275	-
chr9	135787809	135788046	chr9:135787809:135788046:216024113	237	+
chr9	135787637	135787866	chr9:135787637:135787866:216024114	229	-
chr9	135771797	135772069	chr9:135771797:135772069:216024116	272	+
chr9	135771595	135771855	chr9:135771595:135771855:216024117	260	-
chr9	135772021	135772296	chr9:135772021:135772296:216024118	275	-
chr9	135776072	135776321	chr9:135776072:135776321:216024112	249	-
chr17	7577457	7577683	chr17:7577457:7577683:216024103	226	-
chr17	7579488	7579758	chr17:7579488:7579758:216024099	270	+
chr17	7579274	7579543	chr17:7579274:7579543:216024100	269	-
chr17	7579704	7579973	chr17:7579704:7579973:216024101	269	-
chr17	7576692	7576929	chr17:7576692:7576929:216024093	237	+
chr17	7577092	7577335	chr17:7577092:7577335:216024094	243	+
chr17	7576482	7576743	chr17:7576482:7576743:216024095	261	-
chr17	7576876	7577147	chr17:7576876:7577147:216024096	271	-
chr20	33297214	33297475	chr20:33297214:33297475:216024107	261	+
chr20	33296998	33297268	chr20:33296998:33297268:216024108	270	-
chr20	33298056	33298285	chr20:33298056:33298285:216024105	229	+
chr20	33297836	33298111	chr20:33297836:33298111:216024106	275	-
chr6	74304766	74305035	chr6:74304766:74305035:216024091	269	-
chr9	135785924	135786198	chr9:135785924:135786198:216024109	274	-
chr9	135776946	135777221	chr9:135776946:135777221:216024110	275	-
chr9	135782658	135782884	chr9:135782658:135782884:216024111	226	-
chr12	56113041	56113270	chr12:56113041:56113270:216023321	229	+
chr12	56112821	56113096	chr12:56112821:56113096:216023322	275	-
chr12	56113217	56113476	chr12:56113217:56113476:216023323	259	-
chr3	11354724	11354985	chr3:11354724:11354985:216023335	261	-
chr3	43753169	43753416	chr3:43753169:43753416:216023074	247	-
chr3	43759131	43759394	chr3:43759131:43759394:216023072	263	-
chr8	17919005	17919233	chr8:17919005:17919233:216023104	228	+
chr8	17918783	17919058	chr8:17918783:17919058:216023105	275	-
chr8	17919177	17919432	chr8:17919177:17919432:216023106	255	-
chr10	121411160	121411435	chr10:121411160:121411435:216023213	275	-
chr10	121436104	121436358	chr10:121436104:121436358:216023280	254	+
chr10	121436500	121436725	chr10:121436500:121436725:216023281	225	+
chr10	121435922	121436161	chr10:121435922:121436161:216023282	239	-
chr10	121436306	121436555	chr10:121436306:121436555:216023283	249	-
chr10	121436670	121436941	chr10:121436670:121436941:216023284	271	-
chr21	27512441	27512684	chr21:27512441:27512684:216023144	243	-

chr21	27369647	27369880	chr21:27369647:27369880:216023093	233	-
chr21	27394318	27394573	chr21:27394318:27394573:216023095	255	+
chr21	27394128	27394372	chr21:27394128:27394372:216023096	244	-
chr21	27423470	27423699	chr21:27423470:27423699:216023097	229	+
chr21	27423288	27423526	chr21:27423288:27423526:216023098	238	-
chr21	27425520	27425791	chr21:27425520:27425791:216023099	271	-
chr21	27484260	27484515	chr21:27484260:27484515:216023107	255	-
chr21	27327911	27328160	chr21:27327911:27328160:216023089	249	-
chr21	27347357	27347596	chr21:27347357:27347596:216023090	239	-
chr21	27348229	27348503	chr21:27348229:27348503:216023091	274	-
chr21	27277308	27277545	chr21:27277308:27277545:216023085	237	-
chr22	51065165	51065426	chr22:51065165:51065426:216023121	261	+
chr22	51065591	51065817	chr22:51065591:51065817:216023122	226	+
chr22	51065969	51066243	chr22:51065969:51066243:216023123	274	+
chr22	51064983	51065215	chr22:51064983:51065215:216023124	232	-
chr22	51065375	51065640	chr22:51065375:51065640:216023125	265	-
chr22	51065767	51066022	chr22:51065767:51066022:216023126	255	-
chr11	47269563	47269798	chr11:47269563:47269798:216023108	235	-
chr11	47267010	47267258	chr11:47267010:47267258:216023080	248	+
chr11	47266818	47267064	chr11:47266818:47267064:216023081	246	-
chr11	47267204	47267477	chr11:47267204:47267477:216023082	273	-
chr11	47270199	47270458	chr11:47270199:47270458:216023148	259	-
chr1	17338196	17338431	chr1:17338196:17338431:216023245	235	-
chr1	63299669	63299894	chr1:63299669:63299894:216023174	225	-
chr1	63269428	63269702	chr1:63269428:63269702:216023164	274	-
chr1	63270891	63271134	chr1:63270891:63271134:216023165	243	+
chr1	63270721	63270950	chr1:63270721:63270950:216023166	229	-
chr1	63282424	63282671	chr1:63282424:63282671:216023167	247	+
chr1	63282212	63282480	chr1:63282212:63282480:216023168	268	-
chr1	17328665	17328933	chr1:17328665:17328933:216023230	268	+
chr1	17328491	17328720	chr1:17328491:17328720:216023231	229	-
chr1	17330989	17331216	chr1:17330989:17331216:216023232	227	+
chr1	17331353	17331593	chr1:17331353:17331593:216023233	240	+
chr1	17330791	17331044	chr1:17330791:17331044:216023234	253	-
chr1	17331163	17331405	chr1:17331163:17331405:216023235	242	-
chr1	17331539	17331779	chr1:17331539:17331779:216023236	240	-
chr1	17322649	17322921	chr1:17322649:17322921:216023224	272	+
chr1	17322425	17322699	chr1:17322425:17322699:216023225	274	-
chr1	17322865	17323100	chr1:17322865:17323100:216023226	235	-
chr1	17316370	17316626	chr1:17316370:17316626:216023216	256	+
chr1	17316152	17316425	chr1:17316152:17316425:216023217	273	-
chr1	17316574	17316849	chr1:17316574:17316849:216023218	275	-
chr1	17312503	17312729	chr1:17312503:17312729:216023246	226	+
chr1	17312829	17313055	chr1:17312829:17313055:216023247	226	+
chr1	17313123	17313349	chr1:17313123:17313349:216023248	226	+
chr1	17313521	17313755	chr1:17313521:17313755:216023249	234	+
chr1	17312347	17312573	chr1:17312347:17312573:216023250	226	-

chr1	17312681	17312906	chr1:17312681:17312906:216023251	225	-
chr1	17312977	17313203	chr1:17312977:17313203:216023252	226	-
chr1	17313299	17313567	chr1:17313299:17313567:216023253	268	-
chr1	17313701	17313960	chr1:17313701:17313960:216023254	259	-
chr1	20960192	20960424	chr1:20960192:20960424:216023052	232	+
chr1	20959972	20960241	chr1:20959972:20960241:216023053	269	-
chr1	20960370	20960595	chr1:20960370:20960595:216023054	225	-
chr11	46690205	46690473	chr11:46690205:46690473:216023130	268	+
chr11	46690011	46690258	chr11:46690011:46690258:216023131	247	-
chr11	46690417	46690668	chr11:46690417:46690668:216023132	251	-
chr11	46677737	46677970	chr11:46677737:46677970:216023152	233	-
chr11	46678613	46678858	chr11:46678613:46678858:216023153	245	-
chr11	46666847	46667116	chr11:46666847:46667116:216023150	269	-
chr11	46690922	46691181	chr11:46690922:46691181:216023160	259	-
chr2	135596225	135596458	chr2:135596225:135596458:216023190	233	-
chr2	135602773	135603034	chr2:135602773:135603034:216023191	261	-
chr2	135619509	135619774	chr2:135619509:135619774:216023079	265	-
chr2	135621117	135621358	chr2:135621117:135621358:216023076	241	+
chr2	135620939	135621173	chr2:135620939:135621173:216023077	234	-
chr2	135630011	135630284	chr2:135630011:135630284:216023194	273	-
chr2	220086918	220087163	chr2:220086918:220087163:216023260	245	+
chr2	220087306	220087572	chr2:220087306:220087572:216023261	266	+
chr2	220086730	220086973	chr2:220086730:220086973:216023262	243	-
chr2	220087110	220087361	chr2:220087110:220087361:216023263	251	-
chr2	220087518	220087743	chr2:220087518:220087743:216023264	225	-
chr2	220092672	220092937	chr2:220092672:220092937:216023278	265	+
chr2	220092452	220092727	chr2:220092452:220092727:216023279	275	-
chr1	20964477	20964724	chr1:20964477:20964724:216023050	247	+
chr1	20964299	20964529	chr1:20964299:20964529:216023051	230	-
chr19	49458068	49458330	chr19:49458068:49458330:216023244	262	-
chr19	49464247	49464488	chr19:49464247:49464488:216023318	241	+
chr19	49464027	49464299	chr19:49464027:49464299:216023319	272	-
chr4	15713398	15713661	chr4:15713398:15713661:216023365	263	-
chr6	31616610	31616851	chr6:31616610:31616851:216023312	241	+
chr6	31616970	31617226	chr6:31616970:31617226:216023313	256	+
chr6	31617342	31617577	chr6:31617342:31617577:216023314	235	+
chr6	31616414	31616660	chr6:31616414:31616660:216023315	246	-
chr6	31616796	31617025	chr6:31616796:31617025:216023316	229	-
chr6	31617170	31617398	chr6:31617170:31617398:216023317	228	-
chr6	31612865	31613104	chr6:31612865:31613104:216023306	239	+
chr6	31613255	31613480	chr6:31613255:31613480:216023307	225	+
chr6	31612683	31612915	chr6:31612683:31612915:216023308	232	-
chr6	31613049	31613309	chr6:31613049:31613309:216023309	260	-
chr6	31610756	31611016	chr6:31610756:31611016:216023302	260	+
chr6	31610570	31610821	chr6:31610570:31610821:216023303	251	-
chr6	31607049	31607299	chr6:31607049:31607299:216023285	250	+
chr6	31606873	31607098	chr6:31606873:31607098:216023286	225	-

chr6	31607243	31607474	chr6:31607243:31607474:216023287	231	-
chr6	31619405	31619634	chr6:31619405:31619634:216023353	229	-
chr6	106634388	106634631	chr6:106634388:106634631:216023179	243	-
chr6	163148666	163148903	chr6:163148666:163148903:216022996	237	-
chr6	161771100	161771329	chr6:161771100:161771329:216022990	229	-
chr6	106649813	106650070	chr6:106649813:106650070:216023109	257	-
chr6	106740871	106741102	chr6:106740871:106741102:216023113	231	-
chr6	106756207	106756478	chr6:106756207:106756478:216023114	271	-
chr6	162475093	162475346	chr6:162475093:162475346:216022994	253	-
chr6	162206772	162207035	chr6:162206772:162207035:216022987	263	-
chr6	161781092	161781347	chr6:161781092:161781347:216022998	255	-
chr6	161807798	161808026	chr6:161807798:161808026:216022992	228	-
chr17	40962749	40963006	chr17:40962749:40963006:216023354	257	-
chr17	40967898	40968169	chr17:40967898:40968169:216023327	271	-
chr17	40971538	40971807	chr17:40971538:40971807:216023328	269	-
chr17	40975738	40975987	chr17:40975738:40975987:216023330	249	-
chr17	78091373	78091619	chr17:78091373:78091619:216023416	246	-
chr17	78092418	78092679	chr17:78092418:78092679:216023417	261	-
chr17	78091966	78092210	chr17:78091966:78092210:216023418	244	-
chr17	78079516	78079786	chr17:78079516:78079786:216023419	270	-
chr17	78084680	78084915	chr17:78084680:78084915:216023435	235	+
chr17	78084480	78084726	chr17:78084480:78084726:216023436	246	-
chr17	79667677	79667921	chr17:79667677:79667921:216023704	244	+
chr17	79668051	79668324	chr17:79668051:79668324:216023705	273	+
chr17	79667459	79667727	chr17:79667459:79667727:216023706	268	-
chr17	79667871	79668104	chr17:79667871:79668104:216023707	233	-
chr17	79661981	79662226	chr17:79661981:79662226:216023695	245	+
chr17	79661801	79662027	chr17:79661801:79662027:216023696	226	-
chr17	79662173	79662430	chr17:79662173:79662430:216023697	257	-
chr17	17119654	17119894	chr17:17119654:17119894:216023493	240	-
chr17	17125787	17126014	chr17:17125787:17126014:216023496	227	-
chr17	17129462	17129719	chr17:17129462:17129719:216023499	257	-
chr17	17131391	17131664	chr17:17131391:17131664:216023500	273	+
chr17	17131175	17131442	chr17:17131175:17131442:216023501	267	-
chr17	78078570	78078822	chr17:78078570:78078822:216023502	252	+
chr17	78078356	78078625	chr17:78078356:78078625:216023503	269	-
chr17	78078768	78079006	chr17:78078768:78079006:216023504	238	-
chr17	78081544	78081815	chr17:78081544:78081815:216023430	271	+
chr17	78081318	78081593	chr17:78081318:78081593:216023431	275	-
chr17	17118457	17118730	chr17:17118457:17118730:216023484	273	+
chr17	17118255	17118513	chr17:17118255:17118513:216023485	258	-
chr17	79652605	79652842	chr17:79652605:79652842:216023708	237	-
chr5	67589059	67589299	chr5:67589059:67589299:216023002	240	+
chr5	67589421	67589684	chr5:67589421:67589684:216023003	263	+
chr5	67588889	67589139	chr5:67588889:67589139:216023004	250	-
chr5	67589241	67589478	chr5:67589241:67589478:216023005	237	-
chr5	67589627	67589890	chr5:67589627:67589890:216023006	263	-

chr5	67590314	67590543	chr5:67590314:67590543:216023039	229	-
chr5	67522648	67522916	chr5:67522648:67522916:216023009	268	+
chr5	67522472	67522703	chr5:67522472:67522703:216023010	231	-
chr5	67584536	67584809	chr5:67584536:67584809:216023037	273	-
chr5	67593208	67593465	chr5:67593208:67593465:216023012	257	-
chr5	67569188	67569463	chr5:67569188:67569463:216023014	275	-
chr3	53217315	53217544	chr3:53217315:53217544:216023058	229	+
chr3	53217099	53217370	chr3:53217099:53217370:216023059	271	-
chr3	53217493	53217725	chr3:53217493:53217725:216023060	232	-
chr3	43740732	43740961	chr3:43740732:43740961:216023067	229	-
chr3	43743853	43744126	chr3:43743853:43744126:216023069	273	+
chr3	43743653	43743908	chr3:43743653:43743908:216023070	255	-
chr3	119595312	119595569	chr3:119595312:119595569:216023597	257	+
chr3	119595134	119595366	chr3:119595134:119595366:216023598	232	-
chr3	119634861	119635134	chr3:119634861:119635134:216023602	273	-
chr3	119642286	119642523	chr3:119642286:119642523:216023603	237	+
chr3	119642100	119642341	chr3:119642100:119642341:216023604	241	-
chr3	119666085	119666350	chr3:119666085:119666350:216023605	265	-
chr3	119812162	119812393	chr3:119812162:119812393:216023606	231	-
chr3	119562068	119562313	chr3:119562068:119562313:216023593	245	-
chr3	11348385	11348654	chr3:11348385:11348654:216023333	269	-
chr3	11383577	11383804	chr3:11383577:11383804:216023340	227	-
chr3	11372776	11373037	chr3:11372776:11373037:216023337	261	-
chr3	11374414	11374665	chr3:11374414:11374665:216023338	251	-
chr3	11404257	11404502	chr3:11404257:11404502:216023346	245	-
chr3	11406103	11406374	chr3:11406103:11406374:216023347	271	-
chr1	155209536	155209776	chr1:155209536:155209776:216023586	240	+
chr1	155209352	155209591	chr1:155209352:155209591:216023587	239	-
chr1	155209722	155209957	chr1:155209722:155209957:216023588	235	-
chr1	155206228	155206496	chr1:155206228:155206496:216023579	268	+
chr1	155206008	155206282	chr1:155206008:155206282:216023580	274	-
chr1	32797241	32797467	chr1:32797241:32797467:216023619	226	+
chr1	32797593	32797844	chr1:32797593:32797844:216023620	251	+
chr1	32797037	32797294	chr1:32797037:32797294:216023621	257	-
chr1	32797415	32797645	chr1:32797415:32797645:216023622	230	-
chr1	32797789	32798028	chr1:32797789:32798028:216023623	239	-
chr1	32792510	32792785	chr1:32792510:32792785:216023663	275	-
chr1	155204950	155205210	chr1:155204950:155205210:216023637	260	+
chr1	155204730	155205004	chr1:155204730:155205004:216023638	274	-
chr1	32782240	32782509	chr1:32782240:32782509:216023658	269	-
chr1	32794635	32794902	chr1:32794635:32794902:216023660	267	-
chr1	32768192	32768429	chr1:32768192:32768429:216023661	237	-
chr1	155210849	155211074	chr1:155210849:155211074:216023656	225	-
chr1	20966359	20966588	chr1:20966359:20966588:216023045	229	-
chr1	20971153	20971416	chr1:20971153:20971416:216023041	263	+
chr1	20970953	20971207	chr1:20970953:20971207:216023042	254	-
chr1	20972019	20972292	chr1:20972019:20972292:216023048	273	-

chr1	155207191	155207417	chr1:155207191:155207417:216023371	226	+
chr1	155207017	155207243	chr1:155207017:155207243:216023372	226	-
chr12	102163930	102164191	chr12:102163930:102164191:216023649	261	+
chr12	102164314	102164565	chr12:102164314:102164565:216023650	251	+
chr12	102163758	102163983	chr12:102163758:102163983:216023651	225	-
chr12	102164136	102164369	chr12:102164136:102164369:216023652	233	-
chr12	56109808	56110073	chr12:56109808:56110073:216023357	265	-
chr12	123282600	123282873	chr12:123282600:123282873:216023367	273	-
chr12	123270234	123270481	chr12:123270234:123270481:216023386	247	-
chr12	123259136	123259361	chr12:123259136:123259361:216023369	225	-
chr12	123262002	123262267	chr12:123262002:123262267:216023384	265	-
chr12	102151043	102151314	chr12:102151043:102151314:216023642	271	+
chr12	102150875	102151102	chr12:102150875:102151102:216023643	227	-
chr12	102151251	102151478	chr12:102151251:102151478:216023644	227	-
chr14	88454635	88454868	chr14:88454635:88454868:216023589	233	+
chr14	88454453	88454692	chr14:88454453:88454692:216023590	239	-
chr14	88454813	88455045	chr14:88454813:88455045:216023591	232	-
chr2	242607925	242608168	chr2:242607925:242608168:216023203	243	-
chr2	242594656	242594927	chr2:242594656:242594927:216023199	271	-
chr2	242598493	242598754	chr2:242598493:242598754:216023200	261	-
chr2	242606030	242606299	chr2:242606030:242606299:216023201	269	-
chr2	242577100	242577374	chr2:242577100:242577374:216023196	274	-
chr2	86732900	86733149	chr2:86732900:86733149:216023408	249	-
chr2	86734572	86734831	chr2:86734572:86734831:216023574	259	-
chr2	242610689	242610924	chr2:242610689:242610924:216023138	235	-
chr22	32894293	32894562	chr22:32894293:32894562:216023546	269	+
chr22	32894083	32894347	chr22:32894083:32894347:216023547	264	-
chr22	41488979	41489254	chr22:41488979:41489254:216023492	275	-
chr22	41569595	41569820	chr22:41569595:41569820:216023483	225	-
chr22	32875102	32875339	chr22:32875102:32875339:216023486	237	+
chr22	32874926	32875157	chr22:32874926:32875157:216023487	231	-
chr22	41565475	41565717	chr22:41565475:41565717:216023480	242	-
chr22	41554384	41554631	chr22:41554384:41554631:216023475	247	-
chr22	41564593	41564830	chr22:41564593:41564830:216023427	237	+
chr22	41564413	41564648	chr22:41564413:41564648:216023428	235	-
chr22	41564775	41565002	chr22:41564775:41565002:216023429	227	-
chr22	32883690	32883965	chr22:32883690:32883965:216023445	275	-
chr22	32889226	32889451	chr22:32889226:32889451:216023447	225	+
chr22	32889048	32889280	chr22:32889048:32889280:216023448	232	-
chr22	41543809	41544070	chr22:41543809:41544070:216023467	261	-
chr22	41545002	41545263	chr22:41545002:41545263:216023468	261	-
chr22	41513351	41513618	chr22:41513351:41513618:216023450	267	+
chr22	41513783	41514040	chr22:41513783:41514040:216023451	257	+
chr22	41513161	41513407	chr22:41513161:41513407:216023452	246	-
chr22	41513563	41513838	chr22:41513563:41513838:216023453	275	-
chr22	41525844	41526077	chr22:41525844:41526077:216023458	233	-
chr22	41527532	41527793	chr22:41527532:41527793:216023459	261	+

chr22	41527342	41527588	chr22:41527342:41527588:216023460	246	-
chr22	41531785	41532014	chr22:41531785:41532014:216023461	229	-
chr22	41533625	41533868	chr22:41533625:41533868:216023462	243	-
chr22	41536112	41536347	chr22:41536112:41536347:216023463	235	-
chr22	41537186	41537437	chr22:41537186:41537437:216023464	251	+
chr22	41537016	41537242	chr22:41537016:41537242:216023465	226	-
chr3	112269001	112269274	chr3:112269001:112269274:216023186	273	-
chr3	112272079	112272332	chr3:112272079:112272332:216023187	253	-
chr3	112262830	112263069	chr3:112262830:112263069:216023184	239	-
chr3	112251465	112251704	chr3:112251465:112251704:216023180	239	-
chr3	112253086	112253349	chr3:112253086:112253349:216023181	263	-
chr3	119720753	119720978	chr3:119720753:119720978:216023575	225	+
chr3	119720583	119720809	chr3:119720583:119720809:216023576	226	-
chr3	119720923	119721164	chr3:119720923:119721164:216023577	241	-
chr3	112256654	112256884	chr3:112256654:112256884:216023133	230	+
chr3	112256466	112256708	chr3:112256466:112256708:216023134	242	-
chr3	112256824	112257081	chr3:112256824:112257081:216023135	257	-
chr8	17928825	17929090	chr8:17928825:17929090:216023376	265	-
chr8	53573148	53573419	chr8:53573148:53573419:216023555	271	+
chr8	53573538	53573810	chr8:53573538:53573810:216023556	272	+
chr8	53573970	53574237	chr8:53573970:53574237:216023557	267	+
chr8	53572934	53573201	chr8:53572934:53573201:216023558	267	-
chr8	53573362	53573594	chr8:53573362:53573594:216023559	232	-
chr8	53573754	53574027	chr8:53573754:53574027:216023560	273	-
chr8	53574182	53574455	chr8:53574182:53574455:216023561	273	-
chr8	17919751	17920026	chr8:17919751:17920026:216023374	275	-
chr8	17941453	17941701	chr8:17941453:17941701:216023380	248	-
chr8	17924640	17924915	chr8:17924640:17924915:216023378	275	-
chr8	17927250	17927476	chr8:17927250:17927476:216023382	226	-
chr9	127999041	127999316	chr9:127999041:127999316:216023571	275	+
chr9	127998827	127999095	chr9:127998827:127999095:216023572	268	-
chr9	127999261	127999498	chr9:127999261:127999498:216023573	237	-
chr9	21968203	21968449	chr9:21968203:21968449:216023401	246	-
chr9	21968697	21968966	chr9:21968697:21968966:216023404	269	-
chr9	21971059	21971332	chr9:21971059:21971332:216023396	273	+
chr9	21970861	21971113	chr9:21970861:21971113:216023397	252	-
chr9	128000604	128000829	chr9:128000604:128000829:216022707	225	+
chr9	128000888	128001113	chr9:128000888:128001113:216022708	225	+
chr9	128001190	128001415	chr9:128001190:128001415:216022709	225	+
chr9	128001508	128001734	chr9:128001508:128001734:216022710	226	+
chr9	128000468	128000693	chr9:128000468:128000693:216022711	225	-
chr9	128000742	128000968	chr9:128000742:128000968:216022712	226	-
chr9	128001050	128001276	chr9:128001050:128001276:216022713	226	-
chr9	128001332	128001561	chr9:128001332:128001561:216022714	229	-
chrX	48664013	48664256	chrX:48664013:48664256:216023607	243	+
chrX	48663807	48664066	chrX:48663807:48664066:216023608	259	-
chrX	48678483	48678754	chrX:48678483:48678754:216023636	271	-

chrX	48666600	48666837	chrX:48666600:48666837:216023611	237	+
chrX	48666400	48666653	chrX:48666400:48666653:216023612	253	-
chrX	48673935	48674160	chrX:48673935:48674160:216023672	225	+
chrX	48674285	48674546	chrX:48674285:48674546:216023673	261	+
chrX	48674695	48674955	chrX:48674695:48674955:216023674	260	+
chrX	48673755	48673992	chrX:48673755:48673992:216023675	237	-
chrX	48674107	48674338	chrX:48674107:48674338:216023676	231	-
chrX	48674491	48674750	chrX:48674491:48674750:216023677	259	-
chrX	48674903	48675130	chrX:48674903:48675130:216023678	227	-
chrX	48681068	48681296	chrX:48681068:48681296:216023679	228	+
chrX	48681412	48681637	chrX:48681412:48681637:216023680	225	+
chrX	48681758	48681983	chrX:48681758:48681983:216023681	225	+
chrX	48682096	48682321	chrX:48682096:48682321:216023682	225	+
chrX	48682430	48682655	chrX:48682430:48682655:216023683	225	+
chrX	48682804	48683033	chrX:48682804:48683033:216023684	229	+
chrX	48680894	48681119	chrX:48680894:48681119:216023685	225	-
chrX	48681242	48681470	chrX:48681242:48681470:216023686	228	-
chrX	48681582	48681808	chrX:48681582:48681808:216023687	226	-
chrX	48681924	48682149	chrX:48681924:48682149:216023688	225	-
chrX	48682262	48682487	chrX:48682262:48682487:216023689	225	-
chrX	48682600	48682858	chrX:48682600:48682858:216023690	258	-
chrX	48682982	48683211	chrX:48682982:48683211:216023691	229	-
chr11	1774882	1775144	chr11:1774882:1775144:216023548	262	+
chr11	1775292	1775551	chr11:1775292:1775551:216023549	259	+
chr11	1774700	1774934	chr11:1774700:1774934:216023550	234	-
chr11	1775090	1775342	chr11:1775090:1775342:216023551	252	-
chr11	1784980	1785206	chr11:1784980:1785206:216023505	226	-
chr11	111782438	111782677	chr11:111782438:111782677:216023521	239	+
chr11	111782220	111782493	chr11:111782220:111782493:216023522	273	-
chr11	1782511	1782742	chr11:1782511:1782742:216023413	231	-
chr11	1776108	1776381	chr11:1776108:1776381:216023421	273	-
chr11	46693777	46694043	chr11:46693777:46694043:216023162	266	-
chr11	46685513	46685768	chr11:46685513:46685768:216023156	255	-
chr16	730608	730862	chr16:730608:730862:216024044	254	+
chr16	730410	730658	chr16:730410:730658:216024045	248	-
chr4	90650315	90650576	chr4:90650315:90650576:216023991	261	-
chr4	15704742	15704998	chr4:15704742:15704998:216023359	256	-
chr4	15724412	15724677	chr4:15724412:15724677:216023363	265	-
chr4	15717290	15717555	chr4:15717290:15717555:216023361	265	-
chr4	3127240	3127498	chr4:3127240:3127498:216022839	258	-
chr4	3100965	3101200	chr4:3100965:3101200:216022720	235	-
chr4	3190636	3190875	chr4:3190636:3190875:216022724	239	-
chr4	3148494	3148725	chr4:3148494:3148725:216022840	231	-
chr4	3225107	3225337	chr4:3225107:3225337:216022722	230	-
chr17	79657676	79657935	chr17:79657676:79657935:216023754	259	-
chr17	79668508	79668763	chr17:79668508:79668763:216023752	255	-
chr13	113975857	113976114	chr13:113975857:113976114:216022853	257	+

chr13	113975679	113975907	chr13:113975679:113975907:216022854	228	-
chr13	113973934	113974163	chr13:113973934:113974163:216022858	229	+
chr13	113973756	113973988	chr13:113973756:113973988:216022859	232	-
chr13	113974632	113974899	chr13:113974632:113974899:216022860	267	-
chr13	113951718	113951979	chr13:113951718:113951979:216022855	261	-
chr12	40671808	40672046	chr12:40671808:40672046:216022847	238	+
chr12	40671636	40671866	chr12:40671636:40671866:216022848	230	-
chr12	40671992	40672259	chr12:40671992:40672259:216022849	267	-
chr12	40645096	40645361	chr12:40645096:40645361:216022841	265	+
chr12	40644916	40645151	chr12:40644916:40645151:216022842	235	-
chr12	40645306	40645535	chr12:40645306:40645535:216022843	229	-
chr12	40742169	40742422	chr12:40742169:40742422:216022816	253	-
chr12	40704207	40704478	chr12:40704207:40704478:216022802	271	-
chr12	40707742	40708013	chr12:40707742:40708013:216022803	271	-
chr12	40687301	40687568	chr12:40687301:40687568:216022790	267	-
chr12	123307881	123308110	chr12:123307881:123308110:216023391	229	-
chr12	123281806	123282031	chr12:123281806:123282031:216023389	225	-
chr12	123332550	123332813	chr12:123332550:123332813:216023798	263	-
chr12	123343421	123343647	chr12:123343421:123343647:216023774	226	+
chr12	123343761	123343986	chr12:123343761:123343986:216023775	225	+
chr12	123344101	123344326	chr12:123344101:123344326:216023776	225	+
chr12	123344413	123344638	chr12:123344413:123344638:216023777	225	+
chr12	123344729	123344955	chr12:123344729:123344955:216023778	226	+
chr12	123345037	123345265	chr12:123345037:123345265:216023779	228	+
chr12	123345351	123345576	chr12:123345351:123345576:216023780	225	+
chr12	123345681	123345928	chr12:123345681:123345928:216023781	247	+
chr12	123346055	123346299	chr12:123346055:123346299:216023782	244	+
chr12	123343255	123343481	chr12:123343255:123343481:216023783	226	-
chr12	123343589	123343814	chr12:123343589:123343814:216023784	225	-
chr12	123343931	123344157	chr12:123343931:123344157:216023785	226	-
chr12	123344245	123344472	chr12:123344245:123344472:216023786	227	-
chr12	123344577	123344803	chr12:123344577:123344803:216023787	226	-
chr12	123344893	123345118	chr12:123344893:123345118:216023788	225	-
chr12	123345183	123345409	chr12:123345183:123345409:216023789	226	-
chr12	123345509	123345734	chr12:123345509:123345734:216023790	225	-
chr12	123345873	123346107	chr12:123345873:123346107:216023791	234	-
chr12	123346245	123346504	chr12:123346245:123346504:216023792	259	-
chr12	123339498	123339724	chr12:123339498:123339724:216023758	226	+
chr12	123339856	123340087	chr12:123339856:123340087:216023759	231	+
chr12	123340204	123340429	chr12:123340204:123340429:216023760	225	+
chr12	123340544	123340770	chr12:123340544:123340770:216023761	226	+
chr12	123340896	123341122	chr12:123340896:123341122:216023762	226	+
chr12	123341244	123341487	chr12:123341244:123341487:216023763	243	+
chr12	123341636	123341893	chr12:123341636:123341893:216023764	257	+
chr12	123339326	123339552	chr12:123339326:123339552:216023765	226	-
chr12	123339676	123339902	chr12:123339676:123339902:216023766	226	-
chr12	123340034	123340262	chr12:123340034:123340262:216023767	228	-

chr12	123340372	123340597	chr12:123340372:123340597:216023768	225	-
chr12	123340714	123340946	chr12:123340714:123340946:216023769	232	-
chr12	123341072	123341297	chr12:123341072:123341297:216023770	225	-
chr12	123341436	123341687	chr12:123341436:123341687:216023771	251	-
chr12	123335739	123335965	chr12:123335739:123335965:216023796	226	-
chr12	123338554	123338789	chr12:123338554:123338789:216023794	235	-
chr12	50156624	50156871	chr12:50156624:50156871:216024073	247	-
chr12	50146724	50146951	chr12:50146724:50146951:216024070	227	-
chr4	90756668	90756927	chr4:90756668:90756927:216024011	259	-
chr4	3227362	3227588	chr4:3227362:3227588:216023860	226	-
chr4	3161981	3162208	chr4:3161981:3162208:216023857	227	-
chr4	844820	845091	chr4:844820:845091:216023624	271	+
chr4	844606	844874	chr4:844606:844874:216023625	268	-
chr4	845692	845931	chr4:845692:845931:216023506	239	+
chr4	845510	845738	chr4:845510:845738:216023507	228	-
chr4	871557	871822	chr4:871557:871822:216023511	265	+
chr4	871375	871608	chr4:871375:871608:216023512	233	-
chr4	864471	864726	chr4:864471:864726:216023629	255	-
chr4	870290	870554	chr4:870290:870554:216023509	264	-
chr4	925787	926051	chr4:925787:926051:216023634	264	-
chr4	898397	898668	chr4:898397:898668:216023633	271	-
chr4	905433	905708	chr4:905433:905708:216023518	275	-
chr4	877801	878046	chr4:877801:878046:216023630	245	-
chr4	882595	882821	chr4:882595:882821:216023516	226	-
chr4	860932	861195	chr4:860932:861195:216023488	263	+
chr4	860716	860986	chr4:860716:860986:216023489	270	-
chr4	861142	861376	chr4:861142:861376:216023490	234	-
chr4	887137	887400	chr4:887137:887400:216023491	263	-
chr4	887634	887898	chr4:887634:887898:216023592	264	-
chr4	3142205	3142448	chr4:3142205:3142448:216022741	243	-
chr4	3144551	3144792	chr4:3144551:3144792:216022735	241	+
chr4	3144367	3144606	chr4:3144367:3144606:216022736	239	-
chr4	3123139	3123374	chr4:3123139:3123374:216022744	235	+
chr4	3122925	3123196	chr4:3122925:3123196:216022745	271	-
chr4	3124584	3124839	chr4:3124584:3124839:216022731	255	-
chr4	3116999	3117228	chr4:3116999:3117228:216022726	229	-
chr4	3231588	3231840	chr4:3231588:3231840:216022739	252	-
chr4	3215655	3215918	chr4:3215655:3215918:216022742	263	-
chr4	3216803	3217036	chr4:3216803:3217036:216022757	233	-
chr4	3156074	3156327	chr4:3156074:3156327:216022759	253	+
chr4	3155898	3156128	chr4:3155898:3156128:216022760	230	-
chr4	3211516	3211753	chr4:3211516:3211753:216022751	237	-
chr4	3213806	3214073	chr4:3213806:3214073:216022762	267	+
chr4	3213624	3213859	chr4:3213624:3213859:216022763	235	-
chr4	3205704	3205953	chr4:3205704:3205953:216022733	249	-
chr4	3188292	3188547	chr4:3188292:3188547:216022754	255	-
chr4	3189560	3189797	chr4:3189560:3189797:216022728	237	+

chr4	3189344	3189615	chr4:3189344:3189615:216022729	271	-
chr4	3241729	3241963	chr4:3241729:3241963:216022748	234	+
chr4	3241545	3241781	chr4:3241545:3241781:216022749	236	-
chr10	73579400	73579671	chr10:73579400:73579671:216023871	271	+
chr10	73579188	73579448	chr10:73579188:73579448:216023872	260	-
chr10	73579616	73579859	chr10:73579616:73579859:216023873	243	-
chr10	73585553	73585806	chr10:73585553:73585806:216023875	253	-
chr10	73588606	73588867	chr10:73588606:73588867:216023806	261	-
chr10	73578760	73579029	chr10:73578760:73579029:216023803	269	-
chr2	168996939	168997209	chr2:168996939:168997209:216023988	270	+
chr2	168996759	168996994	chr2:168996759:168996994:216023989	235	-
chr2	168997155	168997382	chr2:168997155:168997382:216023990	227	-
chr2	168931656	168931907	chr2:168931656:168931907:216023984	251	+
chr2	168931436	168931710	chr2:168931436:168931710:216023985	274	-
chr2	168821142	168821385	chr2:168821142:168821385:216024013	243	-
chr2	168873509	168873742	chr2:168873509:168873742:216024015	233	-
chr2	168919974	168920247	chr2:168919974:168920247:216024016	273	-
chr2	168921799	168922054	chr2:168921799:168922054:216024017	255	-
chr2	169023775	169024010	chr2:169023775:169024010:216024021	235	-
chr2	169038460	169038717	chr2:169038460:169038717:216024022	257	-
chr2	86756301	86756562	chr2:86756301:86756562:216023406	261	-
chr3	11382079	11382304	chr3:11382079:11382304:216023339	225	-
chr3	11389323	11389592	chr3:11389323:11389592:216023341	269	-
chr3	182841830	182842091	chr3:182841830:182842091:216022893	261	-
chr3	182870135	182870388	chr3:182870135:182870388:216022896	253	-
chr3	182871660	182871895	chr3:182871660:182871895:216022897	235	+
chr3	182872014	182872262	chr3:182872014:182872262:216022898	248	+
chr3	182871436	182871709	chr3:182871436:182871709:216022899	273	-
chr3	182871842	182872068	chr3:182871842:182872068:216022900	226	-
chr3	53222914	53223148	chr3:53222914:53223148:216023889	234	+
chr3	53222700	53222967	chr3:53222700:53222967:216023890	267	-
chr3	53223092	53223354	chr3:53223092:53223354:216023891	262	-
chr3	53213769	53214034	chr3:53213769:53214034:216023811	265	+
chr3	53213567	53213825	chr3:53213567:53213825:216023812	258	-
chr3	53221328	53221561	chr3:53221328:53221561:216023814	233	-
chr3	53223863	53224100	chr3:53223863:53224100:216023815	237	-
chr6	74325001	74325250	chr6:74325001:74325250:216024076	249	-
chr6	74345162	74345387	chr6:74345162:74345387:216024077	225	+
chr6	74344988	74345217	chr6:74344988:74345217:216024078	229	-
chr6	74346235	74346510	chr6:74346235:74346510:216024079	275	-
chr6	74351565	74351817	chr6:74351565:74351817:216024082	252	+
chr6	74351377	74351619	chr6:74351377:74351619:216024083	242	-
chr6	74310003	74310228	chr6:74310003:74310228:216024074	225	-
chr8	98837254	98837499	chr8:98837254:98837499:216022891	245	-
chr8	98828252	98828477	chr8:98828252:98828477:216022889	225	-
chr8	98788049	98788317	chr8:98788049:98788317:216022884	268	+
chr8	98787851	98788095	chr8:98787851:98788095:216022885	244	-

chr8	98788269	98788530	chr8:98788269:98788530:216022886	261	-
chr8	98817529	98817762	chr8:98817529:98817762:216022887	233	-
chrX	119575457	119575688	chrX:119575457:119575688:216022705	231	-
chrX	119562462	119562697	chrX:119562462:119562697:216022874	235	+
chrX	119562274	119562517	chrX:119562274:119562517:216022875	243	-
chrX	119565149	119565410	chrX:119565149:119565410:216022876	261	-
chrX	119572980	119573215	chrX:119572980:119573215:216022877	235	-
chrX	119602933	119603189	chrX:119602933:119603189:216022861	256	-
chrX	119589356	119589605	chrX:119589356:119589605:216022769	249	+
chrX	119589180	119589411	chrX:119589180:119589411:216022770	231	-
chr11	6412794	6413067	chr11:6412794:6413067:216023994	273	+
chr11	6413212	6413487	chr11:6413212:6413487:216023995	275	+
chr11	6412576	6412849	chr11:6412576:6412849:216023996	273	-
chr11	6413016	6413265	chr11:6413016:6413265:216023997	249	-
chr11	6412015	6412282	chr11:6412015:6412282:216024000	267	+
chr11	6411801	6412062	chr11:6411801:6412062:216024001	261	-
chr1	203834127	203834392	chr1:203834127:203834392:216024034	265	-
chr1	203838973	203839242	chr1:203838973:203839242:216024035	269	-
chr14	88452798	88453059	chr14:88452798:88453059:216023715	261	-
chr14	88442665	88442938	chr14:88442665:88442938:216023720	273	-
chr14	88448454	88448681	chr14:88448454:88448681:216023713	227	-
chr14	88411864	88412133	chr14:88411864:88412133:216023725	269	-
chr14	88401042	88401301	chr14:88401042:88401301:216023710	259	-
chr14	88429691	88429966	chr14:88429691:88429966:216023718	275	-
chr14	88431814	88432054	chr14:88431814:88432054:216023722	240	-
chr14	68123105	68123332	chr14:68123105:68123332:216022718	227	-
chr10	73577173	73577406	chr10:73577173:73577406:216023971	233	-
chr10	73591571	73591818	chr10:73591571:73591818:216023809	247	-
chr10	73579971	73580241	chr10:73579971:73580241:216023804	270	-
chr1	87194031	87194257	chr1:87194031:87194257:216023962	226	-
chr1	87208055	87208327	chr1:87208055:87208327:216024028	272	+
chr1	87207859	87208112	chr1:87207859:87208112:216024029	253	-
chr1	87208695	87208942	chr1:87208695:87208942:216024030	247	-
chr1	87195721	87195952	chr1:87195721:87195952:216023983	231	-
chr1	87200239	87200474	chr1:87200239:87200474:216024026	235	-
chr1	155850249	155850504	chr1:155850249:155850504:216024061	255	-
chr1	155829523	155829772	chr1:155829523:155829772:216024055	249	-
chr12	50146205	50146454	chr12:50146205:50146454:216024051	249	-
chr12	102140908	102141171	chr12:102140908:102141171:216023732	263	-
chr12	102142832	102143091	chr12:102142832:102143091:216023729	259	-
chr12	102224298	102224533	chr12:102224298:102224533:216023744	235	-
chr12	102158119	102158390	chr12:102158119:102158390:216023734	271	+
chr12	102158555	102158816	chr12:102158555:102158816:216023735	261	+
chr12	102158953	102159216	chr12:102158953:102159216:216023736	263	+
chr12	102157945	102158174	chr12:102157945:102158174:216023737	229	-
chr12	102158335	102158610	chr12:102158335:102158610:216023738	275	-
chr12	102158761	102159008	chr12:102158761:102159008:216023739	247	-

chr12	102161774	102162000	chr12:102161774:102162000:216023741	226	-
chr12	102164741	102164992	chr12:102164741:102164992:216023727	251	-
chr17	79653310	79653545	chr17:79653310:79653545:216023748	235	-
chr17	79658449	79658676	chr17:79658449:79658676:216023746	227	-
chr17	79662984	79663259	chr17:79662984:79663259:216023750	275	+
chr17	79662792	79663032	chr17:79662792:79663032:216023751	240	-
chr4	3240317	3240582	chr4:3240317:3240582:216023846	265	+
chr4	3240119	3240372	chr4:3240119:3240372:216023847	253	-
chr4	3240527	3240776	chr4:3240527:3240776:216023848	249	-
chr4	3208354	3208580	chr4:3208354:3208580:216023835	226	+
chr4	3208746	3208995	chr4:3208746:3208995:216023836	249	+
chr4	3208168	3208409	chr4:3208168:3208409:216023837	241	-
chr4	3208526	3208800	chr4:3208526:3208800:216023838	274	-
chr4	3208940	3209197	chr4:3208940:3209197:216023839	257	-
chr4	3230449	3230675	chr4:3230449:3230675:216023840	226	+
chr4	3230283	3230509	chr4:3230283:3230509:216023841	226	-
chr4	3230621	3230852	chr4:3230621:3230852:216023842	231	-
chr4	3133142	3133386	chr4:3133142:3133386:216023823	244	+
chr4	3132958	3133198	chr4:3132958:3133198:216023824	240	-
chr4	3133332	3133597	chr4:3133332:3133597:216023825	265	-
chr4	3174015	3174287	chr4:3174015:3174287:216023858	272	-
chr8	53555069	53555308	chr8:53555069:53555308:216023920	239	+
chr8	53554871	53555124	chr8:53554871:53555124:216023921	253	-
chr8	53555253	53555482	chr8:53555253:53555482:216023922	229	-
chr8	53586573	53586826	chr8:53586573:53586826:216023946	253	+
chr8	53586373	53586626	chr8:53586373:53586626:216023947	253	-
chr8	53586771	53587022	chr8:53586771:53587022:216023948	251	-
chr8	53537243	53537502	chr8:53537243:53537502:216023949	259	-
chr8	53596179	53596444	chr8:53596179:53596444:216023937	265	+
chr8	53596007	53596234	chr8:53596007:53596234:216023938	227	-
chr8	53596389	53596640	chr8:53596389:53596640:216023939	251	-
chr8	53542988	53543218	chr8:53542988:53543218:216023940	230	-
chr8	53548533	53548790	chr8:53548533:53548790:216023941	257	-
chr8	53558392	53558635	chr8:53558392:53558635:216023942	243	+
chr8	53558222	53558447	chr8:53558222:53558447:216023943	225	-
chr9	128003080	128003339	chr9:128003080:128003339:216023963	259	+
chr9	128002898	128003134	chr9:128002898:128003134:216023964	236	-
chr9	128003282	128003528	chr9:128003282:128003528:216023965	246	-
chr14	102552233	102552460	chr14:102552233:102552460:216023901	227	+
chr14	102552583	102552838	chr14:102552583:102552838:216023902	255	+
chr14	102552057	102552287	chr14:102552057:102552287:216023903	230	-
chr14	102552405	102552637	chr14:102552405:102552637:216023904	232	-
chr14	102549503	102549746	chr14:102549503:102549746:216023906	243	+
chr14	102549897	102550151	chr14:102549897:102550151:216023907	254	+
chr14	102550271	102550542	chr14:102550271:102550542:216023908	271	+
chr14	102549331	102549558	chr14:102549331:102549558:216023909	227	-
chr14	102549691	102549952	chr14:102549691:102549952:216023910	261	-

chr14	102550093	102550326	chr14:102550093:102550326:216023911	233	-
chr14	102568390	102568661	chr14:102568390:102568661:216023894	271	+
chr14	102568178	102568445	chr14:102568178:102568445:216023895	267	-
chr14	73673064	73673317	chr14:73673064:73673317:216023868	253	-
chr14	73678447	73678698	chr14:73678447:73678698:216023869	251	-
chr14	73640242	73640473	chr14:73640242:73640473:216023863	231	-
chr14	73653523	73653762	chr14:73653523:73653762:216023864	239	-
chr14	73659434	73659681	chr14:73659434:73659681:216023865	247	+
chr14	73659264	73659489	chr14:73659264:73659489:216023866	225	-
chr2	65324995	65325252	chr2:65324995:65325252:216023978	257	-
chr2	65331806	65332053	chr2:65331806:65332053:216023979	247	-
chr3	128514179	128514452	chr3:128514179:128514452:216023852	273	-
chr3	128516754	128517001	chr3:128516754:128517001:216023816	247	-
chr3	128532142	128532407	chr3:128532142:128532407:216023953	265	-
chr4	3134408	3134635	chr4:3134408:3134635:216023826	227	+
chr4	3134232	3134463	chr4:3134232:3134463:216023827	231	-
chr4	3134580	3134821	chr4:3134580:3134821:216023828	241	-
chr6	32486307	32486564	chr6:32486307:32486564:216023801	257	-
chr6	32487315	32487575	chr6:32487315:32487575:216023849	260	+
chr6	32487115	32487369	chr6:32487115:32487369:216023850	254	-
chr6	41658571	41658830	chr6:41658571:41658830:216024048	259	+
chr6	41658365	41658625	chr6:41658365:41658625:216024049	260	-
chr6	41658781	41659023	chr6:41658781:41659023:216024050	242	-
chr6	41654804	41655057	chr6:41654804:41655057:216024066	253	-
chr6	41652532	41652759	chr6:41652532:41652759:216024062	227	+
chr6	41652310	41652582	chr6:41652310:41652582:216024063	272	-
chr6	41652704	41652973	chr6:41652704:41652973:216024064	269	-
chr11	122931980	122932209	chr11:122931980:122932209:216023972	229	+
chr11	122931796	122932032	chr11:122931796:122932032:216023973	236	-
chr11	122928573	122928828	chr11:122928573:122928828:216023958	255	+
chr11	122928403	122928628	chr11:122928403:122928628:216023959	225	-
chr12	40702383	40702610	chr12:40702383:40702610:216022862	227	+
chr12	40702183	40702437	chr12:40702183:40702437:216022863	254	-
chr12	40637304	40637529	chr12:40637304:40637529:216022778	225	-
chr12	40760694	40760931	chr12:40760694:40760931:216022827	237	-
chr16	87432387	87432624	chr16:87432387:87432624:216022881	237	-
chr16	87435749	87436024	chr16:87435749:87436024:216022882	275	-
chr16	56624722	56624987	chr16:56624722:56624987:216022986	265	-
chr20	33147196	33147471	chr20:33147196:33147471:216022870	275	+
chr20	33147618	33147851	chr20:33147618:33147851:216022871	233	+
chr20	33146976	33147248	chr20:33146976:33147248:216022872	272	-
chr20	33147418	33147674	chr20:33147418:33147674:216022873	256	-
chr20	33146599	33146833	chr20:33146599:33146833:216022879	234	-
chr3	182804453	182804708	chr3:182804453:182804708:216022948	255	-
chr3	182810167	182810428	chr3:182810167:182810428:216022949	261	-
chr3	182775067	182775308	chr3:182775067:182775308:216022946	241	-
chr3	182759323	182759598	chr3:182759323:182759598:216022943	275	-

chr3	182743513	182743774	chr3:182743513:182743774:216022938	261	-
chr17	44067404	44067659	chr17:44067404:44067659:216022957	255	+
chr17	44067196	44067459	chr17:44067196:44067459:216022958	263	-
chr17	44051723	44051998	chr17:44051723:44051998:216022955	275	-
chr17	44055713	44055974	chr17:44055713:44055974:216022828	261	-
chr17	44071258	44071507	chr17:44071258:44071507:216022959	249	-
chr17	44087634	44087883	chr17:44087634:44087883:216022835	249	-
chr17	44091581	44091832	chr17:44091581:44091832:216022962	251	-
chr17	44095954	44096197	chr17:44095954:44096197:216022963	243	-
chr19	7587601	7587860	chr19:7587601:7587860:216022964	259	-
chr19	7594444	7594669	chr19:7594444:7594669:216022923	225	-
chr19	7591465	7591718	chr19:7591465:7591718:216022966	253	+
chr19	7591289	7591515	chr19:7591289:7591515:216022967	226	-
chr19	7591661	7591887	chr19:7591661:7591887:216022968	226	-
chr19	7593647	7593901	chr19:7593647:7593901:216022973	254	+
chr19	7594057	7594292	chr19:7594057:7594292:216022974	235	+
chr19	7593449	7593695	chr19:7593449:7593695:216022975	246	-
chr19	7593845	7594111	chr19:7593845:7594111:216022976	266	-
chr12	40643596	40643857	chr12:40643596:40643857:216022779	261	-
chr12	40634365	40634630	chr12:40634365:40634630:216022776	265	+
chr12	40634171	40634420	chr12:40634171:40634420:216022777	249	-
chr12	40626044	40626269	chr12:40626044:40626269:216022773	225	-
chr12	40629396	40629638	chr12:40629396:40629638:216022774	242	-
chr12	40689387	40689658	chr12:40689387:40689658:216022792	271	+
chr12	40689197	40689441	chr12:40689197:40689441:216022793	244	-
chr12	40692221	40692472	chr12:40692221:40692472:216022794	251	+
chr12	40692001	40692275	chr12:40692001:40692275:216022795	274	-
chr12	40653250	40653513	chr12:40653250:40653513:216022784	263	-
chr12	40657507	40657773	chr12:40657507:40657773:216022785	266	-
chr12	40677789	40678064	chr12:40677789:40678064:216022786	275	+
chr12	40677603	40677845	chr12:40677603:40677845:216022787	242	-
chr12	40714806	40715079	chr12:40714806:40715079:216022807	273	-
chr12	40717058	40717331	chr12:40717058:40717331:216022808	273	+
chr12	40716890	40717115	chr12:40716890:40717115:216022809	225	-
chr12	40748225	40748458	chr12:40748225:40748458:216022819	233	+
chr12	40748055	40748280	chr12:40748055:40748280:216022820	225	-
chr12	40740687	40740912	chr12:40740687:40740912:216022814	225	+
chr12	40740509	40740745	chr12:40740509:40740745:216022815	236	-
chr18	21136391	21136666	chr18:21136391:21136666:216022902	275	+
chr18	21136175	21136443	chr18:21136175:21136443:216022903	268	-
chr18	21152010	21152279	chr18:21152010:21152279:216022917	269	-
chr18	21120330	21120559	chr18:21120330:21120559:216022915	229	-
chr18	21124278	21124541	chr18:21124278:21124541:216022911	263	-
chr18	21127942	21128185	chr18:21127942:21128185:216022912	243	-
chr18	21114382	21114613	chr18:21114382:21114613:216022913	231	-
chr18	21119953	21120212	chr18:21119953:21120212:216022907	259	+
chr18	21119745	21120008	chr18:21119745:21120008:216022908	263	-

chr18	21137049	21137288	chr18:21137049:21137288:216022909	239	-
chr18	21115599	21115835	chr18:21115599:21115835:216022926	236	+
chr18	21115401	21115654	chr18:21115401:21115654:216022927	253	-
chr18	21141292	21141521	chr18:21141292:21141521:216022928	229	-
chr18	21153386	21153613	chr18:21153386:21153613:216022929	227	-
chr18	21140365	21140614	chr18:21140365:21140614:216022930	249	+
chr18	21140165	21140418	chr18:21140165:21140418:216022931	253	-
chr3	182755198	182755433	chr3:182755198:182755433:216022940	235	+
chr3	182754976	182755250	chr3:182754976:182755250:216022941	274	-
chr3	182735022	182735249	chr3:182735022:182735249:216022935	227	-
chr3	182769915	182770164	chr3:182769915:182770164:216022945	249	-
